# Supplementary figures and images for: New enantiornithine diversity in the Hell Creek Formation and the functional morphology of the avisaurid tarsometatarsus
Source: PLoS One. 2024 Oct 9;19(10):e0310686. doi: 10.1371/journal.pone.0310686 (PMC11463745; doi:10.1371/journal.pone.0310686)

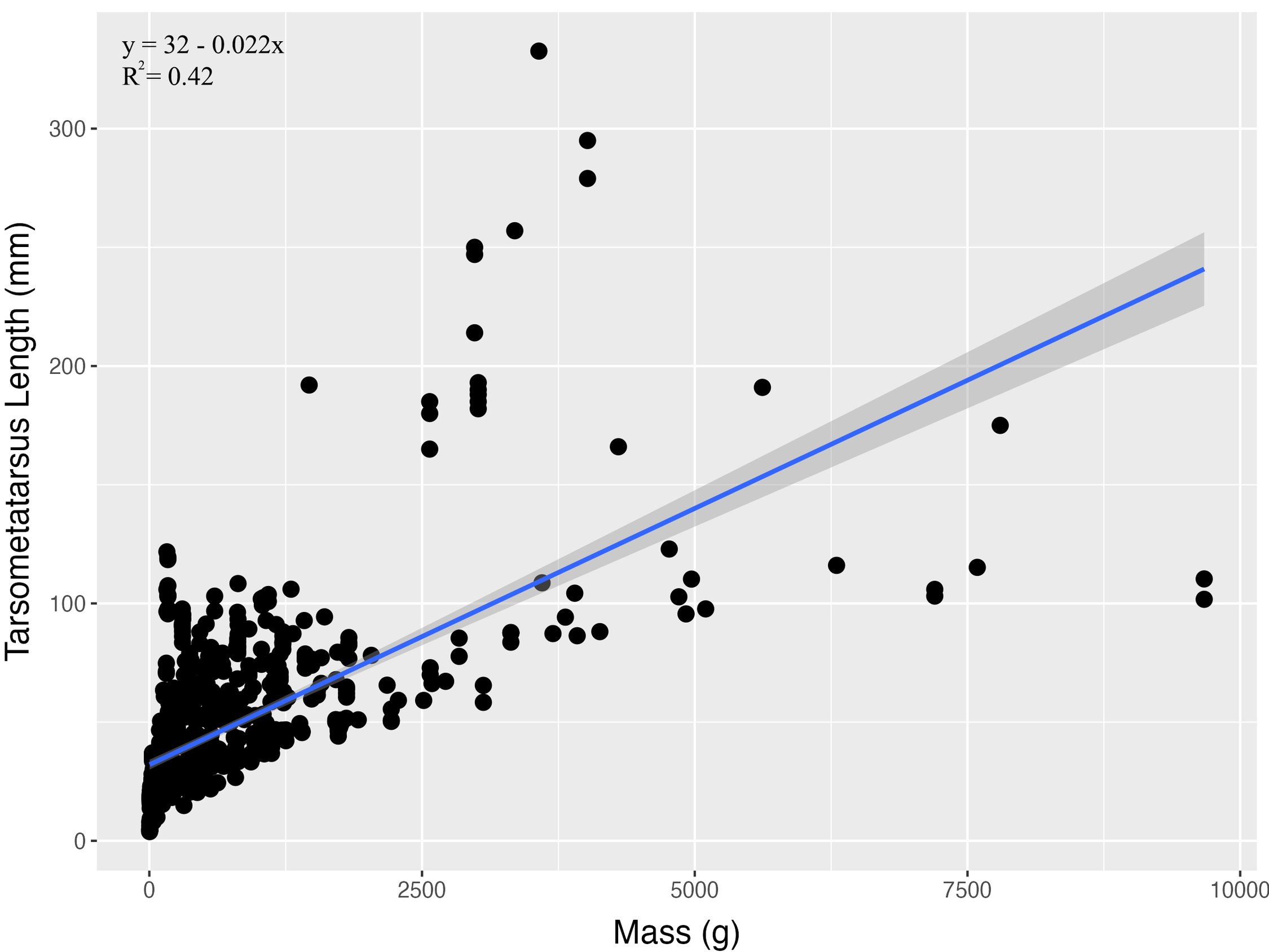

Supplement: S1 Fig — Data from Field et al., (2013). (TIF) [file pone.0310686.s001.tif]

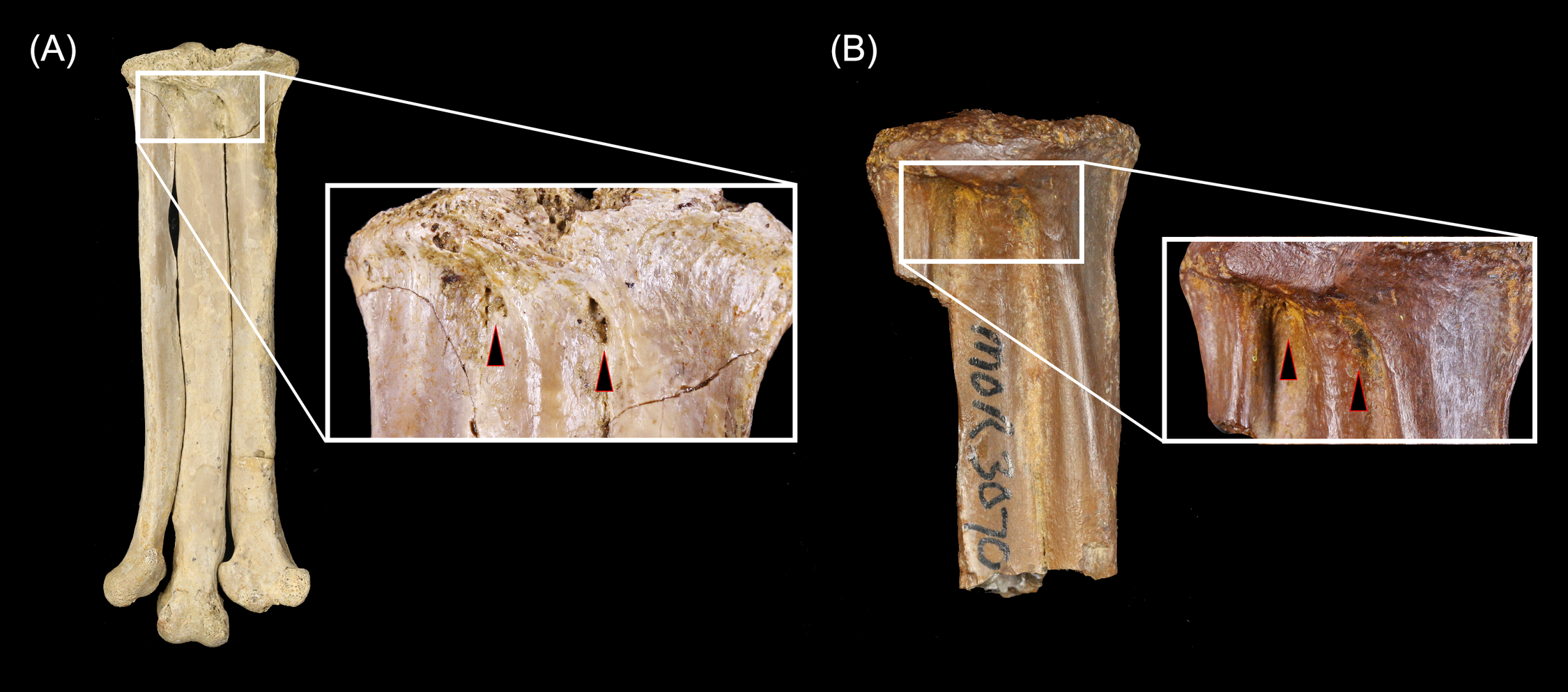

Supplement: S2 Fig — Close-up photos of the proximoplantar fossa on the plantar face of (A) Avisaurus darwini, and (B) Avisaurus sp. MOR 3070. The depressions are indicated by the black arrows. (TIF) [file pone.0310686.s002.tif]

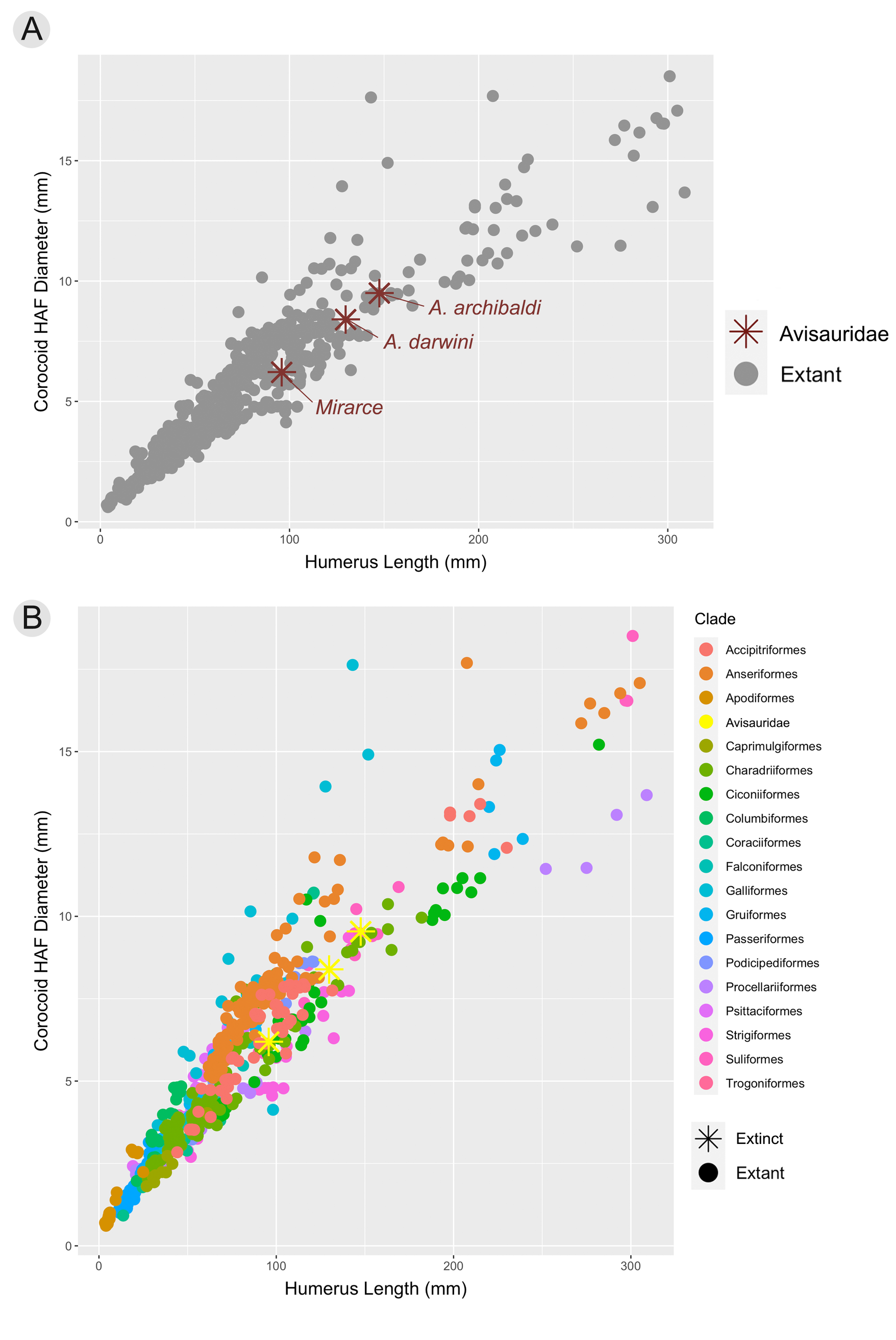

Supplement: S3 Fig — Utilizing the dataset from Field et al., (2013), three avisaurids (A. archibaldi, A. darwini, Mirarce) were plotted against (A) all sampled extant birds, and then against all birds, (B) by family. Once closely associated families were identified, an averaged mass estimate could be calculated using data collected within Field et al., (2013). (TIF) [file pone.0310686.s003.tif]
