# Supplementary material for: New enantiornithine diversity in the Hell Creek Formation and the functional morphology of the avisaurid tarsometatarsus
Source: PLoS One. 2024 Oct 9;19(10):e0310686. doi: 10.1371/journal.pone.0310686 (PMC11463745; doi:10.1371/journal.pone.0310686)
Supplement: Attachment — Submitted filename: PONE-D-24-29019_reviewer.pdf [file pone.0310686.s008.pdf]

PLOS ONE

New enantiornithine diversity in the Hell Creek and the functional morphology of the avisaurid tarsometatarsus

--Manuscript Draft--

**Manuscript Number:**	PONE-D-24-29019
**Article Type:**	Research Article
**Full Title:**	New enantiornithine diversity in the Hell Creek and the functional morphology of the avisaurid tarsometatarsus
**Short Title:**	New Hell Creek enantiornithines and avisaurid tarsometatarsi functional morphology
**Corresponding Author:**	Alexander Clark, M.S.<br>University of Chicago Division of the Biological Sciences<br>Chicago, UNITED STATES OF AMERICA
**Keywords:**	Enantiornithes, paleobiology, Hell Creek Formation, paleoecology, Mesozoic birds
**Abstract:**	Enantiornithines were the most diverse group of birds during the Cretaceous, comprising over half of all known species from this period. The fossil record and subsequently our knowledge of this clade is heavily skewed by the wealth of material from Lower Cretaceous deposits in China. In contrast, specimens from Upper Cretaceous deposits are rare and typically fragmentary, yet critical for understanding the extinction of this clade across the K-Pg boundary. The most complete North American Late Cretaceous enantiornithine is Mirarce eatoni, a member of the diverse clade Avisauridae. Except for Mirarce, avisaurids are known only from isolated hindlimb elements from North and South America. Here we describe three new enantiornithines from the Maastrichtian Hell Creek Formation, two of which represent new avisaurid taxa. These materials represent a substantial increase in the known diversity of Enantiornithes in the latest Cretaceous. Re-examination of material referred to Avisauridae through phylogenetic analysis provides strong support for a more exclusive Avisauridae consisting of six taxa. Exploration of the functional morphology of the avisaurid tarsometatarsus indicates strong grip and raptorial attributes. The lower aspect ratio of the tarsometatarsus facilitates a more biomechanically efficient lever system for lifting proportionally heavier objects. In addition, the proportional size and distal position of the m. tibialis cranialis tubercle of the tarsometatarsus is similar to the morphology seen in extant birds of prey. Together with the deeply-grooved metatarsal trochlea facilitating robust and likely powerful pedal digits, morphologies of the hindlimb suggest avisaurids as Late Cretaceous birds of prey.
**Order of Authors:**	Alexander Clark, M.S.<br>Jessie Atterholt<br>John Scanella<br>Thomas Carr<br>Nathan Carrol<br>Jingmai O'Connor, PhD
**Additional Information:**	
**Question**	**Response**
**Financial Disclosure**<br><br>Enter a financial disclosure statement that describes the sources of funding for the work included in this submission. Review the submission guidelines for detailed requirements. View published research articles from *PLOS ONE* for specific	The author(s) received no specific funding for this work.

examples.<br><br>This statement is required for submission and **will appear in the published article** if the submission is accepted. Please make sure it is accurate.<br><br>**Funded studies**<br>Enter a statement with the following details:<br>• Initials of the authors who received each award<br>• Grant numbers awarded to each author<br>• The full name of each funder<br>• URL of each funder website<br>• Did the sponsors or funders play any role in the study design, data collection and analysis, decision to publish, or preparation of the manuscript?<br><br>Did you receive funding for this work?	
**Competing Interests**<br><br>Use the instructions below to enter a competing interest statement for this submission. On behalf of all authors, disclose any competing interests that could be perceived to bias this work—acknowledging all financial support and any other relevant financial or non-financial competing interests.<br><br>This statement is required for submission and **will appear in the published article** if the submission is accepted. Please make sure it is accurate and that any funding sources listed in your Funding Information later in the submission form are also declared in your Financial Disclosure statement.<br><br>View published research articles from *PLOS ONE* for specific examples.	The authors have declared that no competing interests exist.

**NO authors have competing interests**<br><br>Enter: *The authors have declared that no competing interests exist.*<br><br>**Authors with competing interests**<br><br>Enter competing interest details beginning with this statement:<br><br>*I have read the journal's policy and the authors of this manuscript have the following competing interests: [insert competing interests here]*<br><br>* typeset	
**Ethics Statement**<br><br>Enter an ethics statement for this submission. This statement is required if the study involved:<br><br>• Human participants<br>• Human specimens or tissue<br>• Vertebrate animals or cephalopods<br>• Vertebrate embryos or tissues<br>• Field research<br><br>Write "N/A" if the submission does not require an ethics statement.<br><br>General guidance is provided below. Consult the submission guidelines for detailed instructions. **Make sure that all information entered here is included in the Methods section of the manuscript.**	N/A

**Format for specific study types**<br><br>**Human Subject Research (involving human participants and/or tissue)**<br>• Give the name of the institutional review board or ethics committee that approved the study<br>• Include the approval number and/or a statement indicating approval of this research<br>• Indicate the form of consent obtained (written/oral) or the reason that consent was not obtained (e.g. the data were analyzed anonymously)<br><br>**Animal Research (involving vertebrate animals, embryos or tissues)**<br>• Provide the name of the Institutional Animal Care and Use Committee (IACUC) or other relevant ethics board that reviewed the study protocol, and indicate whether they approved this research or granted a formal waiver of ethical approval<br>• Include an approval number if one was obtained<br>• If the study involved *non-human primates*, add *additional details* about animal welfare and steps taken to ameliorate suffering<br>• If anesthesia, euthanasia, or any kind of animal sacrifice is part of the study, include briefly which substances and/or methods were applied<br><br>**Field Research**<br><br>Include the following details if this study involves the collection of plant, animal, or other materials from a natural setting:<br>• Field permit number<br>• Name of the institution or relevant body that granted permission	
**Data Availability**<br><br>Authors are required to make all data underlying the findings described fully available, without restriction, and from the time of publication. PLOS allows rare exceptions to address legal and ethical concerns. See the PLOS Data Policy and FAQ for detailed information.	Yes - all data are fully available without restriction

A Data Availability Statement describing where the data can be found is required at submission. Your answers to this question constitute the Data Availability Statement and **will be published in the article**, if accepted.<br><br>**Important:** Stating 'data available on request from the author' is not sufficient. If your data are only available upon request, select 'No' for the first question and explain your exceptional situation in the text box.<br><br>Do the authors confirm that all data underlying the findings described in their manuscript are fully available without restriction?	
**Describe where the data may be found in full sentences. If you are copying our sample text, replace any instances of XXX with the appropriate details.**<br><br>• If the data are **held or will be held in a public repository**, include URLs, accession numbers or DOIs. If this information will only be available after acceptance, indicate this by ticking the box below. For example: *All XXX files are available from the XXX database (accession number(s) XXX, XXX.).*<br>• If the data are all contained **within the manuscript and/or Supporting Information files**, enter the following: *All relevant data are within the manuscript and its Supporting Information files.*<br>• If neither of these applies but you are able to provide **details of access elsewhere**, with or without limitations, please do so. For example:<br><br>*Data cannot be shared publicly because of [XXX]. Data are available from the XXX Institutional Data Access / Ethics Committee (contact via XXX) for researchers who meet the criteria for access to confidential data.*<br><br>*The data underlying the results presented in the study are available from (include the name of the third party*	All data used for analyses is present within either in-text tables or supplementary tables. Additionally, coding has been included that produced our discussed quantitative results.

and contact information or URL).<br>• This text is appropriate if the data are owned by a third party and authors do not have permission to share the data.<br><br>* typeset	
Additional data availability information:	Tick here if the URLs/accession numbers/DOIs will be available only after acceptance of the manuscript for publication so that we can ensure their inclusion before publication.

Powered by Editorial Manager® and ProduXion Manager® from Aries Systems Corporation

Manuscript
Click here to access/download;Manuscript;Avisaurus_ONE (07.01.24).docx

Full title: New enantiornithine diversity in the Hell Creek Formation and the functional morphology of the avisaurid tarsometatarsus

Short title: New Hell Creek enantiornithines and avisaurid tarsometatarsi functional morphology

Alexander D. Clark[1,2*], Jessie Atterholt[3], John B. Scannella[4], Thomas D. Carr[5], Nathan Carroll[6], Jingmai K. O'Connor[2]

[1]Committee on Evolutionary Biology, University of Chicago, Chicago, IL 60637 USA
[2]Negaunee Integrative Resource Center, Field Museum of Natural History, 1400 S Dusable Lake Shore Drive, Chicago, IL. 60605 USA
[3]College of Osteopathic Medicine of the Pacific, Western University of Health Sciences, Pomona, CA, USA
[4]Museum of the Rockies and Department of Earth Sciences, Montana State University, 600 W. Kagy Blvd, Bozeman, Montana, 59717, USA
[5]Department of Biology, Carthage College, Kenosha, WI 53140, USA
[6]Carter County Museum, 306 N Main St. Ekalaka, MT, 59324 USA

Corresponding author: Alexander D. Clark, adclark@uchicago.edu

Abstract

Enantiornithines were the most diverse group of birds during the Cretaceous, comprising over half of all known species from this period. The fossil record and subsequently our knowledge of this clade is heavily skewed by the wealth of material from Lower Cretaceous deposits in China. In contrast, specimens from Upper Cretaceous deposits are rare and typically fragmentary, yet critical for understanding the extinction of this clade across the K-Pg boundary. The most complete North American Late Cretaceous enantiornithine is *Mirarce eatoni*, a member of the diverse clade Avisauridae. Except for *Mirarce*, avisaurids are known only from isolated hindlimb elements from North and South America. Here we describe three new enantiornithines from the Maastrichtian Hell Creek Formation, two of which represent new avisaurid taxa. These materials represent a substantial increase in the known diversity of Enantiornithes in the latest Cretaceous. Re-examination of material referred to Avisauridae through phylogenetic analysis provides strong support for a more exclusive Avisauridae consisting of six taxa. Exploration of the functional morphology of the avisaurid tarsometatarsus indicates strong grip and raptorial attributes. The lower aspect ratio of the tarsometatarsus facilitates a more biomechanically efficient lever system for lifting proportionally heavier objects. In addition, the proportional size and distal position of the *m. tibialis cranialis* tubercle of the tarsometatarsus is similar to the morphology seen in extant birds of prey. Together with the deeply-grooved metatarsal trochlea facilitating robust and likely powerful pedal digits, morphologies of the hindlimb suggest avisaurids as Late Cretaceous birds of prey.

Keywords: Enantiornithes, paleobiology, Hell Creek Formation, paleoecology, Mesozoic birds

Introduction

Our understanding of Mesozoic avian diversity and evolution is limited by available material, increasing with each new described taxon. Several Lower Cretaceous Lagerstätten have yielded abundant fossils which provide insights to the diversity, ecology, and taxonomy of Mesozoic birds relatively early in their evolution (Zhou and Zhang, 2006). However, information from Upper Cretaceous deposits is far more limited, with the only possible Lagerstätten being a newly emerging site dubbed "William's Quarry" (Wu et al., 2021). Late Cretaceous fossil birds are critical for understanding one of the most important aspects of avian evolution: the selectivity of the end Cretaceous Mass Extinction in which only neornithines survived.

The most speciose clade of Mesozoic birds, constituting over half of all described species, are the enantiornithines (Chiappe and Walker, 2002; O'Connor et al., 2011; Walker, 1981). Members of this clade exhibit morphological diversity and are found on every continent except for Antarctica and continental Africa (Chiappe and Walker, 2002; O'Connor and Chiappe, 2011; Walker, 1981, Zhou et al., 2008). The vast majority of known specimens and described taxa come from Hauterivian-Aptian Lower Cretaceous deposits in Spain and China. As a result, our knowledge of this clade is heavily biased to a narrow time interval of approximately 10 million years (Buscalioni and Poyato-ariza, 2016; Zhou and Zhang, 2006).

Enantiornithine fossils from the Late Cretaceous are far more limited but critical for understanding the extinction of this once successful lineage. The only widely recognized clade from Upper Cretaceous deposits is the Avisauridae, known exclusively from North and South American deposits 86-66 Ma. Six genera have been named in the literature: *Avisaurus* (Brett-Surman & Paul, 1985), *Soroavisaurus* (Chiappe, 1993), *Neuquenornis* (Chiappe & Calvo, 1994), *Intiornis* (Novas et al., 2010), *Mirarce* (Atterholt et al., 2018), and *Gettyia* (formerly *A. gloria)* (Atterholt et al., 2018; Varrichio and Chiappe, 1995). Members of this group are mostly described from fragmentary remains, with the tarsometatarsus being the only commonly recovered element and thus critical for recognizing members of this clade. Here, we reevaluate the taxa assigned to the Avisauridae using cladistic analysis and in doing so, remove *Intiornis* and *Neuquenornis* from this group.

Previously, three synapomorphies were hypothesized to unite Avisauridae: a strongly convex dorsal margin of metatarsal III; the plantar projection of the medial condyle of metatarsal III; and a J-shaped laterally compressed metatarsal I (Chiappe and Calvo, 1994). One cladistic analysis suggested this clade also included *Enantiophoenix, Halimornis* and *Concornis* (Cau and Arduini, 2008). However, support for this hypothesis is weak. The tarsometatarsus is unknown in *Halimornis,* and thus referral to the Avisauridae is ambiguous. Similarly, the tarsometatarsus of *Enantiophoenix* is poorly preserved, making the assignment to the Avisauridae based on the convexity of the dorsal surface equivocal. Convexity of the dorsal margin of metatarsal III appears to be more widespread than previously thought, being present in non-avisaurid enantiornithines (e.g., *Imparavis, Feitianius*) (O'Connor et al., 2016; Wang et al., 2024). Additionally, as in *Enantiophoenix*, the lack of convexity in other enantiornithines may be taphonomic.

Assignment to the Avisauridae for *Concornis* was also based on the convexity of the dorsal margin of metatarsal III, and stoutness of the first phalanx of pedal digit I (Cau and Arduini, 2008). In this study, *Halimornis* and *Concornis* were regarded as distinct lineages within the Avisauridae, although with little morphological support (Cau and Arduini, 2008).

The taxon from which this family derives its name, *Avisaurus archibaldi*, is a large enantiornithine, with a tarsometatarsal length (~ 75 mm) comparable to large extant larids (e.g., *Larus marinus*, *L. glaucescens*, 900 – 1800 g) and accipitrids (e.g., *Buteo jamaicensis*, *Accipiter gentilis*, ~1400 g) (Brett-Surman and Paul, 1985). This is in stark contrast to nearly all Early Cretaceous enantiornithines, whose average tarsometatarsal length varies between extant emberizids (old-world sparrows, ~20 g) and turdids (thrushes, ~80 g), tending not to exceed 25 mm in length (Chiappe and Walker, 2002) (Table S1). Avisaurids not only add to the morphological diversity of enantiornithines, but also contribute considerably to our understanding of the apparent trend of increasing body size diversity in the Late Cretaceous (Atterholt et al., 2018; Brett-Surman and Paul, 1985).

Here we describe three new enantiornithine specimens, all of which come from the Hell Creek Formation (Latest Cretaceous 68-66 Ma) (Fowler, 2020). One of these new taxa is considered a new

species within the genus *Avisaurus* and another is assigned to the *Avisaurus* genus. Previously, *A. archibaldi* was the only avisaurid and enantiornithine described from these deposits (Brett-Surman and Paul, 1985). Like previously described North American avisaurids except for *Mirarce*, the new taxa are all represented by isolated tarsometatarsi. Although the material is limited, they possess morphological differences from previously known Late Cretaceous enantiornithines. Here, we revise morphological and phylogenetic diagnoses for the Avisauridae and discuss the significance of these new specimens with regards to their phylogenetic position, implications for size diversity among enantiornithines, and the functional morphology of the avisaurid tarsometatarsus.

INSTITUTIONAL ABBREVIATIONS – DDM, Dinosaur Discovery Museum Kenosha, Wisconsin, United States; MOR, Museum of the Rockies Bozeman, Montana, United States; CCM, Carter County Museum Ekalaka, Montana, United States.

Methods

Specimens were photographed under normal lighting conditions with a Canon 70D dslr camera with a Canon EF 100mm f/2.8 macro lens. Photos of each specimen were assessed in Adobe Photoshop version 24.3.0 and if needed, had aspects of contrast, and color balance edited. Anatomical nomenclature primarily follows Baumel and Witmer (1993) using the English equivalents of the Latin terminology. All measurements used for description and comparisons were recorded in-person using digital calipers (OriginCal1P54). Additional measurements were recorded with ImageJ (Schneider et al., 2012).

Systematic Paleontology

Aves Linnaeus, 1758

Pygostylia Chiappe, 2002

Ornithothoraces Chiappe, 1995

Enantiornithes Walker, 1981

Avisauridae Brett-Surman and Paul, 1985

Diagnosis: Enantiornithines characterized by the combination of a robust tarsometatarsus (mediolateral width at the midpoint at least 20% that of the total proximodistal length); a mediolaterally broad trochlea of metatarsal II relative to III; the presence of a hypertrophied tubercle for the attachment of the *m. tibialis cranialis* on metatarsal II; the *m. tibialis cranialis* tubercle located at least 20% down the length of the tarsometatarsus; and a well-developed plantar projection of the medial margin of the trochlea of metatarsal III (modified from Atterholt et al., 2018).

Included genera: *Avisaurus*– UCMP 117600, Hell Creek Formation, Late Cretaceous, Maatstrichtian, Garfield County, Montana (Brett-Surman and Paul, 1985); *Soroavisaurus*– PVL 4690, Lecho Formation, Late Cretaceous, Maastrichtian, Salta Province, Argentina (Chiappe, 1993); *Mirarce*– UCMP 139500, Kaiparowits Formation, Late Cretaceous, Campanian, Grand Staircase-Escalante National Monument, Utah (Atterholt et al., 2018); *Gettyia*– MOR 553E/1.19.91.64, Two Medicine Formation Late Cretaceous, Campanian, Glacier County, Montana (Atterholt et al., 2018).

Avisaurus Brett-Surman and Paul, 1985

Diagnosis: Avisaurid enantiornithines characterized by the following combination of characters: distal extent of metatarsal II approximately equal to that of metatarsal IV; the proximoplantar surface excavated by a pair of small fossae located where the medial and lateral margins of metatarsal III meet the plantar labrum, hereafter referred to as the proximoplantar fossae (nomen novum) (modified from Chiappe, 1993).

Included species: *Avisaurus archibaldi* – UCMP 117600, Late Cretaceous, Maatstrichtian, Garfield County, Montana (Brett-Surman and Paul, 1985); *Avisaurus darwini* – DDM 1577.730, Hell Creek Formation, Late Cretaceous, Maastrichtian, Carter County, Montana (this publication).

Avisaurus darwini sp. nov. (Fig. 1, Table 1)

Holotype: DDM 1577.730 is a complete right tarsometatarsus collected in July of 2022

Etymology: The specific name "*darwini*", is in honor of Charles Darwin, whose momentous research and publications helped define the field of evolutionary biology. *Avisaurus darwini*, Darwin's bird lizard.

Type horizon and locality: Hell Creek Formation, Latest Cretaceous, Maastrichtian (approximately 66 Ma). DDM 1577.730 was collected in Carter County, Montana at a locality under the auspices of the Bureau of Land management. The specimen was collected from the Madison Shark Spine locality (DDM-18-234,1N 55E 11) within a grey sand multitaxic microvertebrate site.

FIGURE 1. The left tarsometatarsus of *Avisaurus darwini* (DDM 1577.730) in A) dorsal, B) plantar, C) right lateral, D) left lateral, E) proximal, and F) distal aspects. Abbreviations: dlp, distal lateral projection; dmp, distal medial projection; dvf, distal vascular foramen; fo, fossa (possible collateral ligament fossa); is, intermetatarsalian space; lc, lateral cotyle; lf, laterally-projecting flange; mc, medial cotyle; mtII, metatarsal II; mtIII, metatarsal III; mtIV, metatarsal IV; pf, proximoplantar fossae; pl, proximal labrum; pp, plantar projection; tct, *m. tibialis cranialis* tubercle. Scale bar 10mm.

	Avisaurus darwini	***MOR 3070***	***Magnusavis ekalakaensis***
**Proximal Surface**			
Width	20.24	18.56	-

Height	11.65	11.15	-
**Cranial Aspect**			
MT II Length	62.93	*58.68	-
MT II Width (midpoint)	4.91	-	3.12
MTIII Length	65.38	-	-
MT III Width (midpoint)	6	-	4.8
MT IV Length	60.4	-	-
MT IV Width (midpoint)	4.98	-	-
**Distal Aspect**			
MT II Trochlea Width	8.07	-	7.38
MT II Trochlea Height	5.22	-	5.78
MT III Trochlea Width	8.18	-	8.27
MT III Trochlea Height	6.62	-	5.87
MT IV Trochlea Width	9.48	-	-
MT IV Trochlea Height	4.64	-	-
**Aspect ratio of Tmt**	1:4	*1:4	-

TABLE 1. Measurements (in mm) of each newly described specimen. For MOR 3070, asterisks represent approximations. Total length of metatarsal II was estimated by scaling MOR 3070 to *A. darwini*, who is nearly the same size.

Diagnosis –A large accipitrid-sized bird identified as an avisaurid based on the strong plantar projection of the medial condyle of the trochlea of metatarsal III; a robust tarsometatarsus (mediolateral width at the midpoint greater than 20% that of the total proximodistal length); a mediolaterally broad trochlea of metatarsal II relative to III; the presence of a hypertrophied *m. tibialis cranialis* tubercle on metatarsal II located ~ 30% down the tarsometatarsus. DDM 1577.730 is referable to the genus *Avisaurus* based on the following features: the distal extent of metatarsal II approximately equal to that of metatarsal IV; the presence of proximoplantar fossae; a metatarsal II exhibiting a laterally-projecting flange of bone from the plantar margin. DDM 1577.730 can be differentiated from other avisaurids based on the following unique combination of features: a proximally located intermetatarsal space between metatarsals III and IV which is proximodistally proportionally shorter than that of *Soroavisaurus*; metatarsal II's laterally-

projecting flange of bone closes the intertrochlear incisure, contacting the distomedial margin of the metatarsal III trochlea (this feature is more pronounced than that in the lateroplantarly-projecting flange in *A. archibaldi*); both medial and lateral margins of the trochlea of metatarsal III terminate distally at the same level, disparate from *Soroavisaurus* (lateral margin extending distally) and *A. archibaldi* (medial margin extending distally).

Avisaurus sp. (Fig. 2, Table 1)

Material: MOR 3070 is a partial right tarsometatarsus preserving most of metatarsal II (distal portion disarticulated), < 50% of metatarsal III, and the proximal portion of metatarsal IV. Some additional disarticulated fragments are probably referable to the specimen but lack diagnostic morphologies.

Locality: Hell Creek Formation, Latest Cretaceous, Maastrichtian (approximately 68 - 66 Ma). MOR locality no. HC-720 “Daigo’s Site”. MOR 3070 was discovered by Daigo Yamamura in Makoshika State Park, Glendive, Montana and collected by a field crew led by Frankie Jackson of Montana State University (Yamamura, 2013).

FIGURE 2. The left partial tarsometatarsus of *Avisaurus* sp. MOR3070 in A) dorsal, B) plantar, and C) proximal aspects. The distal portion of metatarsal II is placed in the approximate place if it was articulated. Abbreviations: dr, dorsal ridge: lc, lateral cotyle; lf, laterally-projecting flange; mc, medial cotyle; mtII, metatarsal II; mtIII, metatarsal III; mtIV, metatarsal IV; pf, proximoplantar fossae; pl, proximal labrum; tct, *m. tibialis cranialis* tubercle. Scale bar 10mm.

Comment – A large accipitrid-sized enantiornithine with plantar-projecting medial and lateral plantar crests; dorsal face of metatarsal II is mediolaterally wider than metatarsal III proximally; the lateral cotyle

elevated slightly proximally relative to the medial cotyle. MOR 3070 is referable to the genus *Avisaurus* based on the two proximoplantar fossae in plantar aspect, and a metatarsal II trochlea exhibiting a lateroplantarly-projecting flange of bone from the plantar margin. MOR 3070 differs from other closely related enantiornithines (avisaurids) based on the following unique combination of features: lateral to medial tapering of the proximal labrum's dorsal margin; a dorsally-projecting *m. tibialis cranialis* tubercle rather than dorsomedially projection as in *A. archibaldi* and *A. darwini*; metatarsal II mediolaterally greater than that of III in dorsal aspect along its proximal preserved portion; a well-developed dorsally-projecting ridge on the dorsal face of metatarsal III beginning prominently below the labrum shelf and continuing distally down the dorsal surface of the remaining portions of metatarsal III (forming a triangular cross section in plantar aspect); plantar margin of the trochlea of metatarsal II with a lateroplantarly-projecting flange, similar to *A. archibaldi* but disparate from the lateral-projecting morphology in *A. darwini*.

Aves Linnaeus, 1758

Pygostylia Chiappe, 2002

Ornithothoraces Chiappe, 1995

Enantiornithes Walker, 1981

Magnusavis ekalakaenis gen. et sp. nov. (Fig. 3, Table 1)

Holotype: CCM V2019.5.1 is a partial right tarsometatarsus, missing the proximal portions of metatarsals II and II, and all of metatarsal IV. A single phalanx is preserved, though digit assignment is uncertain.

Etymology: In Latin, "*Magnus*" meaning big, and "*avis*" meaning bird, and "*ekalakaensis*" in honor of the town of Ekalaka, Montana, close to where this specimen was discovered. *Magnusavis ekalakaenis*, Ekalaka's big bird.

Locality: Hell Creek Formation, Late Cretaceous, Maastrichtian (approximately 66 Ma). The specimen was collected in Carter County, Montana at a locality under the auspices of the Bureau of Land management (Permit MTM 108846).

FIGURE 3. The partial left tarsometatarsus of *Magnusavis ekalakaensis* (CCM V2019.5.1) in A) dorsal, B) plantar, and C) distal aspects, D) and the single preserved pedal phalanx in dorsal and right lateral aspects. Abbreviations; mtII, metatarsal II; mtIII, metatarsal III; pp, proximal projection; psf, plantar supratrochanter fossa; tct, *m. tibialis cranialis* tubercle. Scale bar 10mm.

Diagnosis – A larid-sized enantiornithine differentiated from other taxa, including the closely-related avisaurids, based on the following unique combination of features: distal expansion of metatarsals II and III (all avisaurids retain nearly equal medial and lateral margins of the metatarsals down their lengths); a medial and lateral-rimmed (i.e., well-excavated) plantar supratrochanter fossa on metatarsal III; and an articular face on metatarsal III (for metatarsal IV) terminating distal to that in *A. archibaldi*.

Phylogenetic Analysis

These three new specimens were added to a modified version of the character matrix by Atterholt et al. (2018). We added three new characters to assess the relative robustness of the tarsometatarsus [Character 253], presence of the proximoplantar fossae bordering metatarsal III [Character 254], and the presence of the intermetatarsal space between metatarsals III and IV proximal to the midpoint of the tarsometatarsus [Character 255]. The final matrix, consisting of 45 taxa with scores across 255 characters, was analyzed in TNT (Goloboff et al., 2008). For scoring, see Supplementary Information. Neornithes were represented by *Anas* and *Gallus*, and Dromaeosauridae was used as the outgroup. We conducted a heuristic search retaining the single shortest tree out of every 1000 trees followed by a second round of

tree-bisection reconnection (TBR). The first round of TBR produced 36 trees with a length of 803 steps; the second round of TBR produced 4207 trees of the same length.

Results

Morphological descriptions

DDM 1577.730 – Holotype of *Avisaurus darwini*

The metatarsals are all fused proximally to each other and the distal tarsals. The proximal articular surface is oval in shape with the lateral cotyle slightly elevated and larger relative to the medial cotyle as in *A. archibaldi*. It is greater than the cross-section of metatarsals II-IV such that it forms a well-developed labrum that overhangs the metatarsal shafts in both dorsal and plantar aspects as in *Mirarce* and *A. archibaldi* (Atterholt et al., 2018; Brett-Surman & Paul, 1985). Like *A. archibaldi*, this is more pronounced in plantar aspect. In plantar view, just below the labrum, the proximal-most lateral and medial portions of the metatarsal III shaft are each excavated by a small fossa, referred to as the proximoplantar fossae (Fig. 1, B). These fossae are also present, although less pronounced, in *A. archibaldi* and MOR 3070, but absent in other avisaurids.

In dorsal aspect, the metatarsals do not distally expand mediolaterally, similar to *A. archibaldi*, *Gettyia*, and *Mirarce* (Atterholt et al., 2018; Brett-Surman & Paul, 1985) (Fig. 4). As in *Soroavisaurus*, there is a large intermetatarsal space between metatarsals III and IV (Fig. 1, A&B), originally referred to as a “fenestra” Chiappe (1993). This structure may be analogous to the lateral proximal vascular foramen in extant birds which allows for anastomosis between the dorsal and plantar surfaces (Chiappe, 1993; Jadwiszczak, 2015; Schwehn et al., 2024). The mediolateral width of the space measures approximately 45% of the midpoint of metatarsal IV. In *Soroavisaurus*, this space is proximodistally longer and begins

more proximally, extending from nearly 5% to 36% the length of the tarsometatarsus (Chiappe, 1993). In *Intiornis* the space extends from 12% to 30% the length of the tarsometatarsus (Novas et al., 2010).

Metatarsal III is the longest, as in all avisaurids, and most enantiornithines (Atterholt et al., 2018; Chiappe and Walker, 2002). The proximodistal length of metatarsals II and IV measures 96% and 92% that of metatarsal III respectively. Metatarsal III also exhibits the greatest mediolateral width measured at the midpoint. Metatarsals II and IV measure 82% and 83% the width of metatarsal III, respectively (Table 1). Like other avisaurids (but also some non-avisaurid enantiornithines), the dorsal surface of metatarsal III is strongly convex forming a weak ridge for most of its length. The shaft of metatarsal II is similarly convex, unlike other avisaurids where this face is comparatively flatter. The distal portion of metatarsal II and its trochlea deflects medially as in *A. archibaldi*, *Gettyia*, and *Mirarce* (Atterholt et al., 2018; Brett-Surman & Paul, 1985). The shaft of metatarsal IV is cranioplantarly compressed similar to *A. archibaldi* and *Gettyia*. The distal portion of the metatarsal gently curves laterally just proximal to the mediolaterally expanded trochlea more so than *Soroavisaurus* and *Mirarce* (Atterholt et al., 2018; Brett-Surman & Paul, 1985; Chiappe, 1993).

FIGURE 4. Tarsometatarsus size variation among the Avisauridae and close relatives. A) *Avisaurus darwini*, B) MOR3070, C) *Avisaurus archibaldi*, D) *Gettyia*, E) *Soroavisaurus*, F) *Mirarce*, G) *Intiornis*, H) *Neuquenornis*. All to scale. Scale bar 10 mm.

On the craniomedial surface of metatarsal II there is a large and rugose tubercle for the attachment of the *m. tibialis cranialis* (Fig. 1, A&C). This structure is elongate, beginning 29% of the way down metatarsal II and extending for 15% of its total length, similar to *A. archibaldi* both in size and position (Brett-Surman & Paul, 1985). Distally, there is a weakly-developed intertrochlear incisure between metatarsals II and III. The space extends for approximately 11% that of the total length of metatarsal III. The distal vascular foramen between trochlea III and IV begins 78% down the length of

metatarsal III and terminates at approximately 86%. The mediolateral width of this space measures approximately 34% that of the midpoint width of metatarsal IV.

The laterally-projecting flange of the lateral trochlea of metatarsal II expands such that the trochlea along the plantar margin is wider than that of metatarsal III, unlike *Soroavisaurus*. In *A. archibaldi*, this flange is proportionally smaller, projects lateroplantarly, and does not fully close the gap between the trochlea of metatarsal II and III(Brett-Surman & Paul, 1985). In *Mirarce*, this feature is also present, but is located more distally on metatarsal II and expands distolaterally (Atterholt et al., 2018). In *A. darwini*, this distal laterally-projecting flange contacts that distomedial margin of metatarsal III, closing the gap between the trochlea of metatarsals II and III. This closure creates a condition analogous to the distal vascular foramen between the trochlea of metatarsal III and IV (Atterholt et al., 2018).

The medial and lateral condyles of the metatarsal III trochlea terminate approximately at the same level, whereas the medial margin extends farther distally in *A. archibaldi* (Brett-Surman & Paul, 1985). As in *A. archibaldi* and *Mirarce*, the medial condyle of the trochlea of metatarsal III exhibits greater plantar projection than the lateral condyle (Atterholt et al., 2018; Brett-Surman & Paul, 1985). The trochlea of both metatarsals II and III are ginglymous, and exhibit well-developed, excavated, intercondylar grooves. These well-developed grooves are also present in *A. archibaldi*, *Mirarce*, *Gettyia*, and *Soroavisaurus* (Atterholt et al., 2018; Brett-Surman & Paul, 1985; Chiappe, 1993). The trochlea of metatarsal IV has a reduced medial condyle such that it appears as a single, plantomedially excavated condyle, similar to other enantiornithines (e.g., *A. archibaldi*, *Avimaia*, *Imparavis*, *Mirarce)* (Atterholt et al., 2018; Bailleul et al., 2019; Brett-Surman & Paul, 1985; Wang et al., 2024). In distal view, the trochlea of metatarsal IV is C-shaped (medially concave), and projects farther plantarly than even the medial condyle of the metatarsal III trochlea, as in other avisaurids (e.g., *Mirarce, A. archibaldi*) (Atterholt et al., 2018; Brett-Surman & Paul, 1985).

Specimen MOR 3070 - *Avisaurus* sp.

The metatarsals are all fused proximally to each other and the distal tarsals (Fig. 2). Similar to other *Avisaurus*, the proximal articular surface is oval with the lateral cotyle slightly elevated relative to the medial cotyle. The proximal labrum is well-developed as in *A. archibaldi*, *A. darwini*, and *Mirarce* (Atterholt et al., 2018; Brett-Surman & Paul, 1985). However, unlike *A. darwini*, and *Mirarce*, MOR 3070 exhibits greater lateral to medial tapering of the labrum thickness along the dorsal margin (Fig. 2, A; Atterholt et al., 2018). In plantar aspect, the labrum is dorsoventrally thickest at its midpoint (i.e., above the midline of metatarsal III). Characteristic of the genus *Avisaurus*, two proximoplantar fossae bordering metatarsal III are present. These fossae are not as deep as in *A. darwini*.

Though incomplete, MOR 3070 would have had a low aspect ratio of the tarsometatarsus (~ 1:4) like other avisaurids (e.g., *Mirarce*, *Gettyia*, and *Avisaurus*). Proximally, metatarsal II is the mediolaterally widest metatarsal, with the width of metatarsal III measuring approximately 87% that of the metatarsal II. This in contrast to *Mirarce*, *A. archibaldi*, and *A. darwini*, where even proximally the width of metatarsal II is less than that of III (Atterholt et al., 2018). Similar to members of *Avisaurus*, MOR 3070 exhibits a well-developed *m. tibialis cranialis* tubercle which if scaled to *Avisaurus* would place it approximately 30% down the length of metatarsal II. In contrast to other avisaurids, the *m. tibialis cranialis* tubercle of MOR 3070 projects more dorsally as opposed to dorsomedially. The disarticulated distal portion of metatarsal II exhibits similarities to *A. archibaldi* in that it exhibits a lateroplantarly-projecting flange (Brett-Surman & Paul, 1985). This is disparate from the laterally-projecting morphology exhibited in *A. darwini*. The relationship of this flange with the trochlea metatarsal III is unknown, but similar to *A. archibaldi*, it likely did not contact metatarsal III (this flange contacts the trochlea of metatarsal III in *A. darwini*). Beginning prominently below the labrum shelf, a well-developed dorsally-projecting ridge extends distally down the remaining portion of the dorsal surface of metatarsal III (forming a triangular cross section in plantar aspect). This feature is absent in *A. darwini*, and weakly developed in *A. archibaldi*.

CCM V2019.5.1 – Holotype of *Magnusavis ekalakaenis*

The holotype of *M. ekalakaenis* consists of partial metatarsals II and III and a single phalanx (Fig. 3). Unlike avisaurids, both preserved metatarsals exhibit subtle distal expansion. The proximal-most mediolateral width of metatarsal III measures 76% that of the distal-most portion (proximal to the trochlea) (*A. darwini*, 100%, *A. archibaldi*, 96%). Metatarsal III is both proximodistally longer and mediolaterally wider than metatarsal II along its length, typical of most enantiornithines (Chiappe and Walker, 2002).

The shaft of metatarsal II is rounded and smooth, in contrast to the well-developed peaked ridge in MOR 3070 and *A. darwini*. The distomedial deflection of metatarsal II is weakly-developed compared to the condition seen in avisaurids, exhibiting a nearly closed intertrochlear incisure between metatarsal II and III. Though not as pronounced or as large as the condition seen in avisaurids, the *m. tibialis cranialis* tubercle is well developed when compared to most enantiornithines (Atterholt et al., 2018; Brett-Surman & Paul, 1985). Similar to metatarsal II, the dorsal surface of metatarsal III is only weakly convex, in contrast to the strongly convex morphology in avisaurids (e.g., *Mirarce, A. archibaldi*, MOR 3070, and, *A. darwini*).

The condyles of the metatarsal III trochlea terminate at the same level distally. Like avisaurids, the intercondylar grooves are well-developed in both metatarsals II and III, particularly in III. The medial condyle of the metatarsal III trochlea exhibits greater dorsal and plantar projection relative to the lateral condyle. The plantar supratrochanter fossa is also well developed, and clearly demarcated lateral and medially. The mediolateral width of this depression measures approximately 75% the total width of the trochlea of metatarsal III. The lateral fossa of metatarsals II and III are well-developed.

Though digit assignment of the phalanx is uncertain, it resembles the first phalanx of pedal digit II in *Mirarce*, albeit much more dorsoventrally deep (lateral aspect ratio *Magnusavis* 1:3, *Mirarce* 1:6). The aspect ratio of the phalanx is approximately 1:3, in contrast to that seen in Mirarce (~ 1:5).

Phylogenetic Analysis Results

Both the Nelson strict consensus tree and the 50% majority rule tree place all three new taxa within enantiornithines, with *A. darwini* and MOR 3070 (*Avisaurus* sp.) resolved as part of the Avisauridae clade in the 50% majority rule tree only (Fig. 5).

FIGURE 5. Phylogenetic placement of new Hell Creek enantiornithines based on cladistic analysis. A) The strict consensus and B) majority tree. In the majority tree, the newly diagnosed Avisauridae family in comprised of six taxa, two of which are described in this publication. C) A 1.8 m tall human to scale with *A. darwini* (maroon), the largest known Early Cretaceous enantiornithine *Pengornis* (black), a medium-sized early enantiornithine, *Imparavis* (white), and finally, a minuscule enantiornithine, *Elektorornis* (smaller inset black). The extant *Buteo jamaicensis* (Red-tailed Hawk) is shown in grey.

Within the Avisauridae, *A. archibaldi* and *A. darwini* form a more exclusive clade supported by a metatarsal II that is approximately equal in distal extent to metatarsal IV [Character 236 1 → 0]. *Mirarce* and *Gettyia* form a clade supported by the absence of a plantarly excavated tarsometatarsus [Character 230 1 → 0], a *m. tibialis cranialis* tubercle located near or distal to the midpoint [Character 247 1 → 2], and the absence of proximoplantar fossae [Character 254 1 → 0]. These two clades form a polytomy with MOR 3070 and *Soroavisaurus*. The Avisauridae clade formed by these six OTU (operational taxonomic units) is supported by a tarsometatarsus whose midpoint width measures at least 20% that of its length (i.e., low aspect ratio) [Character 253 1 → 0], and a metatarsal II trochlea that is broader than that of metatarsal III [Character 238 0 → 1].

Magnusavis, *Neuquenornis*, and *Intiornis* are part of a larger polytomy outside of the Avisauridae along with *Mystiornis*, *Halimornis*, *Shenqiornis*, and *Elsornis*. With the removal of two previously identified avisaurids (*Neuquenornis*, and *Intiornis*), this family is now primarily defined not by the convex dorsal surface of metatarsal III or plantar excavation of the tarsometatarsus, characters that are both widespread in enantiornithines (e.g., *Imparavis*, *Elektorornis*, *Qiliania*) (Ji et al., 2011; Xing et al.,

2019; Wang et al., 2024), but instead by a proportionately robust tarsometatarsus (mediolateral width at the midpoint at least 20% that of the total proximodistal length). The removal of these two taxa differs from previous phylogenetic results which place them alongside avisaurids based on a plantar projection of the medial trochlea of metatarsal III (Atterholt et al., 2018). These results support interpretations that MOR 3070 is an avisaurid and likely a member of the genus *Avisaurus* (e.g., robust morphology, two proximoplantar fossae), and one that differs from other avisaurids; however, in light of the fragmentary remains available at present, we refrain from naming this specimen as a new taxon.

Discussion

Size Diversity in Late Cretaceous Enantiornithines

Here we describe three new large birds from the Maastrichtian Hell Creek Formation in Montana, all of which are placed in the clade Enantiornithes (Fig. 5). These three new enantiornithines not only increase the known taxonomic diversity of the Hell Creek avifauna, but they also add to existing evidence that members of this speciose clade achieved proportionally larger body sizes near the end of the Cretaceous.

Most enantiornithines described from Lower Cretaceous deposits, notably the Hauterivian to Aptian Lagerstätten in Spain and China (~130 – 120 Ma), and Upper Cretaceous amber specimens from Myanmar (~ 99 - 100 Ma), are on average similar in size to extant sparrows (families Passeridae, Emberizidae) (~10 – 70 grams) and thrushes (family Turdidae) (~30 – 80 grams) (Chiappe and Walker, 2002; O'Connor et al., 2009; Wang et al., 2024; Zhou, 2002). Currently, the smallest Upper Cretaceous enantiornithines are Burmese amber specimens (*Elektorornis*, approximately the size of 3 g extant trochilids) and the largest is the holotype of *Pengornis houi* [similar in size to large psittaciforms (600-750 grams) (Zhou et al., 2008)].

The tarsometatarsus scales with size in extant birds (Field et al., 2013) (Fig. S1). Extant birds with similar tarsometatarsal lengths to avisaurids are found among modern gulls (family Laridae) and hawks (family Acciptridae) whose masses average approximately 1500 grams (Billerman et al., 2022; Zeffer and Norberg, 2003). Field et al. (2013) found the greatest predictive power (highest R^2 scores) in determining mass of birds was the maximum diameter of the glenoid facet of the coracoid (maximum diameter of the coracoid’s humeral articulation facet – HAF) and the length of the humerus, both which can be measured in the avisaurid *Mirarce* and suggests a body mass of 540 g in this taxon (range 432 – 808 g) (Atterholt et al., 2018). To estimate the mass of larger avisaurids known only from tarsometatarsi (*A. archibaldi* and *A. darwini*), the diameter of the coracoid glenoid and the length of the humerus were approximated by scaling them to *Mirarce* based on the tarsometatarsus, which measures 155% and 140% that of *Mirarce* in *A. archibaldi* and *A. darwini*, respectively. By averaging the masses of the nearest ten extant species to these measurements, the resulting estimated body mass for *A. archibaldi* equals ~ 1700 g (range 1488 – 2283 g), and ~1200 g for *A. darwini* (range 914 – 1712 g) (Fig. S2, Table S2).

The description of two new large-bodied avisaurids (*A. darwini*, *Avisaurus sp.*), and another proportionally large enantiornithine (*Magnusavis*), provides additional evidence in support of an observed trend in enantiornithines towards greater body size diversity over time (Atterholt et al., 2018; O’Connor, 2022) (Fig. 6). This increase in body size disparity towards the end Cretaceous is also observed in the Ornithuromorpha, the clade that includes Neornithes (Longrich et al., 2011). It has been suggested that larger body size among ornithuromorphs may have been a key contributing factor in surviving the end Cretaceous mass-extinction (Longrich et al., 2011; Torres et al., 2021). Although depositional environments yielding bird fossils in Upper Cretaceous North American deposits are biased toward the preservation of larger organisms, this does not explain the apparent absence of larger enantiornithines in Lower Cretaceous Lagerstätten like the Jehol, which records larger birds like *Jeholornis* and *Sapeornis* but only small enantiornithines (Hu et al., 2023; Zhou and Zhang, 2003). Therefore, the signal indicating increased size diversity in the Late Cretaceous is likely a genuine one. Fragmentary smaller-bodied

enantiornithines have been recovered from the Late Cretaceous of North America, both in the Hell Creek Formation and the William's quarry site in Brazil (Longrich et al., 2011; Wu et al., 2021).

FIGURE 6. Regression analyses of A) tarsometatarsus length against time, and B) proportional tarsometatarsus mediolateral width against time. Both result in significant ($x < 0.01$) positive associations with an increase in body size over time. Avisaurids are colored red and all other enantiornithine are colored black.

In enantiornithines, both an increase in tarsometatarsus length and proportional mediolateral width (at the midpoint) are significantly positively associated with time ($p < 0.01$) (Fig. 6). Though well-preserved enantiornithine tarsometatarsal material from 119 – 80 Ma is limited, represented by two specimens from Myanmar preserved in amber (e.g., DIP V 19354, *Elektorornis*) (Xing et al., 2019, 2020), before 119 Ma the average tarsometatarsus length was ~19 mm and the proportional mediolateral thickness was 12% (~2.28 mm) (aspect ratio of ~ 1:8). In contrast, these averages are 43 mm and 21% (aspect ratio 1:4 - 5) after ~ 80 Ma (Fig. 6). This pattern is likely exaggerated by the poor Upper Cretaceous fossil record and the fact no postcranial material has yet been described from William's quarry, although it is reportedly present in abundance (Wu et al., 2021).

Functional Morphology of the Avisaurid Tarsometatarsus

The Avisauridae are known almost entirely from tarsometatarsi. Members of the Avisauridae exhibit a combination of morphologies of the tarsometatarsus that are distinct from other enantiornithines and suggest raptorial behaviors including: the aspect ratio of the tarsometatarsus; the morphology and location of the *m. tibialis cranialis* tubercle; and the well-developed grooves of the trochleae. Specifically, these characteristics in avisaurids show striking similarities to strigids (owls, order Strigiformes) and accipitrids (hawks, eagles, order Accipitriformes). However, the differences between

avisaurids and these extant clades indicates that neither provides a direct analogy for the ecological role occupied by avisaurids in the latest Cretaceous. The unique union of morphologies observed in avisaurids demonstrates that they likely occupied a predatorial ecological niche unrepresented in extant forms.

Aspect Ratio of the Tarsometatarsus

The morphology and topography of the tarsometatarsus greatly varies among extant birds, and can signal certain ecological behaviors such as wading, cursoriality, perching, prey grasping abilities, and pedal digit to substrate interaction (De Mendoza and Gómez, 2022; Grant, 1971; Madan et al., 2017; Tronrud, 2022; Volkov, 2004; Zeffer and Norberg, 2003). When assessing the length and width of the tarsometatarsus of Mesozoic taxa, avisaurids overlap with a range of terrestrial nonvolant theropods (Fig. 7, Table S4). Thus, it is not surprising avisaurids were originally interpreted as non-avian, or as having reduced flight capabilities (Brett-Surman and Paul, 1985). However, when modern birds are added to the dataset, avisaurids cluster with larger birds like pelicans, owls, and hornbills weakening support for using the aspect ratio of the tarsometatarsus alone to predict volancy (Fig. 7).

FIGURE 7. Assessing aspect ratio of A) extinct avian and non-avian theropods, and then with the addition of B) extant volant birds. The grey dots represent extinct volant theropod genera. The red dots represent non-avian theropod genera. Blue dots represent extant avian genera.

The aspect ratio of the tarsometatarsus can be indicative of hindlimb function (Madan et al., 2017). Superficially, the proportions of avisaurid tarsometatarsi are similar to spheniscids (penguins) [*A. archibaldi*. *A. darwini*, *Mirarce*, and *Gettyia* ~ 1:4, *Pygoscelis adeliae* 1:2.5, *Spheniscus humboldti* 1:2, *Eudyptes chrysocome* 1:2, *Waimanu manneringi* (CM zfa 35) 1:5, *Kupoupou stilwelli* (NMNZ S.47312) 1:3, *Delphinornis larseni* (IB/P/B-0062) 1:3], whose tarsometatarsi are robust and proximodistally reduced (Bloklan et al., 2019; Jadwiszcak and Mörs, 2019; Slack et al., 2006). On land, the short

hindlimbs of spheniscids facilitate a waddling gait, which is biomechanically efficient over long distances (Griffin and Kram, 2000). Though present in many other extant birds, notably spheniscid tarsometatarsi (particularly immature individuals) exhibit proportionally large lateral proximal vascular foramina. This structure may be functionally analogous to the intermetatarsal space between metatarsals III and IV in *A. darwini*, and *Soroavisaurus*. This space houses the lateral proximal intermetatarsal vein which eventually anastomoses with the common dorsal metatarsal vein on the dorsal side of the tarsometatarsus (Schwehn et al., 2024).

However, avisaurid tarsometatarsi are otherwise quite different from penguins, which lack a hypertrophied attachment for the *m. tibialis cranialis*, have much more well-developed intertrochlear space, and possess an unmodified, ginglymous metatarsal IV trochlea. Furthermore, evidence from *Mirarce* indicates that the overall skeletal anatomy is very different from spheniscids, lacking modified forelimbs and other morphologies associated with the specialized ecology of spheniscids.

Small bodied strigids (< 350 g) exhibit tarsometatarsi aspect ratios even closer to avisaurids (e.g., *Surnia ulula*, 1:4, *Aegolius funereus*, 1:5, *Glaucidium brasiliensis* 1:5) (Fig. 8). Together with other features of the tarsometatarsus, this similarity suggests small strigids may be the best functional analog for understanding hindlimb function in avisaurids. Strigids are able to produce greater grip force than other similar sized birds of prey (Fowler et al., 2009; Tronrud, 2022; Ward et al., 2002). The proportionally longer tarsometatarsi of accipitrids (aspect ratios of 1:20 – 1:10) are better suited for rapid movements (i.e., grabbing, snatching), whereas the proportionally stouter tarsometatarsi of strigids are better equipped for forceful grip and crushing (Tronrud, 2022; Ward et al., 2002). By lowering the aspect ratio of the tarsometatarsus, strigids reduce the length between the fulcrum (ankle joint), moment of action (muscle tendon insertion), and the resistance force (the prey), therefore reducing the required muscular force (i.e., torque) to lift or grip prey compared to longer-legged accipitrids (Vogel, 2013). In combination with having more mechanically efficient legs for producing force, strigids also possess mediolaterally wider muscle tendons and proportionally larger flexor muscles (e.g., *m. flexor digitorum longus*) than similarly sized accipitrids (Fowler et al., 2009; Tronrud, 2022; Volkov, 2004; Ward et al.,

2002). The lower aspect ratio of the tarsometatarsus accommodates these wide muscle tendons, resulting in more applied force (i.e., contraction) over a given area. The *m. flexor digitorum longus*, which is responsible for contracting pedal digits II-IV, is the largest observable lower hindlimb muscle in strigids; the muscle belly, located on the caudal surface tibiotarsus, is so large that it also protrudes around the medial and lateral borders of the tibiotarsus (Volkov, 2004; Ward et al., 2002). The combination of mediolaterally wider tendon insertions on the tarsometatarsus and greater potential force from increased flexor muscle mass results in comparatively more powerful and efficient pedal grip.

FIGURE 8. A) The left tarsometatarsi in dorsal and distal aspects with the *m. tibialis cranialis* tubercle highlighted in a) *Struthio camelus* (terrestrial ratite), b) *Turdus olivator* (arboreal passerine), c) *Cygnus buccinator* (semi-aquatic anseriform), d) *Buteo jamaicensis* (raptorial accipitrid), e) *Surnia ulula* (raptorial strigid), f) *Avisaurus darwini*. The distal view of each taxon is represented underneath the dorsal view to show variations of the intertrochlear grooves. Tarsometatarsi are not to scale. Reference photos of tarsometatarsi sourced from morphosource. B) Tarsometatarsus length (in meters) against the proportional distal location of the *m. tibialis cranialis* tubercle in extant birds and enantiornithines, including avisaurids (marked as asterisks). Extant birds are divided into their primary means of locomotion, following data from **Zeffer and Norberg (2003**).

Similarly, the tarsometatarsi of avisaurids exhibit lower aspects ratios than all other enantiornithines (with the exception of *Bauxitornis*), suggesting that members within this clade had similarly powerful flexion of DII-IV (Dyke and Osi, 2010). However, strigids are zygodactyl (with digits I and IV reversed), whereas avisaurids and accipitrids are anisodactyl (only digit I reversed). In contrast to strigids, accipitrids have proportionally larger insertions of the *m. flexor hallicus longus* tendons (Volkov, 2004). This permits greater force given to the hallux upon contraction, necessary for it to oppose the combined forces generated by the three forward facing digits (II-IV) (Volkov, 2004; Ward et al., 2002). The mediolaterally wide and well-developed plantar excavation of the tarsometatarsus in large avisaurids

(e.g., *Mirarce*, *Gettyia*, *Avisaurus*) is a space to accommodate mediolaterally expanded muscle tendons (of the *m. flexor hallucis longus* and *m. flexor digitorum longus*) as well as presence of a large *m. flexor hallucis brevis* belly (originating on the proximal part of the plantar surface), facilitating greater contractile grip. Avisaurids appear to unite the pedal arrangement of accipitrids and the mechanical benefits of the low aspect ratio tarsometatarsi in strigids.

Additionally, the well-developed, shelf-like labrum along the proximoplantar surface suggests the presence of an in-vivo cartilaginous hypotarsus, similar to that described in *Confuciusornis* (Jiang et al., 2017). If present, this feature would both aid in supporting the tendons of the digital flexor muscles and function as a moment arm for the ankle joint, allowing for greater tendon stability and ventroflexion of the lower hindlimb, respectively (Clifton and Carr, 2018; Mayr, 2016).

Functional Significance of the *M. Tibialis Cranialis* Tubercle

Present in confuciusornithiforms, ornithuromorphs, and enantiornithines, the tubercle on metatarsal II has been hypothesized to represent the insertion point for the *m. tibialis cranialis* (Chiappe and Walker, 2002). This feature is particularly pronounced (dorsally projecting) in enantiornithines, present in nearly all taxa in which preservation allows observation. Interestingly, this feature is noticeably absent in non-avian theropods, and located on metatarsal III in a select number of Mesozoic birds (e.g., *Mystiornis*, *Confuciusornis*, *Ichthyornis*) (O'Connor et al., 2014). In extant birds, the *m. tibialis cranialis* originates on the proximocranial surface of the tibiotarsus and inserts on the dorsoproximal surface of the tarsometatarsus (Moreno and Carrascal, 1993; Zeffer and Norberg, 2003). As the muscle tendon dorsodistally extends from the distal tibiotarsus to the tarsometatarsus, it is secured in place by a retinaculum on the craniodistal surface of the tibiotarsus, just proximal to the ankle joint (Moreno and Carrascal, 1993; Zeffer and Norberg, 2003). Contraction of this muscle dorsiflexes the ankle, bringing the dorsal face of the tarsometatarsus towards the cranial surface of the tibiotarsus (Moreno and Carrascal, 1993; Zeffer and Norberg, 2003). The larger the insertion point (i.e., tubercle), the larger the

corresponding muscle tendon, resulting in more powerful dorsiflexion. Likewise, the more distal the insertion point, the greater the applied force relative to the fulcrum (ankle joint) to dorsiflex the ankle or compensate for the weight of an object held in the pes (Gaslow, 1967; Moreno and Carrascal, 1993; Vogel, 2013) (Fig. 9).

FIGURE 9. A) Reconstruction of an articulated left tibiotarsus, tarsometatarsus, and pes of *Avisaurus darwini* with muscular reconstruction of the primary pedal flexors (caudal surface) and *m. tibialis cranialis* (cranial surface) in medial aspect. A) ankle joint shown in neutral position. B) ankle joint shown in dorsiflexion. Tibiotarsus and pedal elements based on *Mirarce*, but scaled to appropriate size (**Atterholt et al., 2018**). Muscles such as the gastrocnemius and extensors are not included to more clearly show the two major flexor muscles and the *m. tibialis cranialis*. The tibialis cranialis muscle attaches to the hypertrophied tubercle of metatarsal II. The action of the tibialis cranialis is flexedit is to dorsiflex the ankle and invert the tarsometatarsus. This feature is hypertrophied in birds of prey compared to other extant birds, and is associated with an increased capacity to grasp prey with the pes. C) A member of the genus *Glaucidium* exhibiting the carrying strength that comes from having an enlarged and distally-migrated *m. tibialis cranialis* tubercle. Within this taxon, prey items may exceed three times the body mass of the owl (**Billerman et al., 2022; del Hoyo, 1999**). Photo used with permission and credited to Roy Priest.

Variation in the location of this muscle attachment among extant birds makes it a useful osteological correlate for determining interaction between substrate (or prey) and the pedes (Moreno and Carrascal, 1993; Zeffer and Norberg, 2003). Dorsiflexion is necessary in maintaining balance by lowering the center of mass when perching, which is why this feature is often more well-developed and proximally located in passerines than in terrestrial birds (Zeffer and Norberg, 2003), and similarly why this feature is well-developed in arboreal enantiornithines (Fig. 8). Extant birds that use their legs for swimming have even more prominent *m. tibialis cranialis* tubercles that are more distally located than perching birds.

This facilitates dorsiflexion of the foot against the resistance of water, to subsequently extend and produce thrust (i.e., paddling). However, raptorial birds exhibit both the largest and most distally-located *m. tibialis cranialis* tubercles (Zeffer and Norberg, 2003) (Fig. 8). Similar to the relationship between raptorial and non-raptorial birds, avisaurid *m. tibialis cranialis* tubercles differ from other enantiornithines in being larger and more distally located (Fig. 8; Table S3).

Variation in both the size and distal location of this muscle attachment among raptorial birds reflects differences in acquisition and proportional size of prey (Volkov, 2004; Zeffer and Norberg, 2003). Along with the size and distal location of the tubercle, the relative medial location on the tarsometatarsus also affects the degree of pedal inversion that occurs when the *m. tibialis cranialis* is contracted (Fig. 10). Inversion of the pes while grasping prey increases grip stability as the force being generated pushes against the opposing foot. The more medial the location of the tubercle, the greater the degree of pedal inversion (Volkov, 2004; Zeffer and Norberg, 2003). Compared to accipitrids and falconids, strigids also have the most distomedially located *m. tibialis cranialis* tubercles. In particular, smaller bodied owls, like *Glaucidium* tend to exhibit greater distomedial migration of the attachment compared to larger strigids, further aiding in supporting proportionally larger prey when in flight. For example, *Glaucidium* (Pygmy owls), though diminutive (up to ~ 80 g) will subdue and travel with prey items that often rival or exceed their own body mass (e.g., *Microtus* sp., 60 g; *Coccothraustes* sp., 60 g; *Callipepla sp.*, 160 g) (Billerman et al., 2022; Zeffer and Norberg 2003) (Fig. 9B).

FIGURE 10. Action of the tibialis cranialis muscle on the ankle joint, shown in a A) resting position and a B) contracted position. As the muscle contracts, not only does the ankle dorsiflex, bringing the tibiotarsus and tarsometatarsus closer together, but the tarsometatarsus and phalanges invert (i.e., elevation of the medial border of the foot).

The *m. tibialis cranialis* tubercle in avisaurids is larger and more distomedially located on the tarsometatarsus than in any Mesozoic bird, non-avian theropod, and nearly every extant bird (Brett-

Surman and Paul, 1985). The presence of the large distomedially located *m. tibialis cranialis* tubercle in avisaurids, together with the lower aspect ratio of the tarsometatarsus, suggests a functional similarity that distinguishes them from other enantiornithines. Based on the numerous shared morphologies with strigids, avisaurid tarsometatarsi were likely adapted for strong dorsiflexion and carrying proportionally large prey items while volant (Fig. 11). Among avisaurids, *Gettyia* (Fig. 4D) exhibits the proportionally distal-most *m. tibialis cranialis* tubercle. It is also the smallest known avisaurid (~540 g), with a tarsometatarsal length that is less than half that of *A. darwini* (~1700 g) (Atterholt et al., 2018) (Fig. 8A). This suggests that like diminutive strigids, *Gettyia* was adapted for subduing and carrying proportionally large prey (e.g., *Surnia*, *Glaucidium*, *Aegolius*) (Zeffer and Norberg, 2003).

FIGURE 11. Reconstruction of an avisaurid (e.g., *A. darwini*). Morphology of the tarsometatarsus suggests that these large birds engaged in raptorial behavior and could carry proportionally large prey.

Significance of Trochlear Groove Development

Strigids exhibit well-developed and deeply-grooved metatarsal trochleae, which correspond to both proportionally longer and more robust pedal digits than similarly sized accipitrids (Einoder and Richardson, 2007; Madan et al., 2017; Ward et al., 2002) (Fig. 8). This grooved morphology exhibits distinctly developed excavations between the medial and lateral condyles. The deeply-grooved metatarsal trochlea corresponds to phalanges with greater-projecting intercotylar crests, and excavated articular cotyla. This morphology results in a larger articular surface between the proximal phalanges and the tarsometatarsus, resulting in a restricted range of motion at the metatarsophalangeal joint when the flexor tendons contract, (i.e., gripping an object) (Madan et al., 2017; Tronrud, 2022; Usherwood et al., 2014). Pedal digits II-IV of strigids have mediolaterally wider tendons of the *m. flexor digitorum* than in accipitrids, facilitating stronger contraction and therefore grip strength (Madan et al., 2017; Usherwood et al., 2014). Along with the low aspect ratio of the tarsometatarsus, the more robust pedal digits of strigids

are suggested to aid in crushing and importantly, securing proportionally large prey in conditions where visual acuity is low (i.e., low light) (Fowler et al., 2009; Goslow, 1967, 1972; Madan et al., 2017; Tronrud, 2022). Unlike diurnal accipitrids, pursuit of prey in low light conditions makes recapture unlikely should prey escape upon initial attack, making lethal first contact imperative (Goslow, 1967, 1972; Madan et al., 2017). This may explain why strigiforms have been found to apply more crushing force than is necessary to kill their prey items (Madan et al., 2017; Usherwood et al., 2014) Well-developed (compared to other enantiornithines) trochlear grooves are present in the mediolaterally wide trochleae of metatarsals II and III of *A. archibaldi*, *A. darwini*, *Mirarce*, *Soroavisaurus*, and *Gettyia*, suggesting similarly robust and powerful pedal digits (Atterholt et al., 2018; Brett-Surman & Paul, 1985; Einoder and Richardson, 2007; Madan et al., 2017; Tronrud, 2022). Together with the absolute larger size of avisaurids compared to other enantiornithines, a proportionally high level of applied pedal digit pressure (i.e., grip) is plausible for members of this clade.

Conclusion

Here we describe three new enantiornithines, two of which are new avisaurids, all from exposures of the latest Cretaceous Hell Creek Formation in Montana. Both new avisaurids are larger than the largest Early Cretaceous enantiornithine. This supports the apparent trend towards increased body mass diversity in the Late Cretaceous among enantiornithines with large-bodied forms being most heavily represented in the Late Cretaceous fossil record. By increasing body size, this unique bird lineage explored new ecospaces suggesting that enantiornithines were still diversifying near the end Cretaceous. The morphology of the avisaurid tarsometatarsus suggests some Late Cretaceous enantiornithines adopted raptorial ecologies. Avisaurid tarsometatarsi exhibit low aspect ratios, proportionally large and distomedially located *m. tibialis cranialis* tubercles, and well-developed grooved metatarsal trochleae. All of these morphologies are uniquely found in members of extant raptorial birds, specifically strigids, and

suggest similar powerful pedal constriction behaviors albeit in an anisodactyl pedal formation. By combining morphological aspects of both extant accipitrids and strigids, avisaurids represent a group of enantiornithines in which modern analogues do not exist.

Supporting Information

FIGURE S1. Analysis of 863 extant birds (317 species) measuring mass in grams against length of the tarsometatarsus. Data from Field et al., (2013).

FIGURE S2. Plotting three avisaurids to approximate mass. Utilizing the dataset from Field et al., (2013), three avisaurids (*A. archibaldi, A. darwini*, *Mirarce*) were plotted against (A) all sampled extant birds, and then against all birds, (B) by family. Once closely associated families were identified, an averaged mass estimate could be calculated using data collected within Field et al., (2013).

TABLE S1. Sampled enantiornithine tarsometatarsi length and width at the midpoint (mm).

TABLE S2. Field et al. (2013) measurements used for weight estimations in extant birds and predictive masses for selected avisaurids based on averaged results.

TABLE S3. Dataset from Zeffer and Norberg (2003) with added enantiornithines to assess distal location of the *m. tibialis cranialis* tubercle.

TABLE S4. Dataset used for comparative aspect ratios of the tarsometatarsus between avian and non-avian theropods. All extant taxa used are from Field et al., 2013.

Acknowledgements

We would like to acknowledge the field crews of Nathan Carroll (Carter County Museum), Thomas Carr (Dinosaur Discovery Museum), and Frankie Jackson (Montana State University). Without their effort, these new taxa would not have been found. Thank you to Daigo Yamamura for providing info on the collection of MOR 3070. Thank you to the Bureau of Land Management and Montana Fish, Wildlife & Parks for providing access to lands where these discoveries were made. Thank you to Samantha Clark who illustrated the tarsometatarsi in figures 1 – 3, and to Ville Sinkkonen who illustrated figure 11. Thank you to Akiko Shinya for assistance in additional preparation of the fossils.

References

Abourachid, A., Fabre, A., Cornette, R., Höfling, E., 2017. Foot shape in arboreal birds: two morphological patterns for the same pincer‐like tool. Journal of Anatomy 231, 1‐11. https://doi.org/10.1111/joa.12614

Atterholt, J., Hutchison, J.H., O'Connor, J.K., 2018. The most complete enantiornithine from North America and a phylogenetic analysis of the Avisauridae. PeerJ 6, e5910. https://doi.org/10.7717/peerj.5910

Bailleul, A.M., O'Connor, J., Zhang, S., Li, Z., Wang, Q., Lamanna, M.C., Zhu, X., Zhou, Z., 2019. An Early Cretaceous enantiornithine (Aves) preserving an unlaid egg and probable medullary bone. Nat Commun 10, 1275. https://doi.org/10.1038/s41467-019-09259-x

Baumel, J.J., Witmer , L.M., 1993. Osteologia, in: Handbook or Avian Anatomy. Nuptial Ornithological Club, Cambridge, Massachusetts.

Biewener, A.A., 2011. Muscle function in avian flight: achieving power and control. Phil. Trans. R. Soc. B 366, 1496–1506. https://doi.org/10.1098/rstb.2010.0353

Billerman, S.M., Keeney, B.K., Rodewald, P.G., Schulenberg, T.S., 2022. Birds of the World. Cornell Lab of Ornithology, Ithaca, NY, USA.

Blokland, J., Reid, C., Worthy, T., Tennyson, A., Clarke, J., Scofield, R., 2019. Chatham Island Paleocene fossils provide insight into the palaeobiology, evolution, and diversity of early penguins (Aves, Sphenisciformes). Palaeontol Electron. https://doi.org/10.26879/1009

Brett-Surman, M.K., Paul, G.S., 1985. A new family of bird-like dinosaurs linking Laurasia and Gondwanaland. Journal of Vertebrate Paleontology 5, 133–138. https://doi.org/10.1080/02724634.1985.10011851

Buscalioni, A.D., Poyato-ariza, F.J., 2016. Las Hoyas: a unique Cretaceous ecosystem. New Mexico Museum of Natural History and Science Bulletin.

Cau, A., Arduini, P., 2008. Enantiophoenix electrophyla gen. et sp. nov. (Aves, Enantiornithes) from the Upper Cretaceous (Cenomanian) of Lebanon and its phylogenetic relationships. Atti Soc. it. Sci. nat. Museo civ. Stor. nat. Milano 149, 293–324.

Chiappe, L., 1993. Enantiornithine (Aves) Tarsometatarsi from the Cretaceous Lecho Formation of Northwestern Argentina. American Museum Novitates 3083.

Chiappe, L.M., 1992. Enantiornithine (Aves) tarsometatarsi and the avian affinities of the Late Cretaceous Avisauridae. Journal of Vertebrate Paleontology 12, 344–350. https://doi.org/10.1080/02724634.1992.10011464

Chiappe, L.M., Calvo, J.O., 1994. *Neuquenornis volans*, a new Late Cretaceous bird (Enantiornithes: Avisauridae) from Patagonia, Argentina. Journal of Vertebrate Paleontology 14, 230–246. https://doi.org/10.1080/02724634.1994.10011554

Chiappe, L.M., Walker, C.A., 2002. Skeletal morphology and systematics of the Cretaceous Euenantiornithines (Ornithothoraces: Enantiornithes)., in: Mesozoic Birds: Above the Heads of Dinosaurs. University of California Press, Berkley, CA, p. pp 240-267.

Clifton, G.T., Carr, J.A., Biewener, A.A., 2018. Comparative hindlimb myology of foot-propelled swimming birds. Journal of Anatomy 232, 105–123. https://doi.org/10.1111/joa.12710

del Hoyo, J., Elliot, A., Sargatal, J., Collar, N.J., 1999. Handbook of the birds of the world. Volume 5. Lynx edicions, Barcelona.

De Mendoza, R.S., Gómez, R.O., 2022. Ecomorphology of the tarsometatarsus of waterfowl (Anseriformes) based on geometric morphometrics and its application to fossils. The Anatomical Record 305, 3243–3253. https://doi.org/10.1002/ar.24891

Dyke, G.J., Ősi, A., 2010. A review of Late Cretaceous fossil birds from Hungary. Geological Journal 45, 434–444. https://doi.org/10.1002/gj.1209

Einoder, L.D., Richardson, A.M.M., 2007. Aspects of the Hindlimb Morphology of Some Australian Birds of Prey: A Comparative and Quantitative Study. The Auk 124, 773–788. https://doi.org/10.1093/auk/124.3.773

Field, D.J., Lynner, C., Brown, C., Darroch, S.A.F., 2013. Skeletal Correlates for Body Mass Estimation in Modern and Fossil Flying Birds. PLoS ONE 8, e82000. https://doi.org/10.1371/journal.pone.0082000

Fowler, D.W., Freedman, E.A., Scannella, J.B., 2009. Predatory Functional Morphology in Raptors: Interdigital Variation in Talon Size Is Related to Prey Restraint and Immobilisation Technique. PLoS ONE 4, e7999. https://doi.org/10.1371/journal.pone.0007999

Fowler, D., 2020. The Hell Creek Formation, Montana: A stratigraphic review and revision based on a sequence stratigraphic approach. Geosciences, 10(11), p.435.

Goslow, G.E., 1972. Adaptive Mechanisms of the Raptor Pelvic Limb. The Auk 89, 47–64. https://doi.org/10.2307/4084059

Goslow, G.E., 1967. FUNCTIONAL ANALYSIS OF THE STRIKING MECHANISMS OF RAPTORIAL BIRDS. University of California Davis.

Goloboff, P.A., Farris, J.S., Nixon, K.C., 2008. TNT, a free program for phylogenetic analysis. Cladistics 24, 774–786. https://doi.org/10.1111/j.1096-0031.2008.00217.x

Grant, P.R., 1971. Variation in the Tarsus Length of Birds in Island and Mainland Regions. Evolution 25, 599. https://doi.org/10.2307/2406943

Griffin, T.M., Kram, R., 2000. Penguin waddling is not wasteful. Nature 408, 929–929. https://doi.org/10.1038/35050167

Höfling, E., Abourachid, A., 2021. The skin of birds' feet: Morphological adaptations of the plantar surface. Journal of Morphology 282, 88–97. https://doi.org/10.1002/jmor.21284

Hone, D.W.E., Dyke, G.J., Haden, M., Benton, M.J., 2008. Body size evolution in Mesozoic birds. J of Evolutionary Biology 21, 618–624. https://doi.org/10.1111/j.1420-9101.2007.01483.x

Hu, H., Wang, Y., Fabbri, M., O'Connor, J.K., McDonald, P.G., Wroe, S., Yin, X., Zheng, X., Zhou, Z., Benson, R.B.J., 2023. Cranial osteology and palaeobiology of the Early Cretaceous bird Jeholornis prima (Aves: Jeholornithiformes). Zoological Journal of the Linnean Society 198, 93–112. https://doi.org/10.1093/zoolinnean/zlac089

Jadwiszczak, P., Mörs, T., 2019. First partial skeleton of Delphinornis larseni Wiman, 1905, a slender-footed penguin from the Eocene of Antarctic Peninsula. Palaeontol Electron. https://doi.org/10.26879/933

Jadwiszczak, P., 2015. Another look at tarsometatarsi of early penguins. Polish Polar Research 36, 343–354. https://doi.org/10.1515/popore-2015-0024

Jiang, B., Zhao, T., Regnault, S., Edwards, N.P., Kohn, S.C., Li, Z., Wogelius, R.A., Benton, M.J., Hutchinson, J.R., 2017. Cellular preservation of musculoskeletal specializations in the Cretaceous bird Confuciusornis. Nat Commun 8, 14779. https://doi.org/10.1038/ncomms14779

Longrich, N.R., Tokaryk, T., Field, D.J., 2011. Mass extinction of birds at the Cretaceous–Paleogene (K–Pg) boundary. Proc. Natl. Acad. Sci. U.S.A. 108, 15253–15257. https://doi.org/10.1073/pnas.1110395108

Madan, M.A., Rayfield, E.J., Bright, J.A., 2017. Scaling and functional morphology in strigiform hind limbs. Sci Rep 7, 44920. https://doi.org/10.1038/srep44920

Martin, E.M., Sherratt, E., 2023. Grasping hold of functional trade-offs using the diversity of foot forms in Australian birds. Evol Ecol 37, 945–959. https://doi.org/10.1007/s10682-023-10261-5

Mayr, G., 2016. Variations in the hypotarsus morphology of birds and their evolutionary significance. Acta Zoologica 97, 196–210. https://doi.org/10.1111/azo.12117

Mohamed, R., 2019. Histomorphological study on the tongue of the duck in the Caribbean with relation to feeding habit and #8195; J Adv Vet Anim Res 74. https://doi.org/10.5455/javar.2019.f315

Moreno, E., Carrascal, L.M., 1993. Leg Morphology and Feeding Postures in Four Parus Species: An Experimental Ecomorphological Approach. Ecology 74, 2037–2044. https://doi.org/10.2307/1940849

Novas, F.E., Agnolín, F.L., Scanferla, C.A., 2010. New enantiornithine bird (Aves, Ornithothoraces) from the Late Cretaceous of NW Argentina. Comptes Rendus Palevol 9, 499–503. https://doi.org/10.1016/j.crpv.2010.09.005

O'Connor, J.K., Chiappe, L.M., 2011. A revision of enantiornithine (Aves: Ornithothoraces) skull morphology. Journal of Systematic Palaeontology 9, 135–157. https://doi.org/10.1080/14772019.2010.526639

O'Connor, J., 2022. Enantiornithes. Current Biology 32, R1166–R1172. https://doi.org/10.1016/j.cub.2022.07.074

O'Connor, J.K., Wang, X., Chiappe, L.M., Gao, C., Meng, Q., Cheng, X., Liu, J., 2009a. Phylogenetic support for a specialized clade of Cretaceous enantiornithine birds with information from a new species. Journal of Vertebrate Paleontology 29, 188–204. https://doi.org/10.1080/02724634.2009.10010371

O'Connor, J.K., Wang, X., Chiappe, L.M., Gao, C., Meng, Q., Cheng, X., Liu, J., 2009b. Phylogenetic support for a specialized clade of Cretaceous enantiornithine birds with information from a new species. Journal of Vertebrate Paleontology 29, 188–204. https://doi.org/10.1080/02724634.2009.10010371

O'Connor, J.K., Clark. , A.D., Herrera, F., Zhou, Z., Xiaoli , W., Zheng, X., 2023b. LONGIPTERYX STOMACH CONTENTS INDICATE THAT MORPHOLOGY DOES NOT ACCURATELY PREDICT DIET IN ENANTIORNITHINES. Cincinnati, OH

O'Connor, J.K., Averianov, A.O., Zelenkov, N.V., 2014. A CONFUCIUSORNITHIFORM (AVES, PYGOSTYLIA)-LIKE TARSOMETATARSUS FROM THE EARLY CRETACEOUS OF SIBERIA AND A DISCUSSION OF THE EVOLUTION OF AVIAN HIND LIMB MUSCULATURE. Journal of Vertebrate Paleontology 34.

O'Connor, J.K., Li, D.-Q., Lamanna, M.C., Wang, M., Harris, J.D., Atterholt, J., You, H.-L., 2016. A new Early Cretaceous enantiornithine (Aves, Ornithothoraces) from northwestern China with elaborate tail ornamentation. Journal of Vertebrate Paleontology 36, e1054035. https://doi.org/10.1080/02724634.2015.1054035 Schneider, C. A., Rasband, W. S., & Eliceiri, K. W. 2012. NIH Image to ImageJ: 25 years of image analysis. Nature Methods, 9(7), 671–675. doi:10.1038/nmeth.2089

Schwehn, R., Engelke, E., Seiler, C., Fischer, D., Seifert, H., Pfarrer, C., Fehr, M., Legler, M., 2024. Blood Vessel Topography of the Feet in Selected Species of Birds of Prey and Owls. Veterinary Sciences 11, 88. https://doi.org/10.3390/vetsci11020088

Slack, K.E., Jones, C.M., Ando, T., Harrison, G.L. (Abby), Fordyce, R.E., Arnason, U., Penny, D., 2006. Early Penguin Fossils, Plus Mitochondrial Genomes, Calibrate Avian Evolution. Molecular Biology and Evolution 23, 1144–1155. https://doi.org/10.1093/molbev/msj124

Torres, C.R., Norell, M.A., Clarke, J.A., 2021. Bird neurocranial and body mass evolution across the end-Cretaceous mass extinction: The avian brain shape left other dinosaurs behind. Sci. Adv. 7, eabg7099. https://doi.org/10.1126/sciadv.abg7099

Tronrud, B., 2022. Evolution and ecomorphology of the avian foot: the morphology of the tarsometatarsus in birds and its responses to evolutionary changes in foot use. University of Oxford.

Usherwood, J.R., Sparkes, E.L., Weller, R., 2014. Leap and Strike kinetics of an acoustically "hunting" barn owl Tyto alba. Journal of Experimental Biology jeb.107169. https://doi.org/10.1242/jeb.107169

Varricchio, D.J., Chiappe, L.M., 1995. A New Enantiornithine Bird From the Upper Cretaceous Two Medicine Formation of Montana. Journal of Vertebrate Paleontology 15, 201–204. https://doi.org/10.1080/02724634.1995.10011219

Vogel, S., 2013. Comparative biomechanics: life's physical world, Second edition. ed. Princeton University Press, Princeton.

Volkov, S.V., 2004. THE HINDLIMB MUSCULATURE OF THE TRUE OWLS (STRIGIDAE: STRIGIFORMES): MORPHOLOGICAL PECULIARITIES AND GENERAL ADAPTATIONS. Ornithologia 31.

Walker, C.A., 1981. New subclass of birds from the Cretaceous of South America. Nature 292, 51–53. https://doi.org/10.1038/292051a0

Wang, X., Clark, A.D., O'Connor, J.K., Zhang, X., Wang, X., Zheng, X., Zhou, Z. 2024. First edentulous enantiornithines (Aves: Ornithothoraces) from the Lower Cretaceous Jehol Avifauna. Cretaceous Research.

Ward, A.B., Weigl, P.D., Conroy, R.M., 2002. Functional Morphology of Raptor Hindlimbs: Implications for Resource Partitioning. The Auk 119, 1052–1063. https://doi.org/10.1093/auk/119.4.1052

Wu, Y.-H., Chiappe, L.M., Bottjer, D.J., Nava, W., Martinelli, A.G., 2021. Dental replacement in Mesozoic birds: evidence from newly discovered Brazilian enantiornithines. Sci Rep 11, 19349. https://doi.org/10.1038/s41598-021-98335-8

Xing, L., O'Connor, J.K., Chiappe, L.M., McKellar, R.C., Carroll, N., Hu, H., Bai, M., Lei, F., 2019. A New Enantiornithine Bird with Unusual Pedal Proportions Found in Amber. Current Biology 29, 2396-2401.e2. https://doi.org/10.1016/j.cub.2019.05.077

Xing, L., Cockx, P., O'Connor, J.K., Mckellar, R.C., 2020. A newly discovered enantiornithine foot preserved in mid-Cretaceous Burmese amber. PE 3, 212–219. https://doi.org/10.11646/palaeoentomology.3.2.11

Xu, X., Zhou, Z., Wang, Y., Wang, M., 2020. Study on the Jehol Biota: Recent advances and future prospects. Sci. China Earth Sci. 63, 757–773. https://doi.org/10.1007/s11430-019-9509-3

Yamamura, Daigo. Effects of depositional environment, asssemblage taphonomy and diagenesis on vertebrate skeletal preservation in a crevasse splay sandstone, upper Cretaceous Hell Creek Formation, eastern Montana. Diss. Montana State University-Bozeman, College of Letters & Science, 2013.

Zeffer, A., Norberg, U.M.L., 2003. Leg morphology and locomotion in birds: requirements for force and speed during ankle flexion. Journal of Experimental Biology 206, 1085–1097. https://doi.org/10.1242/jeb.00208

Zheng, X., O'Connor, J.K., Huchzermeyer, F., Wang, X., Wang, Y., Zhang, X., Zhou, Z., 2014. New Specimens of Yanornis Indicate a Piscivorous Diet and Modern Alimentary Canal. PLoS ONE 9, e95036. https://doi.org/10.1371/journal.pone.0095036

Zhou, Z., 2002. A new and primitive enantiornithine bird from the Early Cretaceous of China. Journal of Vertebrate Paleontology 22, 49–57. https://doi.org/10.1671/0272-4634(2002)022[0049:ANAPEB]2.0.CO;2

Zhou, Z., Clarke, J., Zhang, F., 2008. Insight into diversity, body size and morphological evolution from the largest Early Cretaceous enantiornithine bird. Journal of Anatomy 212, 565–577. https://doi.org/10.1111/j.1469-7580.2008.00880.x

Zhou, Z., Zhang, F., 2003. Anatomy of the primitive bird Sapeornis chaoyangensis from the Early Cretaceous of Liaoning, China. Can. J. Earth Sci. 40, 731–747. https://doi.org/10.1139/e03-011

Zhou, Z., Zhang, F., 2006. A beaked basal ornithurine bird (Aves, Ornithurae) from the Lower Cretaceous of China. Zoologica Scripta 35, 363–373. https://doi.org/10.1111/j.1463-6409.2006.00234.x

Figure 1

Click here to access/download;Figure;Figure 1.png

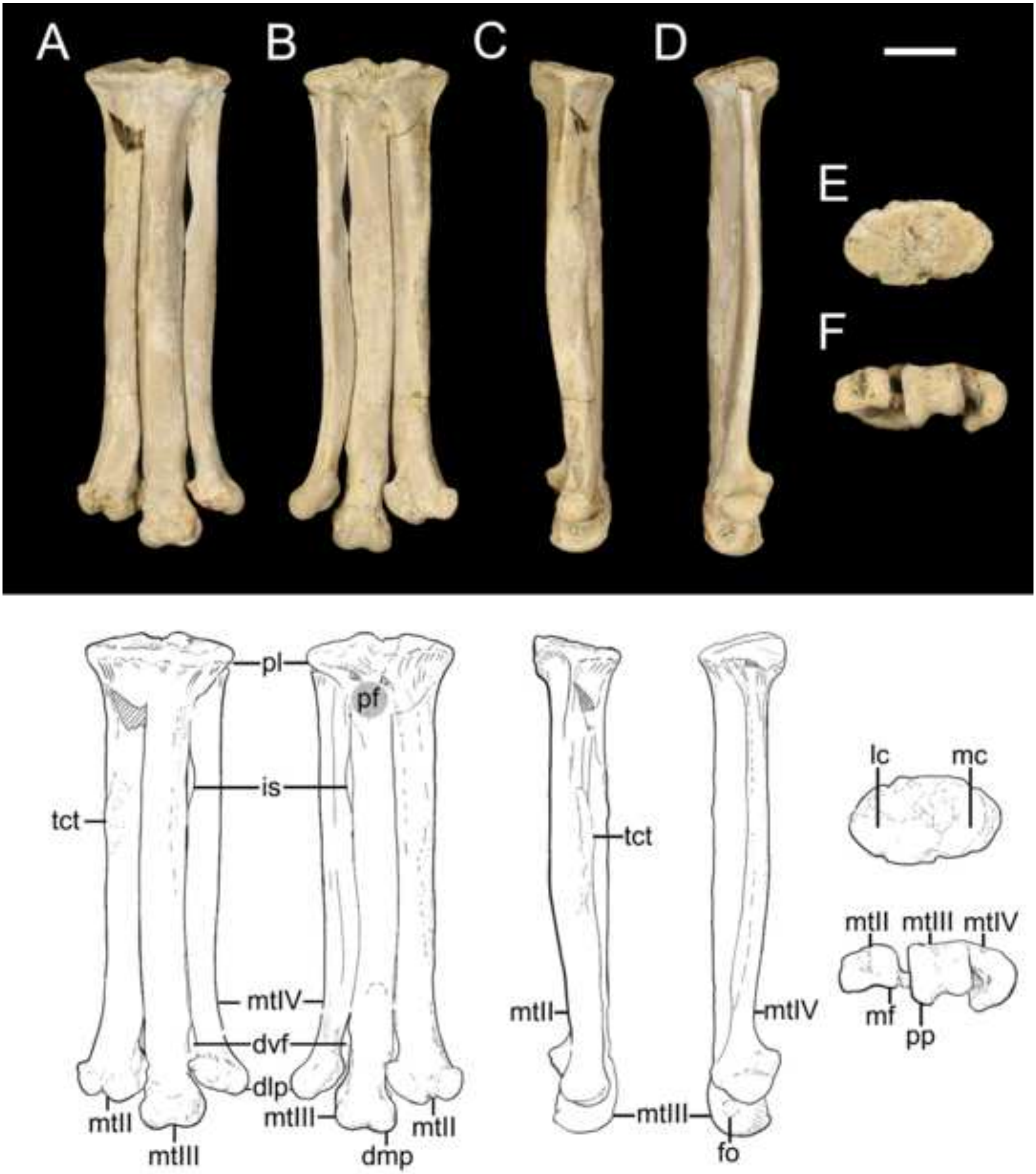

Figure 2

Click here to access/download;Figure;Figure 2.png

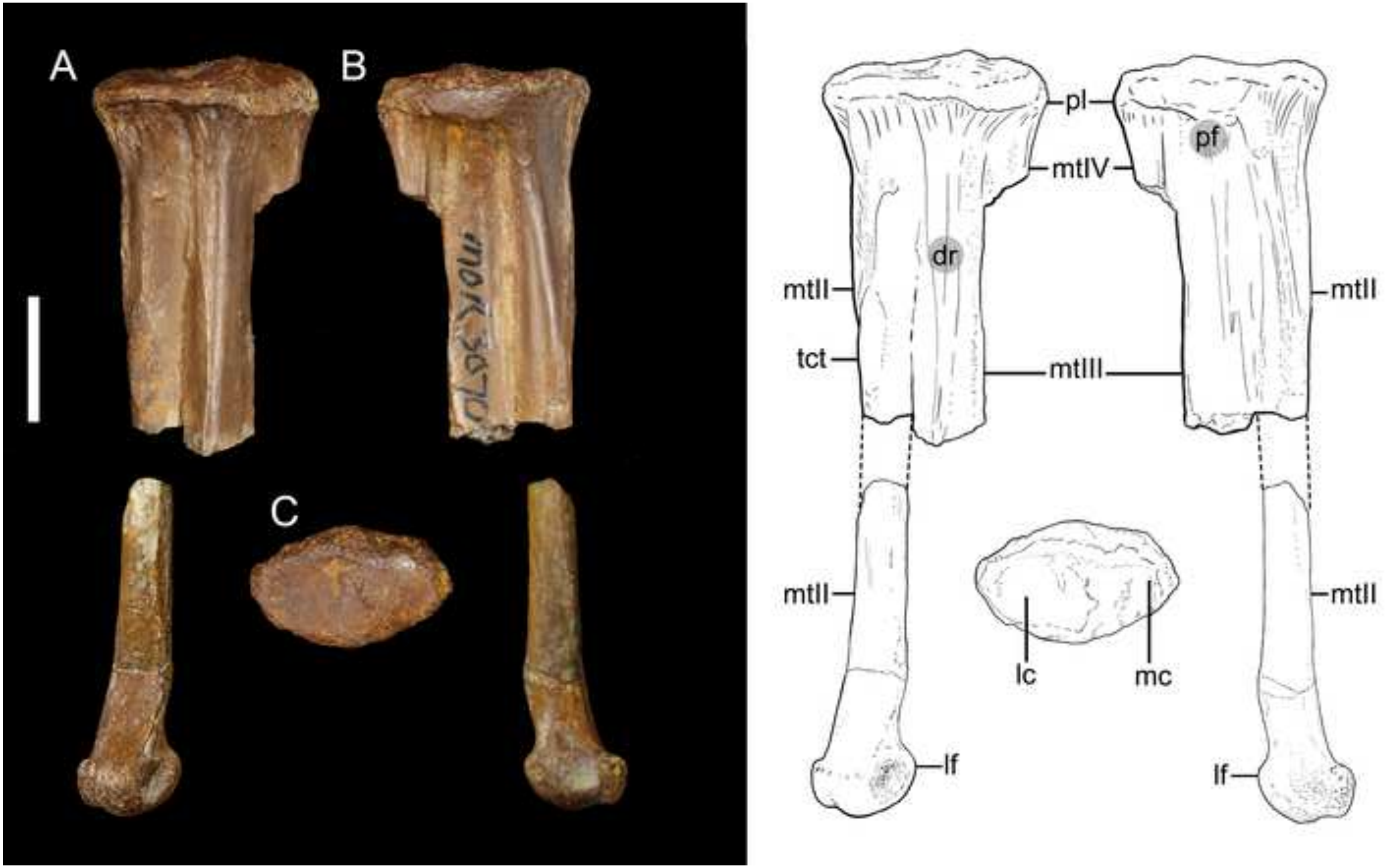

Figure 3

Click here to access/download;Figure;Figure 3.png

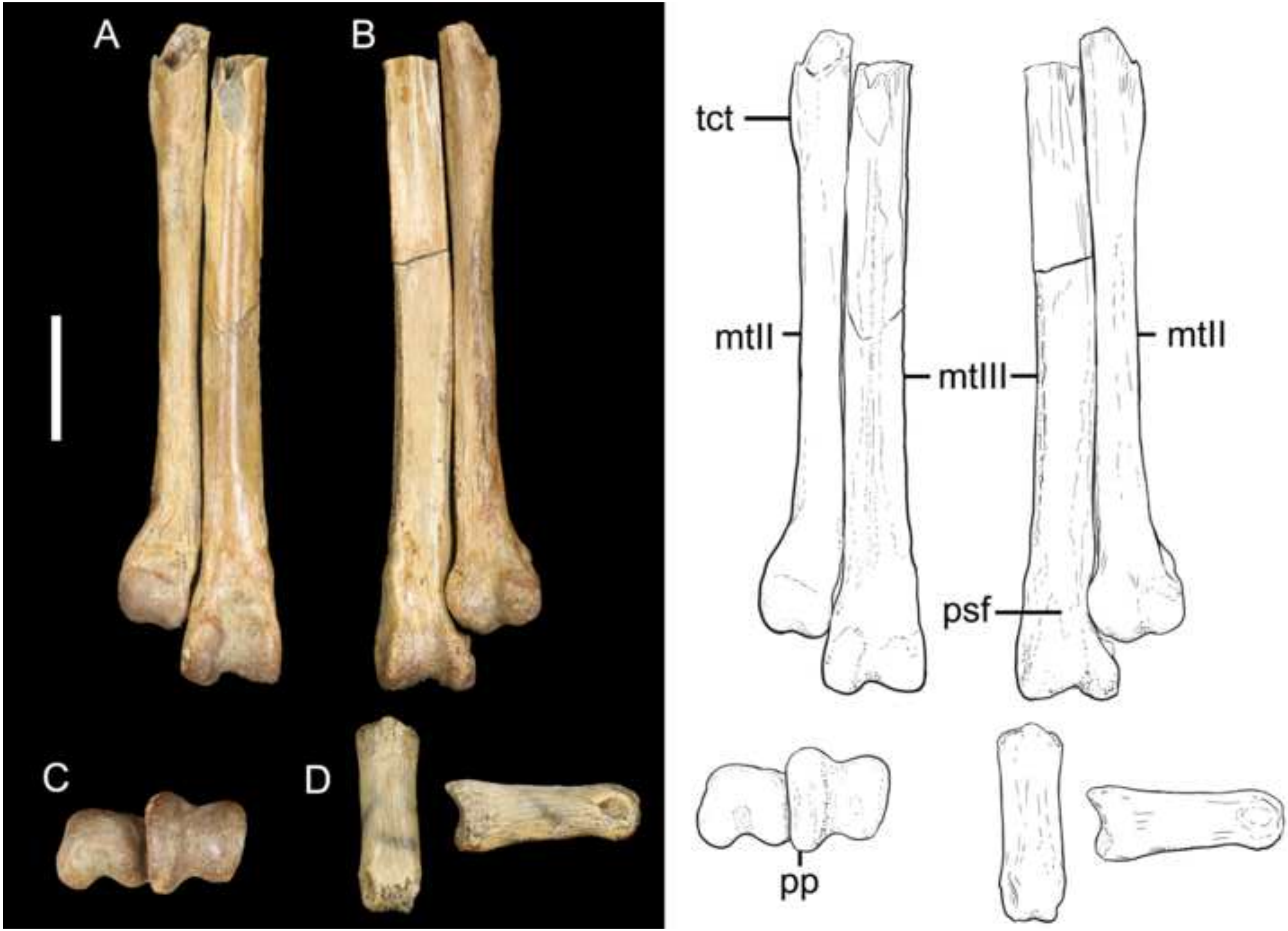

Figure 4

Click here to access/download;Figure;Figure 4.png

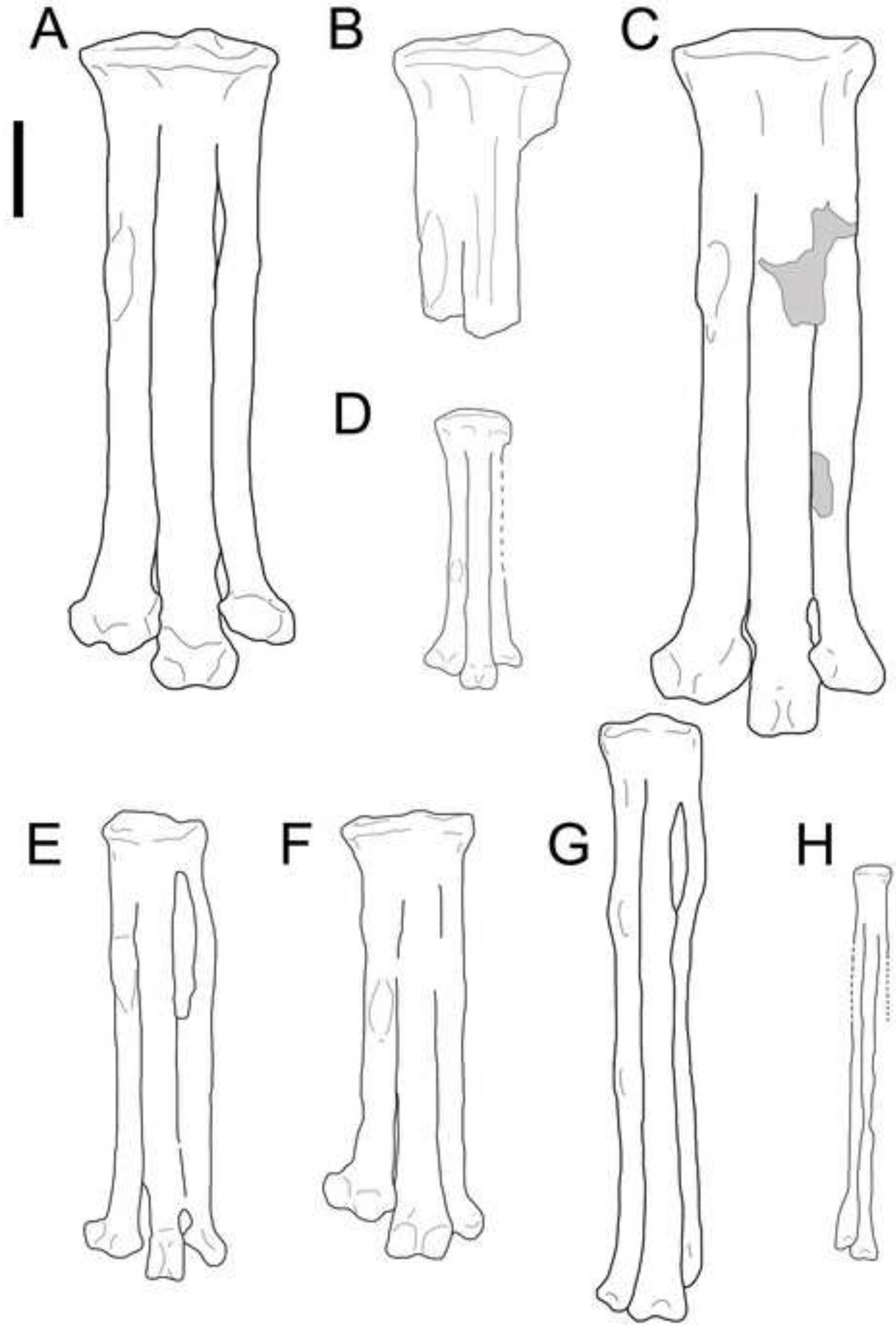

Figure 5

Click here to access/download;Figure;Figure 5.png

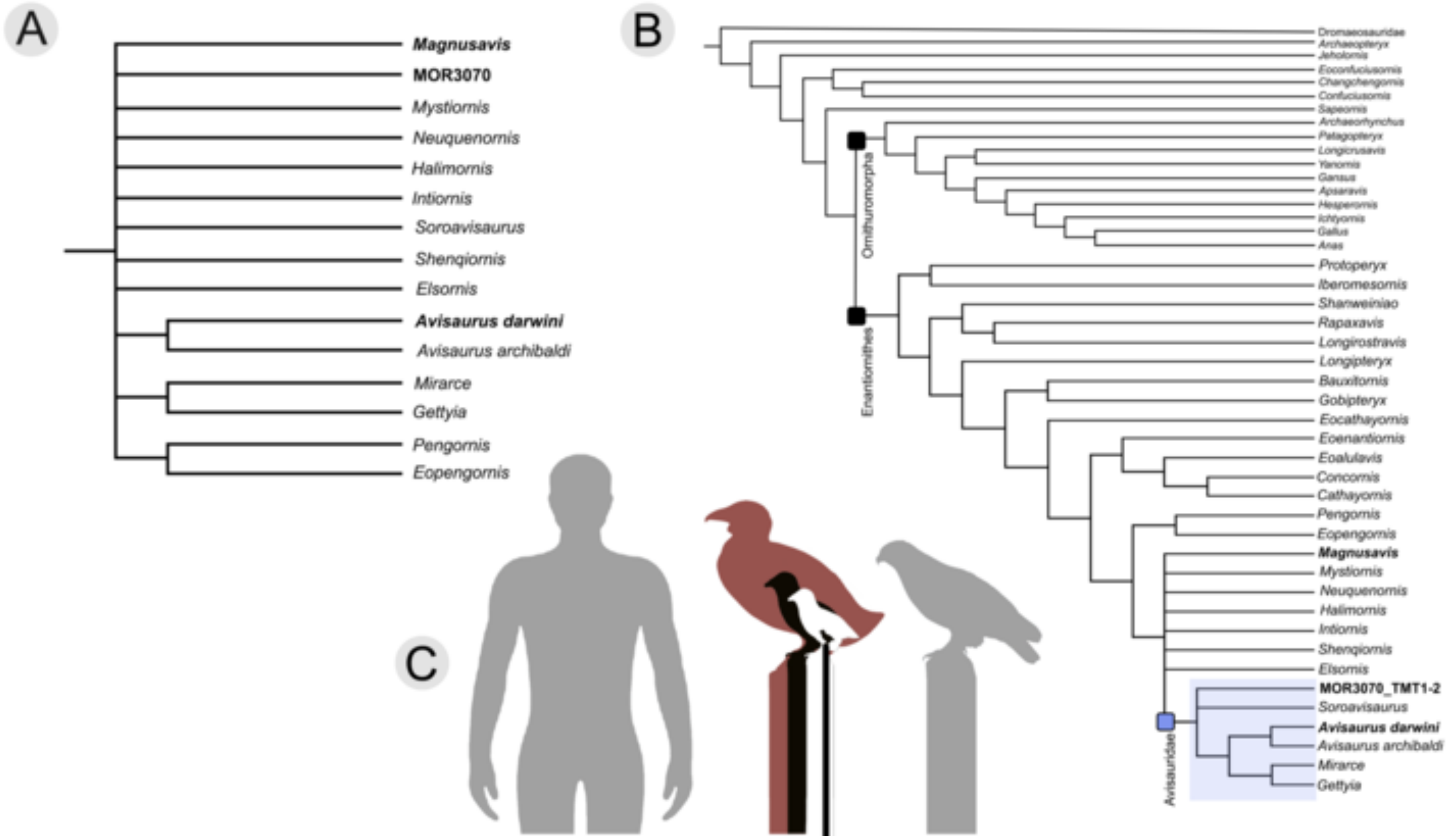

Figure 6

Click here to access/download;Figure;Figure 6.png

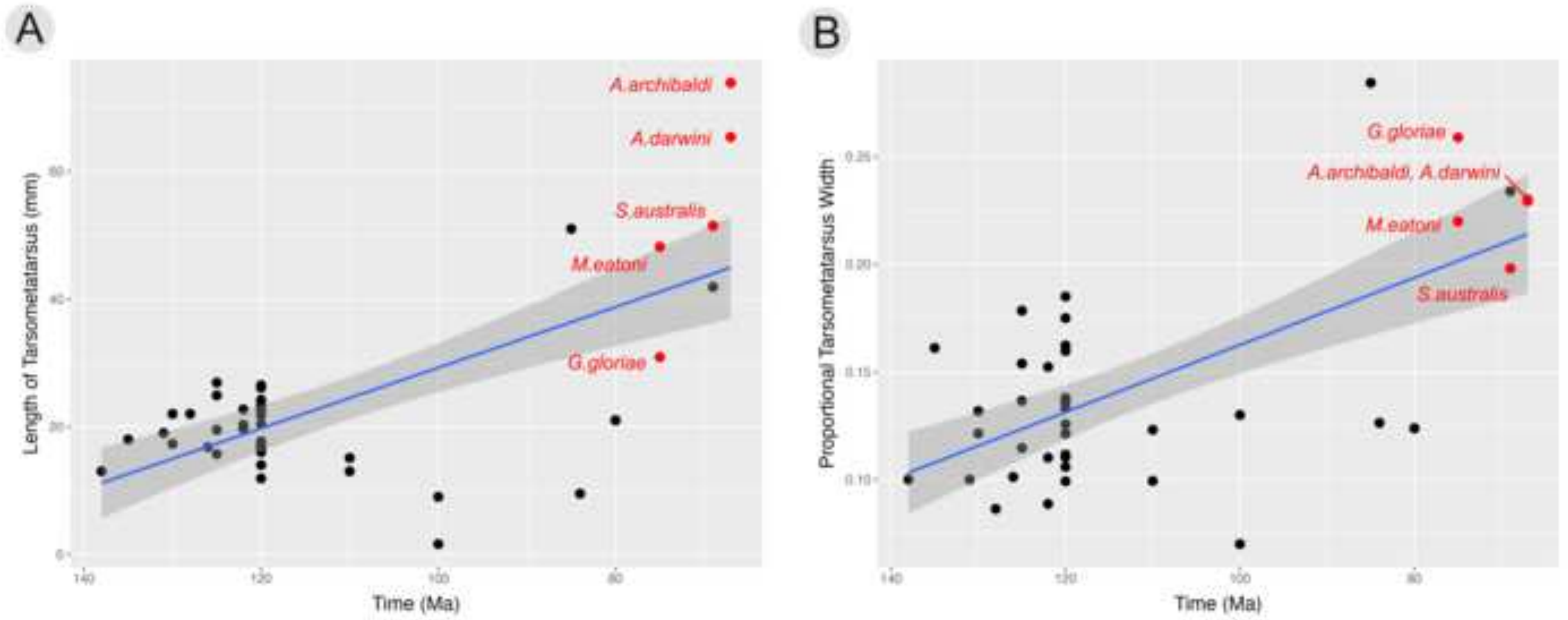

Figure 7
Click here to access/download;Figure;Figure 7.png

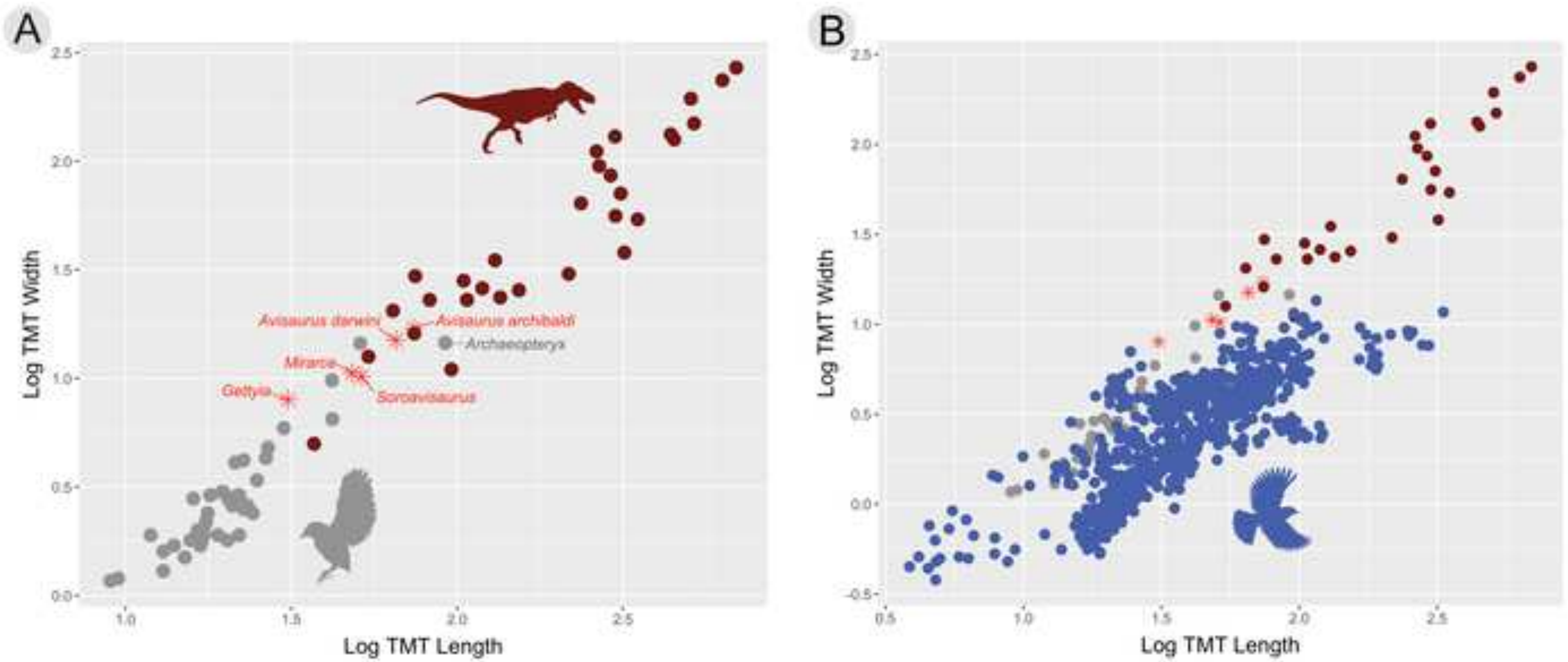

Figure 8

Click here to access/download;Figure;Figure 8.png

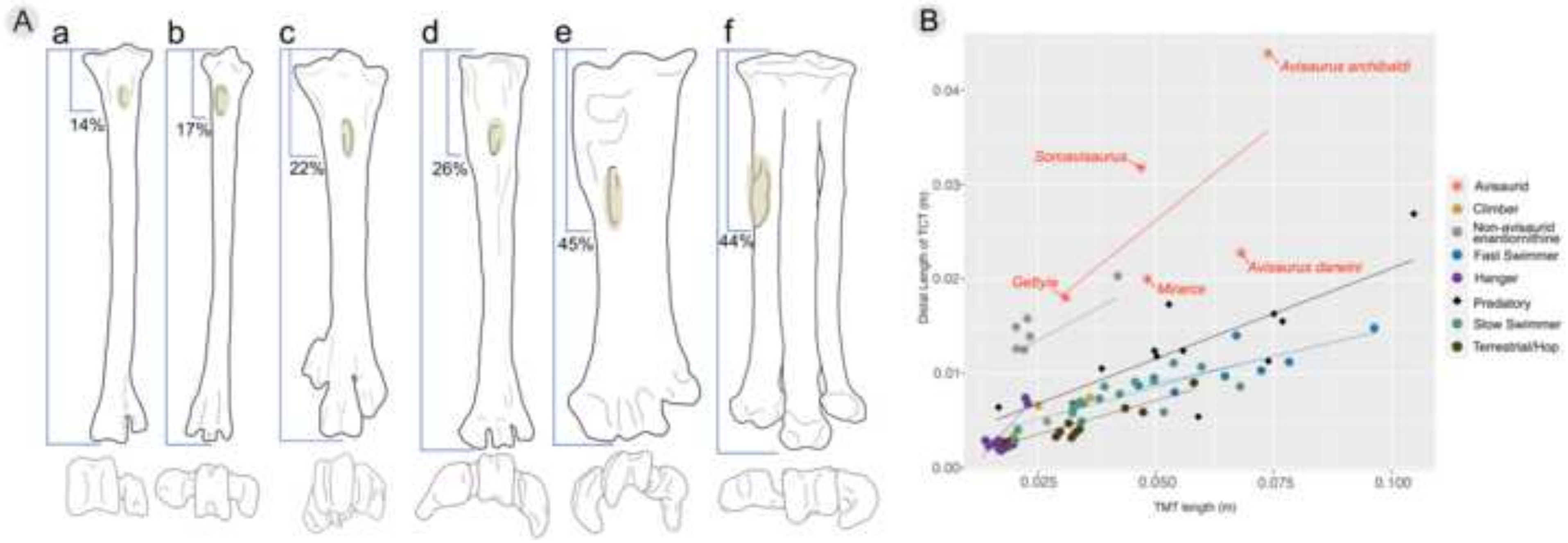

Figure 9

Click here to access/download;Figure;Figure 9.png

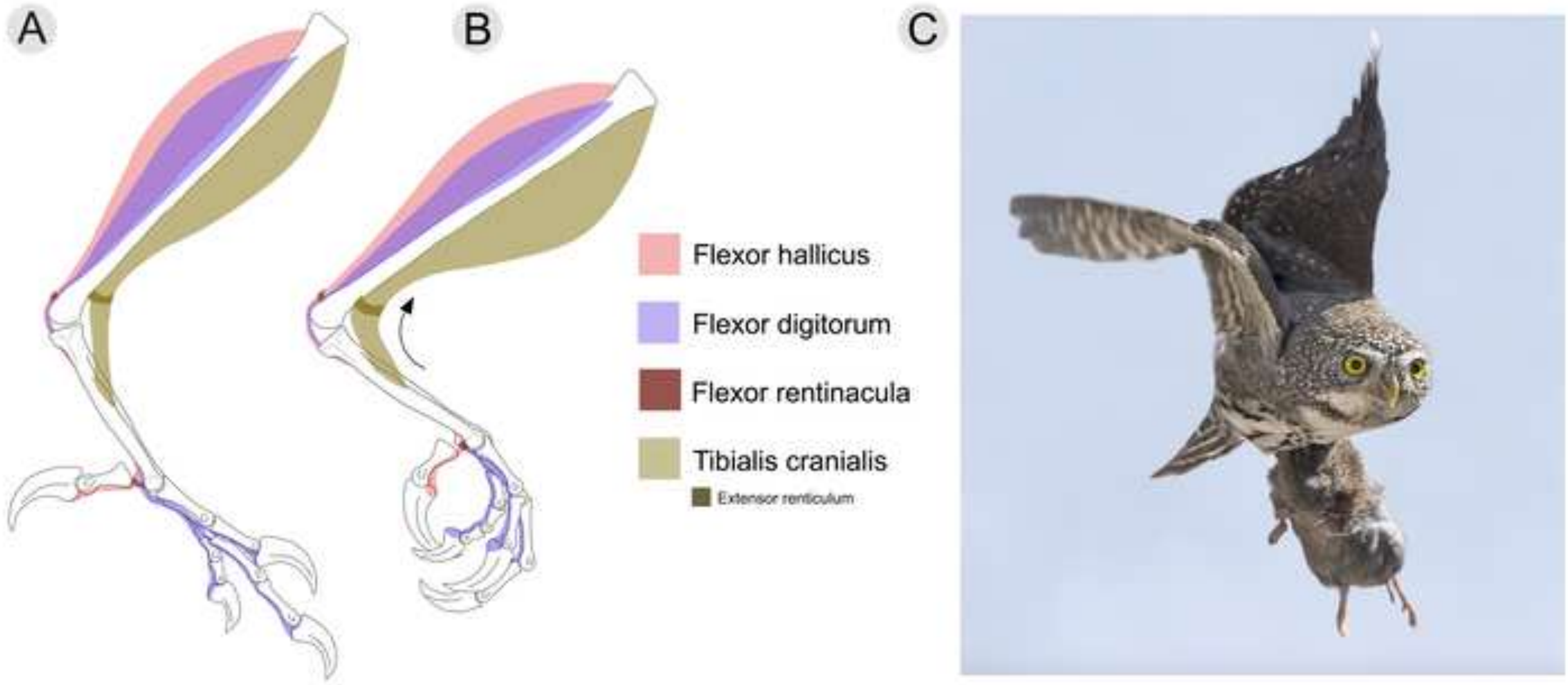

Figure 10

Click here to access/download;Figure;Figure 10.png

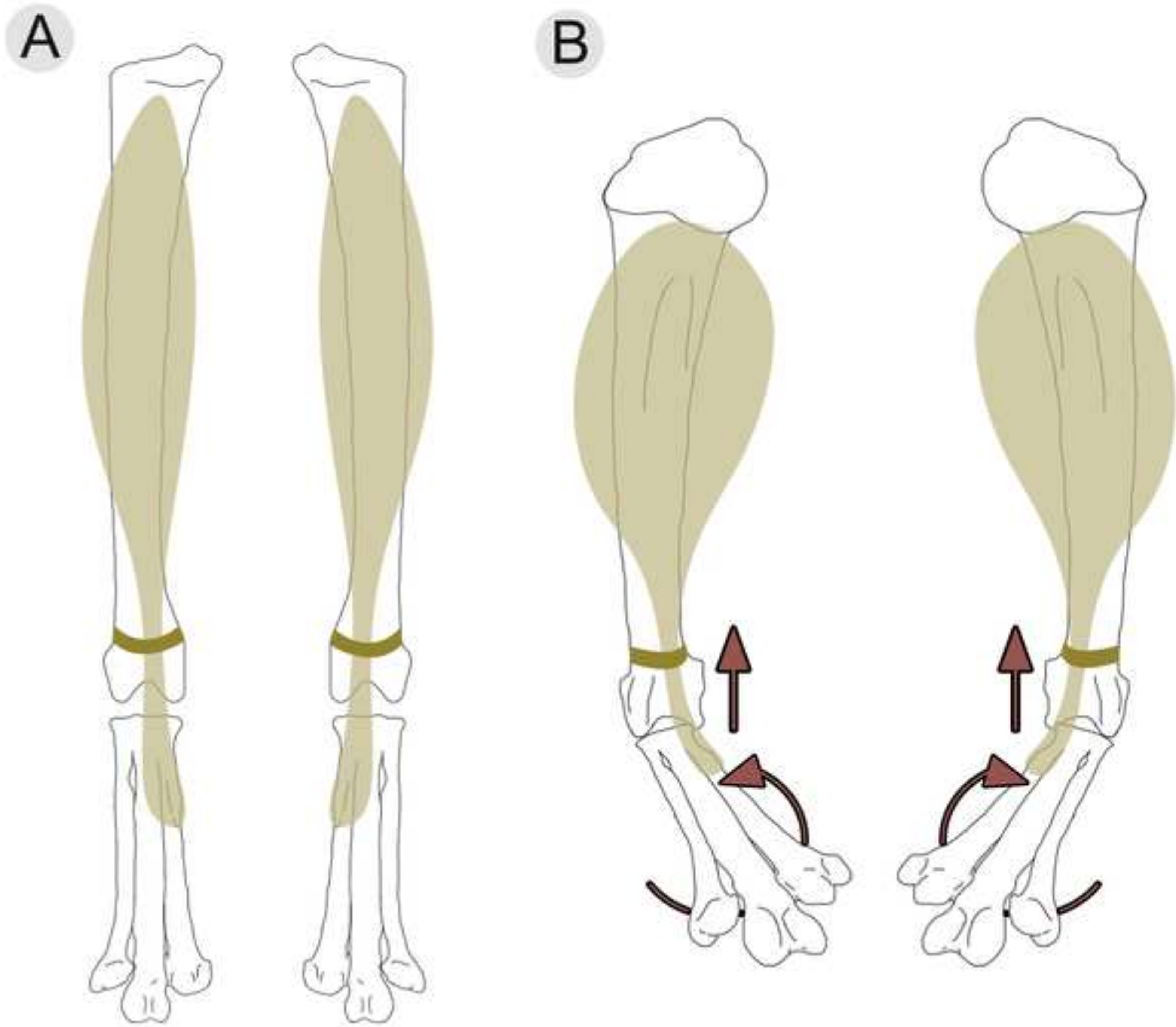

Figure 11

Click here to access/download;Figure;Figure 11.png

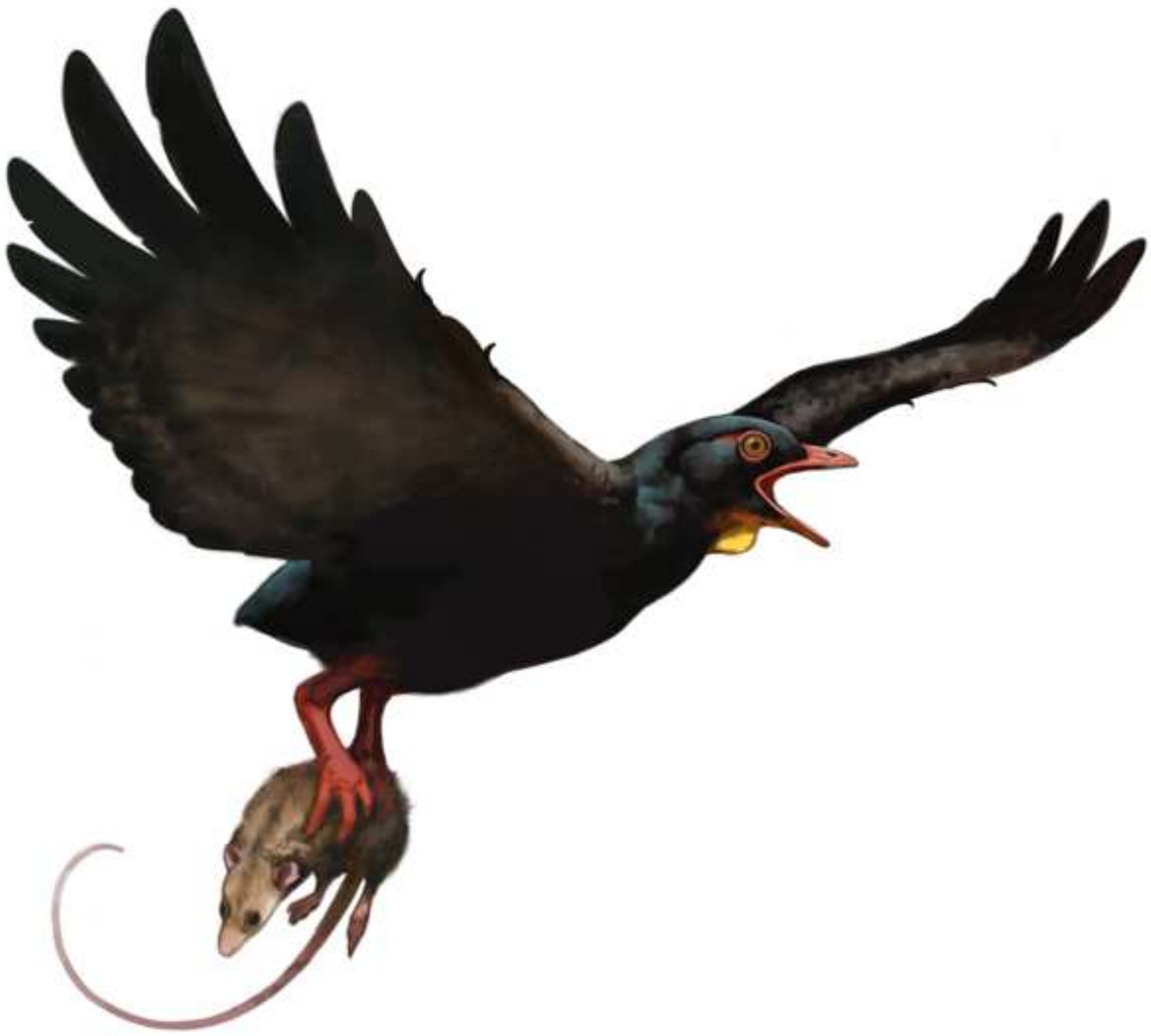

Figure S1

Click here to access/download
Supporting Information
Figure S1.png

Figure S2

Click here to access/download
Supporting Information
Figure S2.png

Code

Click here to access/download
Supporting Information
AvisaurusTMT_Rcode (for journal).R

Character Matrix

Click here to access/download
Supporting Information
Phylogenetic Characters.docx

Character Matrix Grading

Click here to access/download
Supporting Information
Grading_file.txt
