# Supplementary material for: New enantiornithine diversity in the Hell Creek Formation and the functional morphology of the avisaurid tarsometatarsus
Source: PLoS One. 2024 Oct 9;19(10):e0310686. doi: 10.1371/journal.pone.0310686 (PMC11463745; doi:10.1371/journal.pone.0310686)
Supplement: Attachment — Submitted filename: PONE-D-24-29019_ED_notes.pdf [file pone.0310686.s010.pdf]

PLOS ONE

New enantiornithine diversity in the Hell Creek Formation and the functional morphology of the avisaurid tarsometatarsus

--Manuscript Draft--

**Manuscript Number:**	PONE-D-24-29019R1
**Article Type:**	Research Article
**Full Title:**	New enantiornithine diversity in the Hell Creek Formation and the functional morphology of the avisaurid tarsometatarsus
**Short Title:**	New Hell Creek enantiornithines and avisaurid tarsometatarsi functional morphology
**Corresponding Author:**	Alexander Clark, M.S.<br>University of Chicago Division of the Biological Sciences<br>Chicago, UNITED STATES OF AMERICA
**Keywords:**	Enantiornithes, paleobiology, Hell Creek Formation, paleoecology, Mesozoic birds
**Abstract:**	Enantiornithines were the most diverse group of birds during the Cretaceous, comprising over half of all known species from this period. The fossil record and subsequently our knowledge of this clade is heavily skewed by the wealth of material from Lower Cretaceous deposits in China. In contrast, specimens from Upper Cretaceous deposits are rare and typically fragmentary, yet critical for understanding the extinction of this clade across the K-Pg boundary. The most complete North American Late Cretaceous enantiornithine is Mirarce eatoni, a member of the diverse clade Avisauridae. Except for Mirarce, avisaurids are known only from isolated hindlimb elements from North and South America. Here we describe three new enantiornithines from the Maastrichtian Hell Creek Formation, two of which represent new avisaurid taxa. These materials represent a substantial increase in the known diversity of Enantiornithes in the latest Cretaceous. Re-examination of material referred to Avisauridae through phylogenetic analysis provides strong support for a more exclusive Avisauridae consisting of six taxa. Exploration of the functional morphology of the avisaurid tarsometatarsus indicates potential strong constriction and raptorial attributes. The lower aspect ratio of the tarsometatarsus facilitates a more biomechanically efficient lever system which in extant birds of prey equates to lifting proportionally heavier prey items. In addition, the proportional size and distal position of the m. tibialis cranialis tubercle of the tarsometatarsus is similar to the morphology seen in extant birds of prey. Together with the deeply-grooved metatarsal trochlea facilitating robust and likely powerful pedal digits, morphologies of the hindlimb suggest avisaurids as Late Cretaceous birds of prey.
**Order of Authors:**	Alexander Clark, M.S.
	Jessie Atterholt
	John Scanella
	Thomas Carr
	Nathan Carrol
	Jingmai O'Connor, PhD
**Opposed Reviewers:**	Michael Pittman, PhD<br>The Chinese University of Hong Kong<br><br>Previous unethical dilemmas with coauthors
	Juan Benito<br>Cambridge University: University of Cambridge<br><br>Previous unethical dilemmas with coauthors
**Response to Reviewers:**	Editor<br><br>1. Please ensure that your manuscript meets PLOS ONE's style requirements,

Powered by Editorial Manager® and ProduXion Manager® from Aries Systems Corporation

including those for file naming. The PLOS ONE style templates can be found at https://journals.plos.org/plosone/s/file?id=wjVg/PLOSOne_formatting_sample_main_body.pdf and https://journals.plos.org/plosone/s/file?id=ba62/PLOSOne_formatting_sample_title_authors_affiliations.pdf
Completed
2. In your manuscript, please provide additional information regarding the specimens used in your study. Ensure that you have reported human remain specimen numbers and complete repository information, including museum name and geographic location.
If permits were required, please ensure that you have provided details for all permits that were obtained, including the full name of the issuing authority, and add the following statement:
'All necessary permits were obtained for the described study, which complied with all relevant regulations.'
If no permits were required, please include the following statement:
'No permits were required for the described study, which complied with all relevant regulations.'
For more information on PLOS ONE's requirements for paleontology and archeology research, see https://journals.plos.org/plosone/s/submission-guidelines#loc-paleontology-and-archaeology-research.
Completed
3. Please take this opportunity to be sure you have met all of our guidelines for new species. For proper registration of a new zoological taxon, we require two specific statements to be included in your manuscript.
1) In the Results section, the globally unique identifier (GUID), currently in the form of a Life Science Identifier (LSID), should be listed under the new species name, for example:

Anochetus boltoni Fisher sp. nov. urn:lsid:zoobank.org:act:B6C072CF-1CA6-40C7-8396-534E91EF7FBB
Another LSID for the manuscript itself should also appear within the Nomenclature statement. You will need to contact Zoobank (zoobank.org/About) to obtain a GUID (LSID). You should receive one LSID for your manuscript and a separate, unique LSID for the new species.
Completed
2) Please also insert the following text into the Methods section, in a sub-section to be called ""Nomenclatural Acts"":
Completed
The electronic edition of this article conforms to the requirements of the amended International Code of Zoological Nomenclature, and hence the new names contained herein are available under that Code from the electronic edition of this article. This published work and the nomenclatural acts it contains have been registered in ZooBank, the online registration system for the ICZN. The ZooBank LSIDs (Life Science Identifiers) can be resolved and the associated information viewed through any standard web browser by appending the LSID to the prefix ""http://zoobank.org/"". The LSID for this publication is: urn:lsid:zoobank.org:pub: XXXXXXX.
The electronic edition of this work was published in a journal with an ISSN, and has been archived and is available from the following digital repositories: PubMed Central, LOCKSS [author to insert any additional repositories].
Completed
All PLOS ONE articles are deposited in PubMed Central and LOCKSS. If your institute, or those of your co-authors, has its own repository, we recommend that you also deposit the published online article there and include the name in your article.
Following a recent ruling by the International Commission on Zoological Nomenclature, electronic journals are now a valid format for publication of new zoological taxa. In order to ensure the valid publication of your new species, please be sure to include the updated version of Nomenclatural Acts (above).
A complete explanation of our guidelines for publishing new species can be found on our website: http://www.plosone.org/static/guidelines#zoological.
4. When completing the data availability statement of the submission form, you indicated that you will make your data available on acceptance. We strongly recommend all authors decide on a data sharing plan before acceptance, as the process can be lengthy and hold up publication timelines. Please note that, though access restrictions are acceptable now, your entire data will need to be made freely

accessible if your manuscript is accepted for publication. This policy applies to all data except where public deposition would breach compliance with the protocol approved by your research ethics board. If you are unable to adhere to our open data policy, please kindly revise your statement to explain your reasoning and we will seek the editor's input on an exemption. Please be assured that, once you have provided your new statement, the assessment of your exemption will not hold up the peer review process.
Completed
5. Please amend either the title on the online submission form (via Edit Submission) or the title in the manuscript so that they are identical.
Completed
6. We are unable to open your Supporting Information file [AvisaurusTMT_Rcode (for journal).R]. Please kindly revise as necessary and re-upload.
Completed
7. Please review your reference list to ensure that it is complete and correct. If you have cited papers that have been retracted, please include the rationale for doing so in the manuscript text, or remove these references and replace them with relevant current references. Any changes to the reference list should be mentioned in the rebuttal letter that accompanies your revised manuscript. If you need to cite a retracted article, indicate the article's retracted status in the References list and also include a citation and full reference for the retraction notice.
Completed

Reviewer #1: Clark et al. describe three new specimens of avisaurids birds, and use them to support the existing hypothesis that avisaurids were raptorial birds. Attached to my review is a PDF of the manuscript with comments made directly.

The comments made throughout the manuscript by the reviewer were both helpful and productive, and we thank the reviewer for their advice and assistance in creating a stronger manuscript. We found the section concerning the mass ranges of birds particularly helpful and constructive. We have made sure that our wording is clearer when referring to a particular group or when referring to the higher percentile range of that particular group [i.e., we have made it clear when referring to a portion of a family ("larger members") or to the family as a whole]. We have also included better and more specific sources for where size ranges are sourced from.

All comments or suggestions made by the reviewer which required a simple fix or alteration have been corrected throughout the manuscript and figures. There were a number of points brought up within the manuscript notes that we will address here. Lastly, we have also revised all figures as requested including figure 6, 8, 11 and included a new supplemental figure with better images of the proximoplantar fossa as requested (Fig. S2).

Comparison with penguins: We removed the brief section in the discussion that covered comparisons with penguins as requested. Originally, we wanted to include a brief section comparing this group as we felt that including them made sense given the superficially similar aspect ratios. However, as commented on, the manuscript does not require this section and it has since been removed to make the discussion more streamline.

Pengornathids as late-diverging enantiornthines: Thank you for pointing this phylogenetic result out! Our results do differ from those in Atterholt et al., (2018), but pengornithids are recovered as late-diverging enantiornithines in other published literature as well:
O'Connor et al., 2013 - 10.1080/02724634.2012.719176
Wang et al., 2022 - 10.1017/jpa.2022.12
Li et al., 2021 - 10.1111/joa.13588
O'Conner et al., 2020 - 10.1038/s41586-020-2945-x

Introduction comment: We find that the section within the introduction which discusses previous criteria for the Avisauridae is needed both for the reader to understand the history of avisaurids, and for the introduction to be a proper review of previous literature. For readers with limited knowledge of this clade of enantiornithines, we find that introducing these topics (discussing prior synapomorphies) within the introduction

to be important and relevant. By discussing these taxa within the introduction, we feel that we have given the reader a thorough and relevant summary of the current state of this clade of enantiornithines. Additionally, we briefly cover why there are initial issues with previous criteria for admittance to this clade. With this section in the introduction, it allows the reader to see that prior criteria for consideration into the Avisauridae requires an update – which is something we state as an objective in the introduction, and also why some taxa are not considered to be avisaurids here.

Figure 6: With regards to additional work for figure 6 (regression analysis): We have made sure to include both slope and resulting R2 values for both A and B in figure 6 (and for figure S1 were requested). However, we find that phylogenetically influenced results when dealing with enantiornithines can lead to further confusion given that the phylogeny of this entire clade is still highly debated. Our objective in this figure is to only direct the reader to the fact that over time, as far as we can tell from the fossil record, enantiornithine body size remains fairly conserved early in their evolution and expands to both lower and higher thresholds by the end Cretaceous.

Line 545 (relating to lowering aspect ratio equating to greater efficiency) “This is untrue. Chiefly, a lower aspect ratio does not inherently affect the relative length of the tmt. Aspect ratio chiefly describes the relative length and width within the tmt, but not its muscle tendon insertion location nor its length relative to any other bone in the hindlimb. If an accipitrid had its tmt midshaft stretched laterally its aspect ratio would reduce, but the tmt would still be just as long and have the same fulcrum, moment of action, and resistance locations.”

In birds, we typically do see that lower aspect ratio tarsometatarsi do change the proportional lengths against the tibiotarsus. Additionally, when the aspect ratio diminishes, particularly in birds of prey, we also see an increase in the proportional size (i.e., surface area) of the tubercle. In the hypothetical example given by the reviewer (stretching a hawk’s tarsometatarsus), we would expect, based on all other birds of prey, that the relative position and surface area of the tubercle would also change, creating a whole different lever system. If the tarsometatarsus was stretched mediolaterally, it would alter how the hindlimb works and in reality, other parts of the system would change. To the point, we see this very thing happen but in reverse in the Barn owl (Tyto alba). Barn owls have essentially taken the owl-like tarsometatarsus and increased the aspect ratio, elongating it. The result is not little change to the lever system, but alternatively, it has taken the form of a much more hawk-like tarsometatarsus, resulting in an altogether different lever system than other strigiforms. In this relevant example, by changing the aspect ratio, you change the relative length compared to the tibiotarsus, and the position of the tubercle. These results are actually forthcoming in another manuscript soon to be submitted focused on accipitrid and strigid tarsometatarsi.
We find that changes in aspect ratios do in fact change relative location of the tubercle and the lever system as a whole. Lastly, by lowering the aspect ratio, you inherently reduce the proportional length against the other hindlimb bones among birds. Aspect ratio does not scale proportionally among birds of prey – which is made evident in owls and hawks assuming different methods of prey acquisition. Again, when a member of either order evolves aspect ratios closer to the other order, it then exhibits prey acquisition strategies closer to the latter.

Comment: “Perhaps the most obvious association with a higher aspect ratio goes unmentioned, though: Higher resistance to bending forces. As elongate bones thicken in a dimension perpendicular to their long axis they tends to better resist bending forces (see e.g. Cuff and Rayfield). So a tmt with a relatively low aspect ratio will be relatively better able to resist forces like those in locomotion and hunting”.
Thank you for bringing this point up. This thought did occur to us during our initial research, however, a key feature that differs between neornithines and enantiornithines kept this point from our discussion. In neornithines rigidity of the foot is far better facilitated as they have fully fused metatarsals while enantiornithines do not. Even robust avisaurids still retain the enantiornithine feature of fusion limited to the proximal portion. Torsion of the individual metatarsals would be more likely for enantiornithines compared to extant birds of prey today. However, as the reviewer states, this point is debatable. However, we feel it would not contribute enough to be included in our discussion due to the high level of uncertainty regarding rigidity in

unfused metatarsals.

Owl vs hawk comments: There appeared to a few points of misunderstanding with the reviewer that we believe were both addressed in the original draft and are further clarified in the revised manuscript. Particularly with the comments addressing lines 539 and 545 (above), we felt that we made it clear that avisaurids did not function either as owls or as accipitrids in their given environment, but they do display morphological similarities to both groups, suggesting a unique morphological solution to perhaps a similar predatory niche. We lean more so into strigids throughout our discussion because they show more similarities with the lower aspect ratio of the tarsometatarsus and the subsequent lever system implications. However, we have made sure throughout the revised manuscript to state that we are drawing from strigid morphology, not stating it as a direct analogue for how avisaurids functioned. We have more our discussion points, including the ability to lift proportionally heavy things, clearer and address limitations of increasing size. Thank you for making this clearer.

Line 553. "These are not just driven by the low aspect ratio of the tmt, though. The cranial face of owl tarsometatarsi have deep excavations that run down nearly half the length for additional adductor muscle attachment area, and the plantar side is even more dramatically incised to accommodate the large flexor muscles. While the low aspect ratio of the avisaurid tmt does allow for relatively broader muscle attachments than other enantiornithines, it seems unlikely to me that gripping abilities analagous to owls can be attributed to Avisauridae."
We agree with the reviewer; however, we do not state that avisaurids had the gripping abilities as owls – only that they were likely increased relative to other enantiornithines (which we have clarified in the text). We have made sure to include words such as "analogous" to strigid grip where requested in the manuscript. The objective of this portion of the discussion was to signal that owls have morphologies that allow for increased grip relative to accipitrids. In the same way, avisaurids show similar, uniquely-strigid reminiscent morphologies.

Fig. 9 comment: "This is likely more an effect of body size than musculature. Lift scales with wing area and muscle cross-sectional area, both of which scale to body mass^2/3. As such, larger raptorial birds tend to either hunt relatively less massive prey or to kill prey on the ground without lifting it. The Northern pygmy owl in the figure has an average mass of around 70 g, less than a tenth of mass estimates for A. darwini. So feats like this would be unlikely for the avisaurid."
We agree with the reviewer. Assuredly, a bird the size of a large avisaurid could not lift items nearly three times its own weight (similar to much smaller strigids). However, the biomechanics for increased compensation for carrying weight are present. We have made sure to make this clear in the figure's description and the discussion/conclusion sections.

Line 689: "Pedal phalanges are known from Mirarce. Do they have similar aspect ratios to owls'? To other raptorial birds?"
This will be discussed in a forthcoming manuscript! Thank you for pointing it out. For this manuscript we wanted to remain fixed on morphology and function of the tarsometatarsus (which includes the deeply grooved trochlea).

The descriptive portions of the manuscript are well-executed. Morphology is detailed and comparisons to establish new species are appropriate. I have requested some more detail on the Methods, but many of these requests could easily be met with a supplemental file.
Thank you again for your constructive comments throughout the manuscript. I am not sure why the supplemental tables were unavailable to you, but we have made sure to upload them again in csv format this time so that the R code will read them correctly.

However, there are some issues in the authors' evidence presented for avisaurids as raptorial birds. In particular, the authors place much emphasis on the similarity between avisaurids and owls. In doing so, they ignore several major differences between owls and avisaurids and cite several similarities also shared with other raptorial birds. The authors mention briefly in the Abstract and Conclusion that avisaurids likely inhabited a raptorial niche not reminiscent of any extant group, and I believe this hypothesis has more support than using owls as analogues and should be

more fleshed-out in the body of the manuscript.
(see above comment). Thank you again for pressing on this central point of our manuscript. We have made sure to clarify our points both as directed and where needed.

Finally, most of the underlying data for this study has not been provided. None of the supplemental tables nor the .csv files which the uploaded R code relies on were a part of the submission (at least not a part I could access).
(see above comment). We apologize for this issue. All files were previosuly uploaded correctly. However, we have reuploaded all supplemental files in the correct way according to the PLOS one submission system. Additionally, we have changed all excel files to .csv and altered the Rcode to better read these files. We hope that all uploads function correctly during the next revision. Thank you.

Overall, I believe this work is publishable with relatively minor revisions (including all supplemental files, more detailed Methods, removing commented inaccuracies from Discussion and lessening emphasis on owls as full analogues) but can be made much more impactful with more major revisions (building a more cohesive argument around Avisauridae’s unique raptorial niche).
Thank you very much again for your suggestions and constructive points.

The authors' work is both interesting and well-executed and would be an excellent addition to PLOS ONE.

Reviewer #2: The manuscript is in good shape and ready for publication. I have only a few comments:

1- I wouldn't call "Williams Quarry" a Lagerstatte. There is no evidence of soft tissue preserved or any type of other exceptional type of preservation. I believe WQ is more appropriately referred to as a 'bonebed'. You may want to also cite a recent paper on a basicranium from WQ as this paper refers to the discovery of over 1000 enantiornithine bones at this quarry (Chiappe LM, et al 2022. Proc. R. Soc. B 289: 20221398. https://doi.org/10.1098/rspb.2022.1398)
Thank you for the correction. This has been revised in the manuscript to read as “a productive bonebed” rather than a Lagerstatte. The new reference has also been cited.

2. The passage in lines 446-448 is confusing. The authors say that "Currently, the smallest Upper Cretaceous
enantiornithines are Burmese amber specimens (Elektorornis, approximately the size of 3 g extant
trochilids) and the largest is the holotype of Pengornis houi [similar in size to large psittaciforms (600-
448 750 grams) (Zhou et al., 2008)]." However Pengornis is an early Cretaceous taxon--please revise this section, and note that Chiappe (among others) highlighted the fact that Avisaurids are relatively large birds. This is stated on several papers, including those cited in this papers.
The amber specimens are younger than 100.5 My, making them Upper Cretaceous specimens.

3. The discussion about a trend of increased body size during the evolutionary history of enantiornithines needs a bit more of historical context, as the authors--based on the references they cite--make it look as this has only been noticed recently. In fact, almost 30 years ago (e.g. Chiappe 1996) this trend was clearly noticed together with the fact that the entire spectrum of body size increased significantly towards the Late Cretaceous. As an example, I'm including a section from Chiappe & Walker (2002), a paper already cited by the authors:

"During their evolution throughout the Cretaceous there is a general increment in size and size range among the eu- enantiornithine species. All known Early Cretaceous eu-enantiornithines are small to medium-size birds (see com- parative measurements in Bochenski, 1996). Sinornis and Eoalulavis, for example, were the size of a sparrow or finch, and the size of Nanantius and Concornis was comparable to that of a medium-sized thrush. Late Cretaceous euenan- tiornithines, however, are generally larger: Gobipteryx was the size of a jackdaw and Neuquenornis that of a small fal- con. Some

	of the Late Cretaceous taxa were much larger. Enantiornis had a wingspan of about 1.2 meters, ranging in size between a skua and a turkey vulture (Chiappe, 1996b), and a recently discovered enantiornithine from France has been compared in size to a herring gull (Buffetaut, 1998). Some Late Cretaceous euenantiornithines, however, retained the small size of their Early Cretaceous relatives; Alexornis was sparrow-sized." Corrected. We have made sure to acknowledge that this trend has been previously recognized. Thank you for the correction
**Additional Information:**	
**Question**	**Response**
**Financial Disclosure**<br><br>Enter a financial disclosure statement that describes the sources of funding for the work included in this submission. Review the submission guidelines for detailed requirements. View published research articles from *PLOS ONE* for specific examples.<br><br>This statement is required for submission and **will appear in the published article** if the submission is accepted. Please make sure it is accurate.<br><br>**Funded studies**<br>Enter a statement with the following details:<br>• Initials of the authors who received each award<br>• Grant numbers awarded to each author<br>• The full name of each funder<br>• URL of each funder website<br>• Did the sponsors or funders play any role in the study design, data collection and analysis, decision to publish, or preparation of the manuscript?<br><br>Did you receive funding for this work?	The author(s) received no specific funding for this work.
**Competing Interests**<br><br>Use the instructions below to enter a competing interest statement for this submission. On behalf of all authors, disclose any competing interests that could be perceived to bias this work—acknowledging all financial support and any other relevant financial or non-financial competing interests.	The authors have declared that no competing interests exist.

This statement is required for submission and **will appear in the published article** if the submission is accepted. Please make sure it is accurate and that any funding sources listed in your Funding Information later in the submission form are also declared in your Financial Disclosure statement.<br><br>View published research articles from *PLOS ONE* for specific examples.<br><br>**NO authors have competing interests**<br><br>Enter: *The authors have declared that no competing interests exist.*<br><br>**Authors with competing interests**<br><br>Enter competing interest details beginning with this statement:<br><br>*I have read the journal's policy and the authors of this manuscript have the following competing interests: [insert competing interests here]*<br><br>* typeset	
**Ethics Statement**<br><br>Enter an ethics statement for this submission. This statement is required if the study involved:<br><br>• Human participants<br>• Human specimens or tissue<br>• Vertebrate animals or cephalopods<br>• Vertebrate embryos or tissues<br>• Field research<br><br>Write "N/A" if the submission does not require an ethics statement.<br><br>General guidance is provided below. Consult the submission guidelines for detailed instructions. **Make sure that all information entered here is included in the Methods section of the manuscript.**	N/A

**Format for specific study types**<br><br>**Human Subject Research (involving human participants and/or tissue)**<br>• Give the name of the institutional review board or ethics committee that approved the study<br>• Include the approval number and/or a statement indicating approval of this research<br>• Indicate the form of consent obtained (written/oral) or the reason that consent was not obtained (e.g. the data were analyzed anonymously)<br><br>**Animal Research (involving vertebrate animals, embryos or tissues)**<br>• Provide the name of the Institutional Animal Care and Use Committee (IACUC) or other relevant ethics board that reviewed the study protocol, and indicate whether they approved this research or granted a formal waiver of ethical approval<br>• Include an approval number if one was obtained<br>• If the study involved *non-human primates*, add *additional details* about animal welfare and steps taken to ameliorate suffering<br>• If anesthesia, euthanasia, or any kind of animal sacrifice is part of the study, include briefly which substances and/or methods were applied<br><br>**Field Research**<br><br>Include the following details if this study involves the collection of plant, animal, or other materials from a natural setting:<br>• Field permit number<br>• Name of the institution or relevant body that granted permission	
**Data Availability**<br><br>Authors are required to make all data underlying the findings described fully available, without restriction, and from the time of publication. PLOS allows rare exceptions to address legal and ethical concerns. See the PLOS Data Policy and FAQ for detailed information.	Yes - all data are fully available without restriction

Powered by Editorial Manager® and ProduXion Manager® from Aries Systems Corporation

A Data Availability Statement describing where the data can be found is required at submission. Your answers to this question constitute the Data Availability Statement and **will be published in the article**, if accepted.<br><br>**Important:** Stating 'data available on request from the author' is not sufficient. If your data are only available upon request, select 'No' for the first question and explain your exceptional situation in the text box.<br><br>Do the authors confirm that all data underlying the findings described in their manuscript are fully available without restriction?	
**Describe where the data may be found in full sentences. If you are copying our sample text, replace any instances of XXX with the appropriate details.**<br><br>• If the data are **held or will be held in a public repository**, include URLs, accession numbers or DOIs. If this information will only be available after acceptance, indicate this by ticking the box below. For example: *All XXX files are available from the XXX database (accession number(s) XXX, XXX.).*<br>• If the data are all contained **within the manuscript and/or Supporting Information files**, enter the following: *All relevant data are within the manuscript and its Supporting Information files.*<br>• If neither of these applies but you are able to provide **details of access elsewhere**, with or without limitations, please do so. For example:<br><br>*Data cannot be shared publicly because of [XXX]. Data are available from the XXX Institutional Data Access / Ethics Committee (contact via XXX) for researchers who meet the criteria for access to confidential data.*<br><br>*The data underlying the results presented in the study are available*	All data used for analyses is present within either in-text tables or supplementary tables. Additionally, coding has been included that produced our discussed quantitative results.

from (include the name of the third party and contact information or URL).<br>• This text is appropriate if the data are owned by a third party and authors do not have permission to share the data.<br><br>* typeset	
Additional data availability information:	Tick here if the URLs/accession numbers/DOIs will be available only after acceptance of the manuscript for publication so that we can ensure their inclusion before publication.

Powered by Editorial Manager® and ProduXion Manager® from Aries Systems Corporation

Greetings,

I am writing on behalf of myself and my colleagues to submit an article manuscript titled "New enantiornithine diversity in the Hell Creek and the functional morphology of the avisaurid tarsometatarsus" for consideration to be published in PLOS One. Enantiornithines were the most diverse clade of birds during the Mesozoic. Currently, enantiornithine literature is heavily skewed to Lower Cretaceous deposits of China, making Late Cretaceous North American specimens incredibly valuable concerning the state of this speciose clade near the end Cretaceous event. Within our manuscript we describe three new Late Cretaceous North American enantiornithine taxa which expand the known body size diversity of this speciose clade and hint at unique raptorial ecologies. Importantly with these three new taxa, the maximum body size among clade members reached previously unseen maximums. Of these sizeable, Late Cretaceous enantiornithines, the largest members belong to the family Avisauridae. Though avisaurid specimens are often fragmentary, their three-dimensional preservation, robust morphology, and unique osteological correlates allow for examination of the functional morphology of the hindlimb. Specifically, the tarsometatarsus among members of this family exhibit striking similarities to extant birds of prey such as accipitrids (e.g., hawks, eagles), and strigids (owls). Our manuscript presents multiple anatomically-supported lines of evidence to support our conclusions which suggest avisaurids occupied a raptorial niche that lacks one-to-one comparisons among extant taxa. Assessment of the tarsometatarsus suggests a novel muscular arrangement and a functional morphology that unites features that are independently exhibited among extant birds of prey. Overall, our manuscript adds to the diversity of this already speciose clade, describes important Upper Cretaceous specimens, and applies comparative anatomical approaches to better understand the functional morphology of these fascinating birds.

Thank you very much for your consideration.

Sincerely,

Alexander D. Clark
(Corresponding and Lead Author)

Manuscript Click here to access/download;Manuscript;Manuscript.docx

Full title: New enantiornithine diversity in the Hell Creek Formation and the functional morphology of the avisaurid tarsometatarsus

Short title: New Hell Creek enantiornithines and avisaurid tarsometatarsi functional morphology

Alexander D. Clark[1,2*], Jessie Atterholt[3], John B. Scannella[4], Thomas D. Carr[5], Nathan Carroll[6], Jingmai K. O'Connor[2]

[1]Committee on Evolutionary Biology, University of Chicago, Chicago, IL 60637 USA
[2]Negaunee Integrative Resource Center, Field Museum of Natural History, 1400 S Dusable Lake Shore Drive, Chicago, IL. 60605 USA
[3]College of Osteopathic Medicine of the Pacific, Western University of Health Sciences, Pomona, CA, USA
[4]Museum of the Rockies and Department of Earth Sciences, Montana State University, 600 W. Kagy Blvd, Bozeman, Montana, 59717, USA
[5]Department of Biology, Carthage College, Kenosha, WI 53140, USA
[6]Carter County Museum, 306 N Main St. Ekalaka, MT, 59324 USA

Corresponding author: Alexander D. Clark, adclark@uchicago.edu

Abstract

Enantiornithines were the most diverse group of birds during the Cretaceous, comprising over half of all known species from this period. The fossil record and subsequently our knowledge of this clade is heavily skewed by the wealth of material from Lower Cretaceous deposits in China. In contrast, specimens from Upper Cretaceous deposits are rare and typically fragmentary, yet critical for understanding the extinction of this clade across the K-Pg boundary. The most complete North American Late Cretaceous enantiornithine is *Mirarce eatoni*, a member of the diverse clade Avisauridae. Except for *Mirarce*, avisaurids are known only from isolated hindlimb elements from North and South America. Here we describe three new enantiornithines from the Maastrichtian Hell Creek Formation, two of which represent new avisaurid taxa. These materials represent a substantial increase in the known diversity of Enantiornithes in the latest Cretaceous. Re-examination of material referred to Avisauridae through phylogenetic analysis provides strong support for a more exclusive Avisauridae consisting of six taxa. Exploration of the functional morphology of the avisaurid tarsometatarsus indicates potential strong constriction and raptorial attributes. The lower aspect ratio of the tarsometatarsus facilitates a more biomechanically efficient lever system which in extant birds of prey equates to lifting proportionally heavier prey items. In addition, the proportional size and distal position of the *m. tibialis cranialis* tubercle of the tarsometatarsus is similar to the morphology seen in extant birds of prey. Together with the deeply-grooved metatarsal trochlea facilitating robust and likely powerful pedal digits, morphologies of the hindlimb suggest avisaurids as Late Cretaceous birds of prey.

Keywords: Enantiornithes, paleobiology, Hell Creek Formation, paleoecology, Mesozoic birds

Introduction

Our understanding of Mesozoic avian diversity and evolution is limited by available material, increasing with each new described taxon. Several Lower Cretaceous Lagerstätte have yielded abundant fossils which provide insights to the diversity, ecology, and taxonomy of Mesozoic birds relatively early in their evolution (Zhou and Zhang, 2006). However, information from Upper Cretaceous deposits is far more limited, with the only possible productive bonebed being a newly emerging Brazilian site dubbed "William's Quarry" (Chiappe et al., 2022; Wu et al., 2021). Late Cretaceous fossil birds are critical for understanding one of the most important aspects of avian evolution: the selectivity of the end Cretaceous Mass Extinction in which only neornithines survived.

The most speciose clade of Mesozoic birds, constituting over half of all described species, are the enantiornithines (Chiappe and Walker, 2002; O'Connor et al., 2011; Walker, 1981). Members of this clade exhibit morphological diversity and are found on every continent except for Antarctica and continental Africa (Chiappe and Walker, 2002; O'Connor and Chiappe, 2011; Walker, 1981, Zhou et al., 2008). The vast majority of known specimens and described taxa come from Hauterivian-Aptian Lower Cretaceous deposits in Spain and China. As a result, our knowledge of this clade is heavily biased to a narrow time interval of approximately 10 million years (Buscalioni and Poyato-ariza, 2016; Fregenal-Martínez et al., 2017; Yu et al., 2021; Zhong et al., 2021; Zhou and Zhang, 2006).

Enantiornithine fossils from the Late Cretaceous are far more limited but critical for understanding the extinction of this once successful lineage. The only widely recognized clade from Upper Cretaceous deposits is the Avisauridae, known exclusively from North and South American deposits 86-66 Ma. Six genera have been named in the literature: *Avisaurus* (Brett-Surman & Paul, 1985), *Soroavisaurus* (Chiappe, 1993), *Neuquenornis* (Chiappe & Calvo, 1994), *Intiornis* (Novas et al., 2010), *Mirarce* (Atterholt et al., 2018), and *Gettyia* (formerly *A. gloria)* (Atterholt et al., 2018; Varrichio and Chiappe, 1995). Members of this group are mostly described from fragmentary remains, with the tarsometatarsus being the only commonly recovered element and thus critical for recognizing members of this clade. Here, we reevaluate the taxa assigned to the Avisauridae using cladistic analysis and in doing so, remove *Intiornis* and *Neuquenornis* from this group.

Previously, three synapomorphies were hypothesized to unite Avisauridae: a strongly convex dorsal margin of metatarsal III; the plantar projection of the medial condyle of metatarsal III; and a J-shaped laterally compressed metatarsal I (Chiappe and Calvo, 1994). One cladistic analysis suggested this clade also included *Enantiophoenix, Halimornis* and *Concornis* (Cau and Arduini, 2008). However, support for this hypothesis is weak. The tarsometatarsus is unknown in *Halimornis,* and thus referral to the Avisauridae is ambiguous. Similarly, the tarsometatarsus of *Enantiophoenix* is poorly preserved, making the assignment to the Avisauridae based on the convexity of the dorsal surface equivocal. Convexity of the dorsal margin of metatarsal III appears to be more widespread than previously thought, being present in non-avisaurid enantiornithines (e.g., *Imparavis, Feitianius*) (O'Connor et al., 2016; Wang et al., 2024). Additionally, as in *Enantiophoenix*, the lack of convexity in other enantiornithines may be taphonomic.

Assignment to the Avisauridae for *Concornis* was also based on the convexity of the dorsal margin of metatarsal III, and stoutness of the first phalanx of pedal digit I (Cau and Arduini, 2008). In this study, *Halimornis* and *Concornis* were regarded as distinct lineages within the Avisauridae, although with little morphological support (Cau and Arduini, 2008).

The taxon from which this family derives its name, *Avisaurus archibaldi*, is a large enantiornithine, with a tarsometatarsal length (~ 75 mm) comparable to large extant larids (e.g., *Larus marinus*, *L. glaucescens*) and accipitrids (e.g., *Buteo jamaicensis*, *Accipiter gentilis*) (Brett-Surman and Paul, 1985; Winkler et al., 2020a,b). This is in stark contrast to nearly all Early Cretaceous enantiornithines, whose average tarsometatarsal length varies between extant emberizids and turdids, tending not to exceed 25 mm in length (Chiappe and Walker, 2002) (Table S1). Avisaurids not only add to the morphological diversity of enantiornithines, but also contribute considerably to our understanding of the apparent trend of increasing body size diversity in the Late Cretaceous (Atterholt et al., 2018; Brett-Surman and Paul, 1985; Chiappe 1996; Chiappe and Walker, 2002).

Here we describe three new enantiornithine specimens, all of which come from the Hell Creek Formation (Latest Cretaceous 68-66 Ma) (Fowler, 2020). One of these new taxa is considered a new

species within the genus *Avisaurus* and another is assigned to *Avisaurus* sp.. Previously, *A. archibaldi* was the only avisaurid and enantiornithine described from these deposits (Brett-Surman and Paul, 1985). Like previously described North American avisaurids except for *Mirarce*, the new taxa are all represented by isolated tarsometatarsi. Although the material is limited, they possess morphological differences from previously known Late Cretaceous enantiornithines. Here, we revise morphological and phylogenetic diagnoses for the Avisauridae and discuss the significance of these new specimens with regards to their phylogenetic position, implications for size diversity among enantiornithines, and the functional morphology of the avisaurid tarsometatarsus.

INSTITUTIONAL ABBREVIATIONS – DDM, Dinosaur Discovery Museum Kenosha, Wisconsin, United States; MOR, Museum of the Rockies Bozeman, Montana, United States; CCM, Carter County Museum Ekalaka, Montana, United States.

Methods

All necessary permits were obtained for the described study, which complied with all relevant regulations. Specimens were photographed under normal lighting conditions with a Canon 70D dslr camera with a Canon EF 100mm f/2.8 macro lens. Photos of each specimen were assessed in Adobe Photoshop version 24.3.0 and if needed, had aspects of contrast, and color balance edited. Anatomical nomenclature primarily follows Baumel and Witmer (1993) using the English equivalents of the Latin terminology. All measurements used for description and comparisons were recorded in-person using digital calipers (OriginCal1P54). Additional measurements were recorded with ImageJ (Schneider et al., 2012). Aspect ratios of tarsometatarsi were calculated by dividing the mediolateral width at the proximodistal midpoint by the total proximodistal length at the mediolateral midpoint of the proximal-most margin to the distal-most margin of the proximodistally longest metatarsal.

Size estimates for three avisaurids (*Mirarce*, DDM 1577.730, *A. archibaldi*) were calculated to better compare body sizes to other enantiornithines and extant birds. Even though the tarsometatarsus scales with size in extant birds (Fig. S1), Field et al. (2013) found the greatest predictive power (highest R^2 scores) for determining the mass of birds was the maximum diameter of the glenoid facet of the coracoid (maximum diameter of the coracoid's humeral articulation facet – HAF) and the length of the humerus, both which can be measured in the avisaurid *Mirarce*. To estimate the mass of the two larger avisaurids known only from tarsometatarsi (DDM 1577.730 and *A. archibaldi*) the diameter of the coracoid glenoid and the length of the humerus were approximated by scaling them from *Mirarce* by using a bone which all three taxa preserve: the tarsometatarsus. The tarsometatarsus of DDM 1577.730 and *A. archibaldi* measure 140% and 155% that of *Mirarce* respectively. Using these percentages, we scaled the diameter of the coracoid glenoid and the length of the humerus in *Mirarce* to estimated lengths for DDM 1577.730 and *A. archibaldi* to allow for mass predictions. For all three avisaurids, we averaged the masses of the nearest ten extant species according to data collected by Field et al., (2013) using both the diameter of the coracoid glenoid and the length of the humerus.

Nomenclatural Acts

The electronic edition of this article conforms to the requirements of the amended International Code of Zoological Nomenclature, and hence the new names contained herein are available under that Code from the electronic edition of this article. This published work and the nomenclatural acts it contains have been registered in ZooBank, the online registration system for the ICZN. The ZooBank LSIDs (Life Science Identifiers) can be resolved and the associated information viewed through any standard web browser by appending the LSID to the prefix ""http://zoobank.org/"".

The LSID for this publication is: urn:lsid:zoobank.org:pub:53881EA9-CD01-4EAD-9339-ED912FE5C952

The electronic edition of this work was published in a journal with an ISSN, and has been archived and is available from the following digital repositories: PubMed Central, LOCKSS

Systematic Paleontology

Aves Linnaeus, 1758

Pygostylia Chiappe, 2002

Ornithothoraces Chiappe, 1995

Enantiornithes Walker, 1981

Avisauridae Brett-Surman and Paul, 1985

Diagnosis: Enantiornithines characterized by the combination of a robust tarsometatarsus (mediolateral width at the midpoint at least 20% that of the total proximodistal length); a mediolaterally broad trochlea of metatarsal II relative to III; the presence of a hypertrophied tubercle for the attachment of the *m. tibialis cranialis* on metatarsal II; the *m. tibialis cranialis* tubercle located at least 20% down the length of the tarsometatarsus from the proximal margin; and a well-developed plantar projection of the medial margin of the trochlea of metatarsal III (modified from Atterholt et al., 2018).

Included genera: *Avisaurus*– UCMP 117600, Hell Creek Formation, Late Cretaceous, Maatstrichtian, Garfield County, Montana (Brett-Surman and Paul, 1985); *Soroavisaurus*– PVL 4690, Lecho Formation, Late Cretaceous, Maastrichtian, Salta Province, Argentina (Chiappe, 1993); *Mirarce*– UCMP 139500, Kaiparowits Formation, Late Cretaceous, Campanian, Grand Staircase-Escalante National Monument, Utah (Atterholt et al., 2018); *Gettyia*– MOR 553E/1.19.91.64, Two Medicine Formation Late Cretaceous, Campanian, Glacier County, Montana (Atterholt et al., 2018).

Avisaurus Brett-Surman and Paul, 1985

Diagnosis: Avisaurid enantiornithines characterized by the following combination of characters: distal extent of metatarsal II approximately equal to that of metatarsal IV; the proximoplantar surface excavated by a pair of small fossae located where the medial and lateral margins of metatarsal III meet the plantar labrum, hereafter referred to as the proximoplantar fossae (*nomen novum*) (modified from Chiappe, 1993) (Fig. S2).

Included species: *Avisaurus archibaldi* – UCMP 117600, Late Cretaceous, Maatstrichtian, Garfield County, Montana (Brett-Surman and Paul, 1985); *Avisaurus darwini* – DDM 1577.730, Hell Creek Formation, Late Cretaceous, Maastrichtian, Carter County, Montana (this publication).

Avisaurus darwini sp. nov. (Fig. 1, Table 1)

Holotype: DDM 1577.730 is a complete right tarsometatarsus collected in July of 2022

Etymology: The specific name "*darwini*" is in honor of Charles Darwin, whose momentous research and publications helped define the field of evolutionary biology. *Avisaurus darwini*, Darwin's bird lizard.

Type horizon and locality: Hell Creek Formation, Latest Cretaceous, Maastrichtian (approximately 66 Ma). DDM 1577.730 was collected in Carter County, Montana at a locality under the auspices of the Bureau of Land management. The specimen was collected from the Madison Shark Spine locality (DDM-18-234,1N 55E 11) within a grey sand multitaxic microvertebrate site.

FIGURE 1. The left tarsometatarsus of *Avisaurus darwini* (DDM 1577.730) in A) dorsal, B) plantar, C) right lateral, D) left lateral, E) proximal, and F) distal aspects. Abbreviations: dlp, distal lateral projection; dmp, distal medial projection; dvf, distal vascular foramen; fo, fossa (collateral ligament fossa); is, intermetatarsalian space; lc, lateral cotyle; lf, laterally-projecting flange; mc, medial cotyle; mtII, metatarsal II; mtIII, metatarsal III; mtIV, metatarsal IV; pf, proximoplantar fossae; pl, proximal labrum; pp, plantar projection; tct, *m. tibialis cranialis* tubercle. Scale bar 10mm. Line illustrations done by Samantha Clark.

	Avisaurus darwini	***MOR 3070***	***Magnusavis ekalakaensis***
**Proximal Surface**			
Width	20.24	18.56	-
Height	11.65	11.15	-
**Cranial Aspect**			
MT II Length	62.93	*58.68	-
MT II Width (midpoint)	4.91	-	3.12
MTIII Length	65.38	-	-
MT III Width (midpoint)	6	-	4.8
MT IV Length	60.4	-	-
MT IV Width (midpoint)	4.98	-	-
**Distal Aspect**			
MT II Trochlea Width	8.07	-	7.38
MT II Trochlea Height	5.22	-	5.78
MT III Trochlea Width	8.18	-	8.27
MT III Trochlea Height	6.62	-	5.87
MT IV Trochlea Width	9.48	-	-
MT IV Trochlea Height	4.64	-	-
**Aspect ratio of Tmt**	1:4	*1:4	-

TABLE 1. Measurements (in mm) of each newly described specimen. For MOR 3070, asterisks represent approximations. Total length of metatarsal II was estimated by scaling MOR 3070 to *A. darwini*, which is nearly the same size.

Diagnosis –A large accipitrid-sized bird identified as an avisaurid based on the strong plantar projection of the medial condyle of the trochlea of metatarsal III; a robust tarsometatarsus (mediolateral width at the midpoint greater than 20% that of the total proximodistal length); a mediolaterally broad trochlea of metatarsal II relative to III; the presence of a hypertrophied *m. tibialis cranialis* tubercle on metatarsal II located ~ 30% down the tarsometatarsus. DDM 1577.730 is referable to the genus *Avisaurus* based on the following features: the distal extent of metatarsal II approximately equal to that of metatarsal IV; the presence of proximoplantar fossae; a metatarsal II exhibiting a laterally-projecting flange of bone from the plantar margin. DDM 1577.730 can be differentiated from other avisaurids based on the following unique combination of features: a proximally located intermetatarsal space between metatarsals III and IV which is proximodistally proportionally shorter than that of *Soroavisaurus*; metatarsal II's laterally-projecting flange of bone closes the intertrochlear incisure, contacting the distomedial margin of the metatarsal III trochlea (this feature is more pronounced than that in the lateroplantarly-projecting flange in *A. archibaldi*); both medial and lateral margins of the trochlea of metatarsal III terminate distally at the same level, disparate from *Soroavisaurus* (lateral margin extending distally) and *A. archibaldi* (medial margin extending distally).

Avisaurus sp. (Fig. 2, Table 1)

Material: MOR 3070 is a partial right tarsometatarsus preserving most of metatarsal II (distal portion disarticulated), < 50% of metatarsal III, and the proximal portion of metatarsal IV. Some additional disarticulated fragments are probably referable to the specimen but lack diagnostic morphologies.

Locality: Hell Creek Formation, Latest Cretaceous, Maastrichtian (approximately 68 - 66 Ma). MOR locality no. HC-720 "Daigo's Site". MOR 3070 was discovered by Daigo Yamamura in Makoshika State Park, Glendive, Montana and collected by a field crew led by Frankie Jackson of Montana State University (Yamamura, 2013).

FIGURE 2. The left partial tarsometatarsus of *Avisaurus* sp. MOR3070 in A) dorsal, B) plantar, and C) proximal aspects. The distal portion of metatarsal II is placed in the approximate place if it was articulated. Abbreviations: dr, dorsal ridge: lc, lateral cotyle; lf, laterally-projecting flange; mc, medial cotyle; mtII, metatarsal II; mtIII, metatarsal III; mtIV, metatarsal IV; pf, proximoplantar fossae; pl, proximal labrum; tct, *m. tibialis cranialis* tubercle. Scale bar 10mm. Line illustrations done by Samantha Clark.

Comment – A large accipitrid-sized enantiornithine with plantar-projecting medial and lateral plantar crests; dorsal face of metatarsal II is mediolaterally wider than metatarsal III proximally; the lateral cotyle elevated slightly proximally relative to the medial cotyle. MOR 3070 is referable to the genus *Avisaurus* based on the two proximoplantar fossae in plantar aspect, and a metatarsal II trochlea exhibiting a lateroplantarly-projecting flange of bone from the plantar margin. MOR 3070 differs from other closely related enantiornithines (avisaurids) based on the following unique combination of features: lateral to medial tapering of the proximal labrum's dorsal margin; a dorsally-projecting *m. tibialis cranialis* tubercle rather than dorsomedially projection as in *A. archibaldi* and *A. darwini*; metatarsal II mediolaterally greater than that of III in dorsal aspect along its proximal preserved portion; a well-developed dorsally-projecting ridge on the dorsal face of metatarsal III beginning prominently below the labrum shelf and continuing distally down the dorsal surface of the remaining portions of metatarsal III (forming a triangular cross section in plantar aspect); plantar margin of the trochlea of metatarsal II with a lateroplantarly-projecting flange, similar to *A. archibaldi* but disparate from the lateral-projecting morphology in *A. darwini*.

Aves Linnaeus, 1758

Pygostylia Chiappe, 2002

Ornithothoraces Chiappe, 1995

Enantiornithes Walker, 1981

Magnusavis ekalakaenis gen. et sp. nov. (Fig. 3, Table 1)

Holotype: CCM V2019.5.1 is a partial right tarsometatarsus, missing the proximal portions of metatarsals II and II, and all of metatarsal IV. A single phalanx is preserved, though digit assignment is uncertain.

Etymology: In Latin, "*Magnus*" meaning big, and "*avis*" meaning bird, and "*ekalakaensis*" in honor of the town of Ekalaka, Montana, close to where this specimen was discovered. Ekalaka is Lakota for "one who wanders". *Magnusavis ekalakaenis*, Ekalaka's big bird.

Locality: Hell Creek Formation, Late Cretaceous, Maastrichtian (approximately 66 Ma). The specimen was collected in Carter County, Montana at a locality under the auspices of the Bureau of Land management (Permit MTM 108846).

FIGURE 3. The partial left tarsometatarsus of *Magnusavis ekalakaensis* (CCM V2019.5.1) in A) dorsal, B) plantar, and C) distal aspects, D) and the single preserved pedal phalanx in dorsal and right lateral aspects. Abbreviations; mtII, metatarsal II; mtIII, metatarsal III; pp, proximal projection; psf, plantar supratrochanter fossa; tct, *m. tibialis cranialis* tubercle. Scale bar 10mm. Line illustrations done by Samantha Clark.

Diagnosis – A larid-sized enantiornithine differentiated from other taxa, including the closely-related avisaurids, based on the following unique combination of features: distal expansion of metatarsals II and III (all avisaurids retain nearly equal medial and lateral margins of the metatarsals down their lengths); a medial and lateral-rimmed (i.e., well-excavated) plantar supratrochanter fossa on metatarsal III; and an articular face on metatarsal III (for metatarsal IV) terminating distal to that in *A. archibaldi*.

Phylogenetic Analysis

These three new specimens were added to a modified version of the character matrix by Atterholt et al. (2018). We added three new characters to assess the relative robustness of the tarsometatarsus [Character 253], presence of the proximoplantar fossae bordering metatarsal III [Character 254], and the presence of the intermetatarsal space between metatarsals III and IV proximal to the midpoint of the tarsometatarsus [Character 255]. The final matrix, consisting of 45 taxa with scores across 255 characters, was analyzed in TNT (Goloboff et al., 2008). For scoring, see Supplementary Information. Neornithes were represented by *Anas* and *Gallus*, and Dromaeosauridae was used as the outgroup. We conducted a heuristic search retaining the single shortest tree out of every 1000 trees followed by a second round of tree-bisection reconnection (TBR). The first round of TBR produced 36 trees with a length of 803 steps; the second round of TBR produced 4207 trees of the same length.

Results

Morphological descriptions

DDM 1577.730 – Holotype of *Avisaurus darwini*

urn:lsid:zoobank.org:act:E5235AC4-571C-4606-90D7-C25BCDC3CF2A

The metatarsals are all fused proximally to each other and the distal tarsals. The proximal articular surface is oval in shape with the lateral cotyle slightly elevated and larger relative to the medial cotyle as in *A. archibaldi*. It is greater than the cross-section of metatarsals II-IV such that it forms a well-developed labrum that overhangs the metatarsal shafts in both dorsal and plantar aspects as in *Mirarce* and *A. archibaldi* (Atterholt et al., 2018; Brett-Surman & Paul, 1985). Like *A. archibaldi*, this is more

pronounced in plantar aspect. In plantar view, just below the labrum, the proximal-most lateral and medial portions of the metatarsal III shaft are each excavated by a small fossa, referred to as the proximoplantar fossae (Fig. 1, B). These fossae are also present, although less pronounced, in *A. archibaldi* and MOR 3070, but absent in other avisaurids.

In dorsal aspect, the metatarsals do not distally expand mediolaterally, similar to *A. archibaldi*, *Gettyia*, and *Mirarce* (Atterholt et al., 2018; Brett-Surman & Paul, 1985) (Fig. 4). As in *Soroavisaurus*, there is a large intermetatarsal space between metatarsals III and IV (Fig. 1, A&B), originally referred to as a “fenestra” Chiappe (1993). This structure may be analogous to the lateral proximal vascular foramen in extant birds which allows for anastomosis between the dorsal and plantar surfaces (Chiappe, 1993; Jadwiszczak, 2015; Schwehn et al., 2024). The mediolateral width of the space measures approximately 45% of the midpoint of metatarsal IV. In *Soroavisaurus*, this space is proximodistally longer and begins more proximally, extending from nearly 5% to 36% the length of the tarsometatarsus (Chiappe, 1993). In *Intiornis* the space extends from 12% to 30% the length of the tarsometatarsus (Novas et al., 2010).

Metatarsal III is the longest, as in all avisaurids, and most enantiornithines (Atterholt et al., 2018; Chiappe and Walker, 2002). The proximodistal length of metatarsals II and IV measures 96% and 92% that of metatarsal III respectively. Metatarsal III also exhibits the greatest mediolateral width measured at the midpoint. Metatarsals II and IV measure 82% and 83% the width of metatarsal III, respectively (Table 1). Like other avisaurids (but also some non-avisaurid enantiornithines), the dorsal surface of metatarsal III is strongly convex forming a weak ridge for most of its length. The shaft of metatarsal II is similarly convex, unlike other avisaurids where this face is comparatively flatter. The distal portion of metatarsal II and its trochlea deflects medially as in *A. archibaldi*, *Gettyia*, and *Mirarce* (Atterholt et al., 2018; Brett-Surman & Paul, 1985). The shaft of metatarsal IV is cranioplantarly compressed similar to *A. archibaldi* and *Gettyia*. The distal portion of the metatarsal gently curves laterally just proximal to the mediolaterally expanded trochlea more so than *Soroavisaurus* and *Mirarce* (Atterholt et al., 2018; Brett-Surman & Paul, 1985; Chiappe, 1993).

FIGURE 4. Tarsometatarsus size variation among the Avisauridae and close relatives. A) *Avisaurus darwini*, B) MOR3070, C) *Avisaurus archibaldi*, D) *Gettyia*, E) *Soroavisaurus*, F) *Mirarce*, G) *Intiornis*, H) *Neuquenornis*. All to scale. Scale bar 10 mm.

On the craniomedial surface of metatarsal II there is a large and rugose tubercle for the attachment of the *m. tibialis cranialis* (Fig. 1, A&C). This structure is elongate, beginning 29% of the way down metatarsal II and extending for 15% of its total length, similar to *A. archibaldi* both in size and position (Brett-Surman & Paul, 1985). Distally, there is a weakly-developed intertrochlear incisure between metatarsals II and III. The space extends for approximately 11% that of the total length of metatarsal III. The distal vascular foramen between trochlea III and IV begins 78% down the length of metatarsal III and terminates at approximately 86%. The mediolateral width of this space measures approximately 34% that of the midpoint width of metatarsal IV.

The laterally-projecting flange of the lateral trochlea of metatarsal II expands such that the trochlea along the plantar margin is wider than that of metatarsal III, unlike *Soroavisaurus*. In *A. archibaldi*, this flange is proportionally smaller, projects lateroplantarly, and does not fully close the gap between the trochlea of metatarsal II and III(Brett-Surman & Paul, 1985). In *Mirarce*, this feature is also present, but is located more distally on metatarsal II and expands distolaterally (Atterholt et al., 2018). In *A. darwini*, this distal laterally-projecting flange contacts that distomedial margin of metatarsal III, closing the gap between the trochlea of metatarsals II and III. This closure creates a condition analogous to the distal vascular foramen between the trochlea of metatarsal III and IV (Atterholt et al., 2018).

The medial and lateral condyles of the metatarsal III trochlea terminate approximately at the same level, whereas the medial margin extends farther distally in *A. archibaldi* (Brett-Surman & Paul, 1985). As in *A. archibaldi* and *Mirarce*, the medial condyle of the trochlea of metatarsal III exhibits greater plantar projection than the lateral condyle (Atterholt et al., 2018; Brett-Surman & Paul, 1985). The trochlea of both metatarsals II and III are ginglymous, and exhibit well-developed, excavated, intercondylar grooves. These well-developed grooves are also present in *A. archibaldi*, *Mirarce*, *Gettyia*,

and *Soroavisaurus* (Atterholt et al., 2018; Brett-Surman & Paul, 1985; Chiappe, 1993). The trochlea of metatarsal IV has a reduced medial condyle such that it appears as a single, plantomedially excavated condyle, similar to other enantiornithines (e.g., *A. archibaldi*, *Avimaia*, *Imparavis*, *Mirarce)* (Atterholt et al., 2018; Bailleul et al., 2019; Brett-Surman & Paul, 1985; Wang et al., 2024). In distal view, the trochlea of metatarsal IV is C-shaped (medially concave), and projects farther plantarly than even the medial condyle of the metatarsal III trochlea, as in other avisaurids (e.g., *Mirarce, A. archibaldi*) (Atterholt et al., 2018; Brett-Surman & Paul, 1985).

Specimen MOR 3070 - *Avisaurus* sp.

The metatarsals are all fused proximally to each other and the distal tarsals (Fig. 2). Similar to other *Avisaurus*, the proximal articular surface is oval with the lateral cotyle slightly elevated relative to the medial cotyle. The proximal labrum is well-developed as in *A. archibaldi*, *A. darwini*, and *Mirarce* (Atterholt et al., 2018; Brett-Surman & Paul, 1985). However, unlike *A. darwini*, and *Mirarce*, MOR 3070 exhibits greater lateral to medial tapering of the labrum thickness along the dorsal margin (Fig. 2, A; Atterholt et al., 2018). In plantar aspect, the labrum is dorsoventrally thickest at its midpoint (i.e., above the midline of metatarsal III). Characteristic of the genus *Avisaurus*, two proximoplantar fossae bordering metatarsal III are present. These fossae are not as deep as in *A. darwini*.

Though incomplete, MOR 3070 would have had a low aspect ratio of the tarsometatarsus (~ 1:4) like other avisaurids (e.g., *Mirarce*, *Gettyia*, and *Avisaurus*). Proximally, metatarsal II is the mediolaterally widest metatarsal, with the width of metatarsal III measuring approximately 87% that of the metatarsal II. This in contrast to *Mirarce, A. archibaldi*, and *A. darwini*, where even proximally the width of metatarsal II is less than that of III (Atterholt et al., 2018). Similar to members of *Avisaurus*, MOR 3070 exhibits a well-developed *m. tibialis cranialis* tubercle which if scaled to *Avisaurus* would place it approximately 30% down the length of metatarsal II. In contrast to other avisaurids, the *m. tibialis cranialis* tubercle of MOR 3070 projects more dorsally as opposed to dorsomedially. The disarticulated

distal portion of metatarsal II exhibits similarities to *A. archibaldi* in that it exhibits a lateroplantarly-projecting flange (Brett-Surman & Paul, 1985). This is disparate from the laterally-projecting morphology exhibited in *A. darwini*. The relationship of this flange with the trochlea metatarsal III is unknown, but similar to *A. archibaldi*, it likely did not contact metatarsal III (this flange contacts the trochlea of metatarsal III in *A. darwini*). Beginning prominently below the labrum shelf, a well-developed dorsally-projecting ridge extends distally down the remaining portion of the dorsal surface of metatarsal III (forming a triangular cross section in plantar aspect). This feature is absent in *A. darwini*, and weakly developed in *A. archibaldi*.

CCM V2019.5.1 – Holotype of *Magnusavis ekalakaenis*

urn:lsid:zoobank.org:act:4A5DF7A9-06FD-45A0-B7CA-726B1936D7DA

The holotype of *M. ekalakaenis* consists of partial metatarsals II and III and a single phalanx (Fig. 3). Unlike avisaurids, both preserved metatarsals exhibit subtle distal expansion. The proximal-most mediolateral width of metatarsal III measures 76% that of the distal-most portion (proximal to the trochlea) (*A. darwini*, 100%, *A. archibaldi*, 96%). Metatarsal III is both proximodistally longer and mediolaterally wider than metatarsal II along its length, typical of most enantiornithines (Chiappe and Walker, 2002).

The shaft of metatarsal II is rounded and smooth, in contrast to the well-developed peaked ridge in MOR 3070 and *A. darwini*. The distomedial deflection of metatarsal II is weakly-developed compared to the condition seen in avisaurids, exhibiting a nearly closed intertrochlear incisure between metatarsal II and III. Though not as pronounced or as large as the condition seen in avisaurids, the *m. tibialis cranialis* tubercle is well developed when compared to most enantiornithines (Atterholt et al., 2018; Brett-Surman & Paul, 1985). Similar to metatarsal II, the dorsal surface of metatarsal III is only weakly convex, in contrast to the strongly convex morphology in avisaurids (e.g., *Mirarce*, *A. archibaldi*, MOR 3070, and, *A. darwini*).

The condyles of the metatarsal III trochlea terminate at the same level distally. Like avisaurids, the intercondylar grooves are well-developed in both metatarsals II and III, particularly in III. The medial condyle of the metatarsal III trochlea exhibits greater dorsal and plantar projection relative to the lateral condyle. The plantar supratrochanter fossa is also well developed, and clearly demarcated lateral and medially. The mediolateral width of this depression measures approximately 75% the total width of the trochlea of metatarsal III. The lateral fossa of metatarsals II and III are well-developed.

Though digit assignment of the phalanx is uncertain, it resembles the first phalanx of pedal digit II in *Mirarce*, albeit much more dorsoventrally deep (lateral aspect ratio *Magnusavis* 1:3, *Mirarce* 1:6). The aspect ratio of the phalanx is approximately 1:3, in contrast to that seen in Mirarce (~ 1:5).

Mass Estimates

Based on the maximum diameter of the glenoid facet of the coracoid and the length of the humerus in *Mirarce*, we calculated an estimated mass of ~ 540 g (range 432 – 808 g) (Table S2). Similarly, the resulting estimated body mass for *A. darwini* (DDM 1577.730) was ~ 1200 g (range 914 – 1712 g) and ~ 1700 g for *A. archibaldi* (range 1488 – 2283 g) (Fig. S3, Table S2).

Phylogenetic Analysis Results

Both the Nelson strict consensus tree and the 50% majority rule tree place all three new taxa within enantiornithines, with *A. darwini* and MOR 3070 (*Avisaurus* sp.) resolved as part of the Avisauridae clade (Fig. 5).

FIGURE 5. Phylogenetic placement of new Hell Creek enantiornithines based on cladistic analysis. A) A subset of the strict consensus tree focusing on taxa around the Avisauridae, and B) the full majority tree. In the majority tree, the newly diagnosed Avisauridae family in comprised of six taxa, two of which are

described in this publication. C) A 1.8 m tall human to scale with *A. darwini* (maroon), the largest known Early Cretaceous enantiornithine *Pengornis* (black), a medium-sized early enantiornithine, *Imparavis* (white), and finally, a minuscule enantiornithine, *Elektorornis* (smaller inset black). The extant *Buteo jamaicensis* (Red-tailed Hawk) is shown in grey.

Within the Avisauridae, *A. archibaldi* and *A. darwini* form a more exclusive clade supported by a metatarsal II that is approximately equal in distal extent to metatarsal IV [Character 236 1 → 0]. *Mirarce* and *Gettyia* form a clade supported by the absence of a plantarly excavated tarsometatarsus [Character 230 1 → 0], a *m. tibialis cranialis* tubercle located near or distal to the midpoint [Character 247 1 → 2], and the absence of proximoplantar fossae [Character 254 1 → 0]. These two clades form a polytomy with MOR 3070 and *Soroavisaurus*. The Avisauridae clade formed by these six OTU (operational taxonomic units) is supported by a tarsometatarsus whose midpoint width measures at least 20% that of its length (i.e., low aspect ratio) [Character 253 1 → 0], and a metatarsal II trochlea that is broader than that of metatarsal III [Character 238 0 → 1].

Magnusavis, *Neuquenornis*, and *Intiornis* are part of a larger polytomy outside of the Avisauridae along with *Mystiornis*, *Halimornis*, *Shenqiornis*, and *Elsornis*. With the removal of two previously identified avisaurids (*Neuquenornis*, and *Intiornis*), this family is now primarily defined not by the convex dorsal surface of metatarsal III or plantar excavation of the tarsometatarsus, characters that are both widespread in enantiornithines (e.g., *Imparavis*, *Elektorornis*, *Qiliania*) (Ji et al., 2011; Xing et al., 2019; Wang et al., 2024), but instead by a proportionately robust tarsometatarsus (mediolateral width at the midpoint at least 20% that of the total proximodistal length). The removal of these two taxa differs from previous phylogenetic results which place them alongside avisaurids based on a plantar projection of the medial trochlea of metatarsal III (Atterholt et al., 2018). These results support interpretations that MOR 3070 is an avisaurid and likely a member of the genus *Avisaurus* (e.g., robust morphology, two proximoplantar fossae), and one that differs from other avisaurids; however, in light of the fragmentary remains available at present, we refrain from naming this specimen as a new taxon.

Discussion

Size Diversity in Late Cretaceous Enantiornithines

Here we describe three new large birds from the Maastrichtian Hell Creek Formation in Montana, all of which are placed in the clade Enantiornithes (Fig. 5). These three new enantiornithines not only increase the known taxonomic diversity of the Hell Creek avifauna, but they also add to existing evidence that members of this speciose clade achieved proportionally larger body sizes near the end of the Cretaceous.

Most enantiornithines described from Lower Cretaceous deposits, notably the Hauterivian to Aptian Lagerstätte in Spain and China (~ 130 – 120 Ma), and Upper Cretaceous amber specimens from Myanmar (~ 99 – 100 Ma), are similar in size to sparrows (families Passeridae, Emberizidae) within the range of ~ 10 – 70 grams and thrushes (family Turdidae) within the range of ~ 20 – 170 grams using the length of the humerus as a means of comparison (Chiappe and Walker, 2002; O'Connor et al., 2009; Tobias et al., 2022; Wang et al., 2024; Winkler et al., 2020c-e; Zhou, 2002). Currently, the smallest Upper Cretaceous enantiornithines are Burmese amber specimens (*Elektorornis*, predicted to the size of 3 - 5 g trochilids based on hindlimb measurements) and the largest being the holotype of *Pengornis houi* which is similar in size to larger members within Psittaciformes within the range of 600-750 grams (Billerman, 2020; Tobias et al., 2022; Zhou et al., 2008)]. Mass estimates for avisaurids reveal even larger Late Cretaceous enantiornithines with *Mirarce*, *A. darwini*, and *A. archibaldi* estimated as having body masses of ~ 540 g, ~ 1200 g, and ~ 1700 g respectively (see methods and results sections).

The description of two new large-bodied avisaurids (*A. darwini*, *Avisaurus sp*.), and another proportionally large enantiornithine (*Magnusavis*), provides additional evidence in support of an observed trend in enantiornithines towards greater body size diversity over time (Atterholt et al., 2018; Chiappe, 1996; Chiappe and Walker, 2002; O'Connor, 2022). This increase in body size disparity towards the end

Cretaceous is also observed in the Ornithuromorpha, the clade that includes Neornithes (Longrich et al., 2011). It has been suggested that larger body size among ornithuromorphs may have been a key contributing factor in surviving the end Cretaceous mass-extinction (Longrich et al., 2011; Torres et al., 2021). Although depositional environments yielding bird fossils in Upper Cretaceous North American deposits are biased toward the preservation of larger organisms, this does not explain the apparent absence of larger enantiornithines in Lower Cretaceous Lagerstätte like the Jehol, which records larger birds like *Jeholornis* and *Sapeornis* but only small enantiornithines (Hu et al., 2023; Zhou and Zhang, 2003). Therefore, the signal indicating increased size diversity in the Late Cretaceous is likely a genuine one (Fig. 6). Fragmentary smaller-bodied enantiornithines have been recovered from the Late Cretaceous of North America, both in the Hell Creek Formation and the William's quarry site in Brazil (Longrich et al., 2011; Wu et al., 2021).

FIGURE 6. Regression analyses of A) tarsometatarsus length against time, and B) proportional tarsometatarsus mediolateral width against time. Both result in significant ($p < 0.01$) positive associations with an increase in body size over time. Avisaurids are colored red and all other enantiornithine are colored black.

In enantiornithines, both an increase in tarsometatarsus length and proportional mediolateral width (at the midpoint) are significantly positively associated with time ($p < 0.01$) (Fig. 6). Though well-preserved enantiornithine tarsometatarsal material from 119 – 80 Ma is limited, represented by two specimens from Myanmar preserved in amber (e.g., DIP V 19354, *Elektorornis*) (Xing et al., 2019, 2020), before 119 Ma the average tarsometatarsus length was 19 mm and the proportional mediolateral thickness was 12% (~ 2.28 mm) (aspect ratio of ~ 1:8). In contrast, these averages are 43 mm and 21% (mediolateral thickness of 9 mm) (aspect ratio 1:5) after ~ 80 Ma (Fig. 6). This pattern is likely exaggerated by the poor Upper Cretaceous fossil record and the fact no postcranial material has yet been described from William's Quarry, although it is reportedly present in abundance (Wu et al., 2021).

Functional Morphology of the Avisaurid Tarsometatarsus

The Avisauridae are known almost entirely from tarsometatarsi. Members of the Avisauridae exhibit a combination of morphologies of the tarsometatarsus that are distinct from other enantiornithines and suggest raptorial behaviors including: the aspect ratio of the tarsometatarsus; the morphology and location of the *m. tibialis cranialis* tubercle; and the well-developed grooves of the trochleae. Specifically, these characteristics in avisaurids show striking similarities to strigids (owls, order Strigiformes) and accipitrids (hawks, eagles, order Accipitriformes). However, the differences between avisaurids and these extant clades indicates that neither provides a direct analogy for the ecological role occupied by avisaurids in the latest Cretaceous. The unique union of morphologies observed in avisaurids demonstrates that they likely occupied a predatorial ecological niche morphologically unrepresented in extant forms.

Aspect Ratio of the Tarsometatarsus

The morphology and topography of the tarsometatarsus greatly varies among extant birds, and can signal certain ecological behaviors such as wading, cursoriality, perching, prey grasping abilities, and pedal digit to substrate interaction (De Mendoza and Gómez, 2022; Grant, 1971; Madan et al., 2017; Tronrud, 2022; Volkov, 2004; Zeffer and Norberg, 2003a, b). When assessing the length and width of the tarsometatarsus of Mesozoic taxa, avisaurids overlap with a range of terrestrial nonvolant theropods (Fig. 7, Table S4). Thus, it is not surprising avisaurids were originally interpreted as non-avian, or as having reduced flight capabilities (Brett-Surman and Paul, 1985). However, when modern birds are added to the dataset, avisaurids cluster with larger birds like pelicans, owls, and hornbills weakening support for using the aspect ratio of the tarsometatarsus alone to predict volancy (Fig. 7).

FIGURE 7. Assessing aspect ratio of A) extinct avian and non-avian theropods, and then with the addition of B) extant volant birds. The grey dots represent extinct volant theropod genera. The red dots represent non-avian theropod genera. Blue dots represent extant avian genera. Refer to Table S4 for data.

Small bodied strigids (< 350 g) exhibit tarsometatarsi aspect ratios very similar to avisaurids (e.g., *Surnia ulula*, 1:4, *Aegolius funereus*, 1:5, *Glaucidium brasiliensis* 1:5) (Fig. 8). Similarities such as the aspect ratio of the tarsometatarsus, location of the *m. tibialis cranialis* tubercle, and the deeply notched trochlea of the metatarsal all suggest extant raptorial birds, and more specifically strigids, may be the best functional analogues for understanding hindlimb function in avisaurids. Strigids are able to produce greater grip (i.e., constriction of pedal digits) force than other similar sized birds of prey (Fowler et al., 2009; Tronrud, 2022; Ward et al., 2002). The proportionally more elongate tarsometatarsi of accipitrids (aspect ratios of 1:20 – 1:10) are better suited for rapid movements (i.e., grabbing, snatching), whereas the proportionally stouter tarsometatarsi of strigids are better equipped for forceful constriction (Tronrud, 2022; Ward et al., 2002). By lowering the aspect ratio of the tarsometatarsus relative to their tibiotarsus, strigids reduce the length between the fulcrum (ankle joint), moment of action (muscle tendon insertion), and the resistance force (the prey), therefore reducing the required muscular force (i.e., torque) to lift or grip prey compared to longer-legged accipitrids (Vogel, 2013). In combination with having more mechanically efficient legs for producing dorsiflexing torque, strigids also possess mediolaterally wider muscle tendons and proportionally larger flexor muscles (e.g., *m. flexor digitorum longus*) than similarly sized accipitrids (Fowler et al., 2009; Tronrud, 2022; Volkov, 2004; Ward et al., 2002). The lower aspect ratio of the tarsometatarsus accommodates these wide muscle tendons, resulting in more applied force (i.e., contraction) over a given area. The *m. flexor digitorum longus*, which is responsible for contracting pedal digits II-IV, is the largest observable lower hindlimb muscle in strigids; the muscle belly, located on the caudal surface tibiotarsus, is so large that it also protrudes around the medial and lateral borders of the tibiotarsus (Volkov, 2004; Ward et al., 2002). The combination of potentially mediolaterally wider tendon

insertions on the tarsometatarsus and greater force from increased flexor muscle mass in avisaurids may have resulted in comparatively more powerful and efficient pedal grip relative to other enantiornithines.

FIGURE 8. A) The left tarsometatarsi in dorsal and distal aspects with the *m. tibialis cranialis* tubercle highlighted in a) *Struthio camelus* (terrestrial ratite), b) *Turdus olivator* (arboreal passerine), c) *Cygnus buccinator* (semi-aquatic anseriform), d) *Buteo jamaicensis* (raptorial accipitrid), e) *Surnia ulula* (raptorial strigid), f) *Avisaurus darwini*. The distal view of each taxon is represented underneath the dorsal view to show variations of the intertrochlear grooves. Tarsometatarsi are not to scale. Reference photos of tarsometatarsi sourced from morphosource. B) Tarsometatarsus length (in meters) against the proportional distal location of the *m. tibialis cranialis* tubercle (TCT) in extant birds and enantiornithines, including avisaurids (marked as asterisks). Extant birds are divided into their primary means of locomotion, following data from Zeffer and Norberg (2003).

Similarly, the tarsometatarsi of avisaurids exhibit lower aspects ratios than all other enantiornithines (with the exception of *Bauxitornis*), suggesting that members within this clade had analogously powerful flexion of pedal digits II-IV (Dyke and Osi, 2010). However, strigids are zygodactyl (with digits I and IV reversed), whereas avisaurids and accipitrids are anisodactyl (only digit I reversed). In contrast to strigids, accipitrids exhibit greater developed *m. flexor hallucis longus* tendons (Volkov, 2004). This facilitates greater force to the contraction of the hallux (and partially to digit II) which is necessary as it opposes the combined forces generated by the three forward facing digits (II-IV) (Volkov, 2004; Ward et al., 2002). The mediolaterally wide and well-developed plantar excavation of the tarsometatarsus in large avisaurids (e.g., *Mirarce*, *Gettyia*, *Avisaurus*) is a space to accommodate mediolaterally expanded muscle tendons (of the *m. flexor hallucis longus* and *m. flexor digitorum longus*) as well as presence of a large *m. flexor hallucis brevis* belly (originating on the proximal part of the plantar surface), facilitating greater contractile grip. Avisaurids appear to unite the pedal arrangement of accipitrids and the mechanical benefits of the low aspect ratio tarsometatarsi in strigids.

Additionally, the well-developed, shelf-like labrum along the proximoplantar surface suggests the presence of an in-vivo cartilaginous hypotarsus, similar to that described in *Confuciusornis* (Jiang et al., 2017). If present, this feature would both aid in supporting the tendons of the digital flexor muscles and function as a moment arm for the ankle joint, allowing for greater tendon stability and ventroflexion of the lower hindlimb, respectively (Clifton and Carr, 2018; Mayr, 2016).

Functional Significance of the *M. Tibialis Cranialis* Tubercle

Present in confuciusornithiforms, ornithuromorphs, and enantiornithines, the tubercle on metatarsal II has been hypothesized to represent the insertion point for the *m. tibialis cranialis* (Chiappe and Walker, 2002). This feature is particularly pronounced (dorsally projecting) in enantiornithines, present in nearly all taxa in which preservation allows observation. Interestingly, this feature is noticeably absent in non-avian theropods, and located on metatarsal III in a select number of Mesozoic birds (e.g., *Mystiornis*, *Confuciusornis*, *Ichthyornis*) (O'Connor et al., 2014). In extant birds, the *m. tibialis cranialis* originates on the proximocranial surface of the tibiotarsus and inserts on the dorsoproximal surface of the tarsometatarsus (Moreno and Carrascal, 1993; Zeffer and Norberg, 2003). As the muscle tendon dorsodistally extends from the distal tibiotarsus to the tarsometatarsus, it is secured in place by a retinaculum on the craniodistal surface of the tibiotarsus, just proximal to the ankle joint (Moreno and Carrascal, 1993; Zeffer and Norberg, 2003). Contraction of this muscle dorsiflexes the ankle, bringing the dorsal face of the tarsometatarsus towards the cranial surface of the tibiotarsus (Moreno and Carrascal, 1993; Zeffer and Norberg, 2003). The larger the insertion point (i.e., tubercle), the larger the corresponding muscle tendon, resulting in more powerful dorsiflexion. Likewise, as the insertion point migrates distally, it increases the in-lever of the system while the out-lever remains the same, increasing the system's mechanical advantage and ultimately allowing for increased dorsiflexion while carrying weight in the pes (Gaslow, 1967; Moreno and Carrascal, 1993; Vogel, 2013) (Fig. 9).

FIGURE 9. A) Reconstruction of an articulated left tibiotarsus, tarsometatarsus, and pes of *Avisaurus darwini* with muscular reconstruction of the primary pedal flexors (caudal surface) and *m. tibialis cranialis* (cranial surface) in medial aspect. A) ankle joint shown in neutral position. B) ankle joint shown in dorsiflexion. Pedal elements are based on *Mirarce* which preserves nearly all pedal phalanges (Atterholt et al., 2018). The tibiotarsus is based on the distal portion preserved in Mirarce and the reconstruction given in Atterholt et al. (2018). Muscles such as the gastrocnemius and extensors are not included to more clearly show the two major flexor muscles and the *m. tibialis cranialis*. The tibialis cranialis muscle attaches to the hypertrophied tubercle of metatarsal II. The action of the tibialis cranialis is flexed to dorsiflex the ankle and invert the tarsometatarsus. This feature is hypertrophied in birds of prey compared to other extant birds, and is associated with an increased capacity to grasp prey with the pes. C) A member of the genus *Glaucidium* exhibiting the carrying strength that comes from having an enlarged and distally-migrated *m. tibialis cranialis* tubercle. Within this taxon, prey items may exceed three times the body mass of the owl (del Hoyo, 1999; Deshler, 2023). Photo used with permission and credited to Roy Priest.

Variation in the location of this muscle attachment among extant birds makes it a useful osteological correlate for determining interaction between substrate (or prey) and the pedes (Moreno and Carrascal, 1993; Zeffer and Norberg, 2003). Dorsiflexion is necessary in maintaining balance by lowering the center of mass when perching, which is why this feature is often more well-developed and proximally located in passerines than in terrestrial birds (Zeffer and Norberg, 2003), and similarly why this feature is well-developed in arboreal enantiornithines (Fig. 8). Extant birds that use their legs for swimming have even more prominent *m. tibialis cranialis* tubercles that are more distally located than perching birds. This facilitates dorsiflexion of the foot against the resistance of water, to subsequently extend and produce thrust (i.e., paddling). However, raptorial birds exhibit both the largest and most distally-located *m. tibialis cranialis* tubercles (Zeffer and Norberg, 2003) (Fig. 8). Similar to the relationship between

raptorial and non-raptorial birds, avisaurid *m. tibialis cranialis* tubercles differ from other enantiornithines in being larger and more distally located (Fig. 8; Table S3).

Variation in both the size and distal location of this muscle attachment among raptorial birds reflects differences in acquisition and proportional size of prey (Volkov, 2004; Zeffer and Norberg, 2003). Along with the size and distal location of the tubercle, the relative medial location on the tarsometatarsus also affects the degree of pedal inversion that occurs when the *m. tibialis cranialis* is contracted (Fig. 10). Inversion of the pes while grasping prey increases grip stability as the force being generated pushes against the opposing foot. The more medial the location of the tubercle, the greater the degree of pedal inversion (Volkov, 2004; Zeffer and Norberg, 2003). Compared to accipitrids and falconids, strigids also have the most distomedially located *m. tibialis cranialis* tubercles. In particular, smaller bodied owls, like *Glaucidium* tend to exhibit greater distomedial migration of the attachment compared to larger strigids, further aiding in supporting proportionally larger prey when in flight. For example, *Glaucidium gnoma*, though diminutive (up to ~ 80 g) will subdue and travel with prey items that often rival or exceed their own body mass (e.g., *Microtus* sp., 60 g; *Coccothraustes* sp., 60 g; *Callipepla sp.*, 160 g) (Deshler, 2023; Zeffer and Norberg 2003) (Fig. 9B).

FIGURE 10. Action of the tibialis cranialis muscle on the ankle joint, shown in a A) resting position and a B) contracted position. As the muscle contracts, not only does the ankle dorsiflex, bringing the tibiotarsus and tarsometatarsus closer together, but the tarsometatarsus and phalanges invert (i.e., elevation of the medial border of the foot).

The *m. tibialis cranialis* tubercle in avisaurids is larger and more distomedially located on the tarsometatarsus than in any Mesozoic bird, non-avian theropod, and nearly every extant bird (Brett-Surman and Paul, 1985). The presence of the large distomedially located *m. tibialis cranialis* tubercle in avisaurids, together with the lower aspect ratio of the tarsometatarsus, suggests a functional similarity with extant birds of prey that distinguishes them from other enantiornithines. Based on the numerous

shared morphologies with strigids, avisaurid tarsometatarsi were likely adapted for strong dorsiflexion and carrying proportionally large prey items while volant (Fig. 11). However, it should be noted that the largest avisaurids are estimated to be nearly twenty times larger than *G. gnoma*, (*G. gnoma* 80 g, *A. archibaldi* ~ 1700 g) which limits the proportional size of prey that could likely be lifted given that as an object's surface area increases, its volume increases at a greater rate (i.e., the square-cube law). Carrying similar proportionally-sized prey as some strigids (e.g., *Surnia*, *Glaucidium*, *Aegolius*) may have been more plausible for smaller-bodied avisaurids like *Gettyia* (Zeffer and Norberg, 200). *Gettyia* (Fig. 4D) exhibits the proportionally distal-most *m. tibialis cranialis* tubercle. It is also the smallest known avisaurid (~ 540 g), with a tarsometatarsal length that is less than half that of *A. archibaldi* (30.9 mm, *A. archibaldi* 73.9 mm) (Atterholt et al., 2018) (Fig. 8A).

FIGURE 11. Reconstruction of an avisaurid (e.g., *A. darwini*). Morphology of the tarsometatarsus suggests that these large birds engaged in raptorial behavior and could carry proportionally large prey. Illustration done by Ville Sinkkonen.

Significance of Trochlear Groove Development

Birds which rely on increased constriction of prey (e.g., birds of prey) exhibit well-developed and deeply-grooved metatarsal trochleae, which correspond to both proportionally long and robust pedal digits (Einoder and Richardson, 2007; Gianechini et al., 2020; Madan et al., 2017; Ward et al., 2002) (Fig. 8). This grooved morphology exhibits distinctly developed excavations between the medial and lateral condyles. The deeply-grooved metatarsal trochlea corresponds to phalanges with greater-projecting intercotylar crests, and excavated articular cotyla. This morphology results in a larger articular surface between the proximal phalanges and the tarsometatarsus, resulting in a restricted range of motion at the metatarsophalangeal joint when the flexor tendons contract, (i.e., gripping an object) (Madan et al., 2017; Tronrud, 2022; Usherwood et al., 2014). Pedal digits II-IV of strigids have mediolaterally wider tendons

of the *m. flexor digitorum* than in accipitrids, facilitating stronger contraction and therefore grip strength (Madan et al., 2017; Usherwood et al., 2014). Together with the low aspect ratio of the tarsometatarsus, the more robust pedal digits of strigids are suggested to also aid in constricting prey (Fowler et al., 2009; Goslow, 1967, 1972; Madan et al., 2017; Tronrud, 2022). This may explain why strigiforms have been found to apply more constrictive force than is necessary to kill their prey items (Madan et al., 2017; Usherwood et al., 2014) Well-developed (compared to other enantiornithines) trochlear grooves are present in the mediolaterally wide trochleae of metatarsals II and III of *A. archibaldi*, *A. darwini*, *Mirarce*, *Soroavisaurus*, and *Gettyia*, suggesting similarly robust and powerful pedal digits, reminiscent of those seen in extant birds of prey (Atterholt et al., 2018; Brett-Surman & Paul, 1985; Einoder and Richardson, 2007; Madan et al., 2017; Tronrud, 2022). Together with the absolute larger size of avisaurids compared to other enantiornithines, a proportionally high level of applied pedal digit pressure (i.e., constriction) is plausible for members of this clade.

Conclusion

Here we describe three new enantiornithines, two of which are new avisaurids, all from exposures of the latest Cretaceous Hell Creek Formation in Montana. Both new avisaurids are larger than the largest Early Cretaceous enantiornithine. This supports the apparent trend towards increased body mass diversity in the Late Cretaceous among enantiornithines with large-bodied forms being most heavily represented in the Late Cretaceous fossil record. By increasing body size, this unique bird lineage explored new ecospaces suggesting that enantiornithines were still diversifying near the end Cretaceous. The morphology of the avisaurid tarsometatarsus suggests some Late Cretaceous enantiornithines adopted raptorial ecologies. Avisaurid tarsometatarsi exhibit low aspect ratios, proportionally large and distomedially located *m. tibialis cranialis* tubercles, and well-developed grooved metatarsal trochleae. All of these morphologies are uniquely found in members of extant raptorial birds, specifically strigids and

accipitrids, and suggest similar powerful pedal constriction behaviors in an anisodactyl pedal formation. By combining multiple aspects of the tarsometatarsus of both extant accipitrids and strigids, avisaurids represent a group of enantiornithines which employed raptorial behaviors that lack modern morphological analogues.

Supporting Information

FIGURE S1. Analysis of 863 extant birds (317 species) measuring mass in grams against length of the tarsometatarsus. Data from Field et al., (2013).

FIGURE S2. Close-up photos of the proximoplantar fossa on the plantar face of (A) *Avisaurus darwini*, and (B) *Avisaurus* sp. MOR 3070. The depressions are indicated by the black arrows.

FIGURE S3. Plotting three avisaurids to approximate mass. Utilizing the dataset from Field et al., (2013), three avisaurids (*A. archibaldi, A. darwini*, *Mirarce*) were plotted against (A) all sampled extant birds, and then against all birds, (B) by family. Once closely associated families were identified, an averaged mass estimate could be calculated using data collected within Field et al., (2013).

TABLE S1. Sampled enantiornithine tarsometatarsi length and width at the midpoint (mm).

TABLE S2. Field et al. (2013) measurements used for weight estimations in extant birds and predictive masses for selected avisaurids based on averaged results.

TABLE S3. Dataset from Zeffer and Norberg (2003) with added enantiornithines to assess distal location of the *m. tibialis cranialis* tubercle.

TABLE S4. Dataset used for comparative aspect ratios of the tarsometatarsus between avian and non-avian theropods. All extant taxa used are from Field et al., 2013.

Acknowledgements

Thank you to the editor and to both reviewers for their constructive suggestions for our manuscript. We would like to acknowledge the field crews of Nathan Carroll (Carter County Museum), Thomas Carr (Dinosaur Discovery Museum), and Frankie Jackson (Montana State University). Without their effort, these new taxa would not have been found. Thank you to Daigo Yamamura for providing info on the collection of MOR 3070. Thank you to the Bureau of Land Management and Montana Fish, Wildlife & Parks for providing access to lands where these discoveries were made. Thank you to Samantha Clark who illustrated the tarsometatarsi in figures 1 – 3, and to Ville Sinkkonen who illustrated figure 11. Thank you to Akiko Shinya for assistance in additional preparation of the fossils.

References

Abourachid, A., Fabre, A., Cornette, R., Höfling, E., 2017. Foot shape in arboreal birds: two morphological patterns for the same pincer‐like tool. Journal of Anatomy 231, 1‐11. https://doi.org/10.1111/joa.12614

Atterholt, J., Hutchison, J.H., O'Connor, J.K., 2018. The most complete enantiornithine from North America and a phylogenetic analysis of the Avisauridae. PeerJ 6, e5910. https://doi.org/10.7717/peerj.5910

Bailleul, A.M., O’Connor, J., Zhang, S., Li, Z., Wang, Q., Lamanna, M.C., Zhu, X., Zhou, Z., 2019. An Early Cretaceous enantiornithine (Aves) preserving an unlaid egg and probable medullary bone. Nat Commun 10, 1275. https://doi.org/10.1038/s41467-019-09259-x

Baumel, J.J., Witmer , L.M., 1993. Osteologia, in: Handbook or Avian Anatomy. Nuptial Ornithological Club, Cambridge, Massachusetts.

Biewener, A.A., 2011. Muscle function in avian flight: achieving power and control. Phil. Trans. R. Soc. B 366, 1496–1506. https://doi.org/10.1098/rstb.2010.0353

Billerman, S. M. 2020. Old World Parrots (Psittaculidae), version 1.0. In Birds of the World (S. M. Billerman, B. K. Keeney, P. G. Rodewald, and T. S. Schulenberg, Editors). Cornell Lab of Ornithology, Ithaca, NY, USA. https://doi.org/10.2173/bow.psitta4.01

Brett-Surman, M.K., Paul, G.S., 1985. A new family of bird-like dinosaurs linking Laurasia and Gondwanaland. Journal of Vertebrate Paleontology 5, 133–138. https://doi.org/10.1080/02724634.1985.10011851

Buscalioni, A.D., Poyato-ariza, F.J., 2016. Las Hoyas: a unique Cretaceous ecosystem. New Mexico Museum of Natural History and Science Bulletin.

Cau, A., Arduini, P., 2008. *Enantiophoenix electrophyla* gen. et sp. nov. (Aves, Enantiornithes) from the Upper Cretaceous (Cenomanian) of Lebanon and its phylogenetic relationships. Atti Soc. it. Sci. nat. Museo civ. Stor. nat. Milano 149, 293–324.

Chiappe, L.M., Navalón, G., Martinelli, A.G., Nava, W., Field, D.J., 2022. Fossil basicranium clarifies the origin of the avian central nervous system and inner ear. Proc. R. Soc. B. 289, 20221398. https://doi.org/10.1098/rspb.2022.1398

Chiappe, L., 1993. Enantiornithine (Aves) Tarsometatarsi from the Cretaceous Lecho Formation of Northwestern Argentina. American Museum Novitates 3083.

Chiappe, L.M., 1992. Enantiornithine (Aves) tarsometatarsi and the avian affinities of the Late Cretaceous Avisauridae. Journal of Vertebrate Paleontology 12, 344–350. https://doi.org/10.1080/02724634.1992.10011464

Chiappe, L.M., Calvo, J.O., 1994. *Neuquenornis volans*, a new Late Cretaceous bird (Enantiornithes: Avisauridae) from Patagonia, Argentina. Journal of Vertebrate Paleontology 14, 230–246. https://doi.org/10.1080/02724634.1994.10011554

Chiappe, L.M., Walker, C.A., 2002. Skeletal morphology and systematics of the Cretaceous Euenantiornithines (Ornithothoraces: Enantiornithes)., in: Mesozoic Birds: Above the Heads of Dinosaurs. University of California Press, Berkley, CA, p. pp 240-267.

Clifton, G.T., Carr, J.A., Biewener, A.A., 2018. Comparative hindlimb myology of foot-propelled swimming birds. Journal of Anatomy 232, 105–123. https://doi.org/10.1111/joa.12710

del Hoyo, J., Elliot, A., Sargatal, J., Collar, N.J., 1999. Handbook of the birds of the world. Volume 5. Lynx edicions, Barcelona.

De Mendoza, R.S., Gómez, R.O., 2022. Ecomorphology of the tarsometatarsus of waterfowl (Anseriformes) based on geometric morphometrics and its application to fossils. The Anatomical Record 305, 3243–3253. https://doi.org/10.1002/ar.24891

Deshler, J. F. 2023. Northern Pygmy-Owl (Glaucidium gnoma), version 2.0. In Birds of the World (P. G. Rodewald, Editor). Cornell Lab of Ornithology, Ithaca, NY, USA. https://doi.org/10.2173/bow.nopowl.02

Dyke, G.J., Ősi, A., 2010. A review of Late Cretaceous fossil birds from Hungary. Geological Journal 45, 434–444. https://doi.org/10.1002/gj.1209

Einoder, L.D., Richardson, A.M.M., 2007. Aspects of the hindlimb morphology of some australian birds of prey: a comparative and quantitative study. The Auk 124, 773–788. https://doi.org/10.1093/auk/124.3.773

Field, D.J., Lynner, C., Brown, C., Darroch, S.A.F., 2013. Skeletal Correlates for Body Mass Estimation in Modern and Fossil Flying Birds. PLoS ONE 8, e82000. https://doi.org/10.1371/journal.pone.0082000

Fowler, D.W., Freedman, E.A., Scannella, J.B., 2009. Predatory functional morphology in raptors: interdigital variation in talon size is related to prey restraint and immobilisation technique. PLoS ONE 4, e7999. https://doi.org/10.1371/journal.pone.0007999

Fowler, D., 2020. The Hell Creek Formation, Montana: A stratigraphic review and revision based on a sequence stratigraphic approach. Geosciences, 10(11), p.435.

Fregenal-Martínez, M., Nieves Meléndez, N., Muñoz-García, M.B., Elez, J. and Horra, R.D.L., 2017. The stratigraphic record of the Late Jurassic-Early Cretaceous rifting in the Alto Tajo-Serranía de Cuenca region (Iberian Ranges, Spain).

Gianechini, F.A., Ercoli, M.D. and Díaz-Martínez, I., 2020. Differential locomotor and predatory strategies of Gondwanan and derived Laurasian dromaeosaurids (Dinosauria, Theropoda, Paraves): Inferences from morphometric and comparative anatomical studies. Journal of Anatomy, 236(5), pp.772-797.

Goslow, G.E., 1972. Adaptive Mechanisms of the Raptor Pelvic Limb. The Auk 89, 47–64. https://doi.org/10.2307/4084059

Goslow, G.E., 1967. Functional analysis of the striking mechanisms of raptorial birds. University of California Davis.

Goloboff, P.A., Farris, J.S., Nixon, K.C., 2008. TNT, a free program for phylogenetic analysis. Cladistics 24, 774–786. https://doi.org/10.1111/j.1096-0031.2008.00217.x

Grant, P.R., 1971. Variation in the tarsus length of birds in island and mainland regions. Evolution 25, 599. https://doi.org/10.2307/2406943

Höfling, E., Abourachid, A., 2021. The skin of birds' feet: Morphological adaptations of the plantar surface. Journal of Morphology 282, 88–97. https://doi.org/10.1002/jmor.21284

Hone, D.W.E., Dyke, G.J., Haden, M., Benton, M.J., 2008. Body size evolution in Mesozoic birds. J of Evolutionary Biology 21, 618–624. https://doi.org/10.1111/j.1420-9101.2007.01483.x

Hu, H., Wang, Y., Fabbri, M., O'Connor, J.K., McDonald, P.G., Wroe, S., Yin, X., Zheng, X., Zhou, Z., Benson, R.B.J., 2023. Cranial osteology and palaeobiology of the Early Cretaceous bird

Jeholornis prima (Aves: Jeholornithiformes). Zoological Journal of the Linnean Society 198, 93–112. https://doi.org/10.1093/zoolinnean/zlac089

Jadwiszczak, P., 2015. Another look at tarsometatarsi of early penguins. Polish Polar Research 36, 343–354. https://doi.org/10.1515/popore-2015-0024

Jiang, B., Zhao, T., Regnault, S., Edwards, N.P., Kohn, S.C., Li, Z., Wogelius, R.A., Benton, M.J., Hutchinson, J.R., 2017. Cellular preservation of musculoskeletal specializations in the Cretaceous bird *Confuciusornis*. Nat Commun 8, 14779. https://doi.org/10.1038/ncomms14779

Longrich, N.R., Tokaryk, T., Field, D.J., 2011. Mass extinction of birds at the Cretaceous–Paleogene (K–Pg) boundary. Proc. Natl. Acad. Sci. U.S.A. 108, 15253–15257. https://doi.org/10.1073/pnas.1110395108

Madan, M.A., Rayfield, E.J., Bright, J.A., 2017. Scaling and functional morphology in strigiform hind limbs. Sci Rep 7, 44920. https://doi.org/10.1038/srep44920

Martin, E.M., Sherratt, E., 2023. Grasping hold of functional trade-offs using the diversity of foot forms in Australian birds. Evol Ecol 37, 945–959. https://doi.org/10.1007/s10682-023-10261-5

Mayr, G., 2016. Variations in the hypotarsus morphology of birds and their evolutionary significance. Acta Zoologica 97, 196–210. https://doi.org/10.1111/azo.12117

Mohamed, R., 2019. Histomorphological study on the tongue of the duck in the Caribbean with relation to feeding habit and #8195; J Adv Vet Anim Res 74. https://doi.org/10.5455/javar.2019.f315

Moreno, E., Carrascal, L.M., 1993. Leg morphology and feeding postures in four *Parus* species: an experimental ecomorphological approach. Ecology 74, 2037–2044. https://doi.org/10.2307/1940849

Novas, F.E., Agnolín, F.L., Scanferla, C.A., 2010. New enantiornithine bird (Aves, Ornithothoraces) from the Late Cretaceous of NW Argentina. Comptes Rendus Palevol 9, 499–503. https://doi.org/10.1016/j.crpv.2010.09.005

O’Connor, J.K., Chiappe, L.M., 2011. A revision of enantiornithine (Aves: Ornithothoraces) skull morphology. Journal of Systematic Palaeontology 9, 135–157. https://doi.org/10.1080/14772019.2010.526639

O’Connor, J., 2022. Enantiornithes. Current Biology 32, R1166–R1172. https://doi.org/10.1016/j.cub.2022.07.074

O’Connor, J.K., Wang, X., Chiappe, L.M., Gao, C., Meng, Q., Cheng, X., Liu, J., 2009. Phylogenetic support for a specialized clade of Cretaceous enantiornithine birds with information from a new species. Journal of Vertebrate Paleontology 29, 188–204. https://doi.org/10.1080/02724634.2009.10010371

O’Connor, J.K., Clark. , A.D., Herrera, F., Zhou, Z., Xiaoli , W., Zheng, X., 2023. *Longipteryx* stomach contents indicate that morphology does not accurately predict diet in enantiornithines. Cincinnati, OH

O’Connor, J.K., Averianov, A.O., Zelenkov, N.V., 2014. A confuciusornithiforms from the Early Cretaceous of Siberia and a discussion of the evolution of avian hindlimb musculature. Journal of Vertebrate Paleontology 34.

O’Connor, J.K., Li, D.-Q., Lamanna, M.C., Wang, M., Harris, J.D., Atterholt, J., You, H.-L., 2016. A new Early Cretaceous enantiornithine (Aves, Ornithothoraces) from northwestern China with elaborate tail ornamentation. Journal of Vertebrate Paleontology 36, e1054035. https://doi.org/10.1080/02724634.2015.1054035 Schneider, C. A., Rasband, W. S., & Eliceiri, K. W. 2012. NIH Image to ImageJ: 25 years of image analysis. Nature Methods, 9(7), 671–675. doi:10.1038/nmeth.2089

Schwehn, R., Engelke, E., Seiler, C., Fischer, D., Seifert, H., Pfarrer, C., Fehr, M., Legler, M., 2024. Blood Vessel Topography of the Feet in Selected Species of Birds of Prey and Owls. Veterinary Sciences 11, 88. https://doi.org/10.3390/vetsci11020088

Tobias, J.A., Sheard, C., Pigot, A.L., Devenish, A.J., Yang, J., Sayol, F., Neate-Clegg, M.H., Alioravainen, N., Weeks, T.L., Barber, R.A. and Walkden, P.A., 2022. AVONET: morphological, ecological and geographical data for all birds. Ecology Letters, 25(3), pp.581-597.

Torres, C.R., Norell, M.A., Clarke, J.A., 2021. Bird neurocranial and body mass evolution across the end-Cretaceous mass extinction: The avian brain shape left other dinosaurs behind. Sci. Adv. 7, eabg7099. https://doi.org/10.1126/sciadv.abg7099

Tronrud, B., 2022. Evolution and ecomorphology of the avian foot: the morphology of the tarsometatarsus in birds and its responses to evolutionary changes in foot use. University of Oxford.

Usherwood, J.R., Sparkes, E.L., Weller, R., 2014. Leap and Strike kinetics of an acoustically "hunting" barn owl *Tyto alba*. Journal of Experimental Biology jeb.107169. https://doi.org/10.1242/jeb.107169

Varricchio, D.J., Chiappe, L.M., 1995. A new enantiornithine bird from the Upper Cretaceous two medicine formation of Montana. Journal of Vertebrate Paleontology 15, 201–204. https://doi.org/10.1080/02724634.1995.10011219

Vogel, S., 2013. Comparative biomechanics: life's physical world, Second edition. ed. Princeton University Press, Princeton.

Volkov, S.V., 2004. The hindlimb musculature of the true owls (Strigidae: Strigiformes): morphological peculiarities and general adaptations. Ornithologia 31.

Walker, C.A., 1981. New subclass of birds from the Cretaceous of South America. Nature 292, 51–53. https://doi.org/10.1038/292051a0

Wang, X., Clark, A.D., O'Connor, J.K., Zhang, X., Wang, X., Zheng, X., Zhou, Z. 2024. First edentulous enantiornithines (Aves: Ornithothoraces) from the Lower Cretaceous Jehol Avifauna. Cretaceous Research.

Ward, A.B., Weigl, P.D., Conroy, R.M., 2002. Functional morphology of raptor hindlimbs: implications for resource partitioning. The Auk 119, 1052–1063. https://doi.org/10.1093/auk/119.4.1052

Winkler, D. W., S. M. Billerman, and I. J. Lovette. 2020a. Hawks, Eagles, and Kites (Accipitridae), version 1.0. In Birds of the World (S. M. Billerman, B. K. Keeney, P. G. Rodewald, and T. S. Schulenberg, Editors). Cornell Lab of Ornithology, Ithaca, NY, USA. https://doi.org/10.2173/bow.accipi1.01

Winkler, D. W., S. M. Billerman, and I. J. Lovette. 2020b. Gulls, Terns, and Skimmers (Laridae), version 1.0. In Birds of the World (S. M. Billerman, B. K. Keeney, P. G. Rodewald, and T. S. Schulenberg, Editors). Cornell Lab of Ornithology, Ithaca, NY, USA. https://doi.org/10.2173/bow.larida1.01

Winkler, D. W., S. M. Billerman, and I. J. Lovette. 2020c. Old World Buntings (Emberizidae), version 1.0. In Birds of the World (S. M. Billerman, B. K. Keeney, P. G. Rodewald, and T. S. Schulenberg, Editors). Cornell Lab of Ornithology, Ithaca, NY, USA. https://doi.org/10.2173/bow.emberi2.01

Winkler, D. W., S. M. Billerman, and I. J. Lovette. 2020d. Old World Sparrows (Passeridae), version 1.0. In Birds of the World (S. M. Billerman, B. K. Keeney, P. G. Rodewald, and T. S. Schulenberg, Editors). Cornell Lab of Ornithology, Ithaca, NY, USA. https://doi.org/10.2173/bow.passer4.01

Winkler, D. W., S. M. Billerman, and I. J. Lovette. 2020e. Thrushes and Allies (Turdidae), version 1.0. In Birds of the World (S. M. Billerman, B. K. Keeney, P. G. Rodewald, and T. S. Schulenberg, Editors). Cornell Lab of Ornithology, Ithaca, NY, USA. https://doi.org/10.2173/bow.turdid1.01

Wu, Y.-H., Chiappe, L.M., Bottjer, D.J., Nava, W., Martinelli, A.G., 2021. Dental replacement in Mesozoic birds: evidence from newly discovered Brazilian enantiornithines. Sci Rep 11, 19349. https://doi.org/10.1038/s41598-021-98335-8

Xing, L., O’Connor, J.K., Chiappe, L.M., McKellar, R.C., Carroll, N., Hu, H., Bai, M., Lei, F., 2019. A new enantiornithine bird with unusual pedal proportions found in amber. Current Biology 29, 2396-2401.e2. https://doi.org/10.1016/j.cub.2019.05.077

Xing, L., Cockx, P., O'Connor, J.K., Mckellar, R.C., 2020. A newly discovered enantiornithine foot preserved in mid-Cretaceous Burmese amber. PE 3, 212–219. https://doi.org/10.11646/palaeoentomology.3.2.11

Xu, X., Zhou, Z., Wang, Y., Wang, M., 2020. Study on the Jehol Biota: Recent advances and future prospects. Sci. China Earth Sci. 63, 757–773. https://doi.org/10.1007/s11430-019-9509-3

Yamamura, Daigo. Effects of depositional environment, asssemblage taphonomy and diagenesis on vertebrate skeletal preservation in a crevasse splay sandstone, upper Cretaceous Hell Creek Formation, eastern Montana. Diss. Montana State University-Bozeman, College of Letters & Science, 2013.

Yu, Z., Wang, M., Li, Y., Deng, C. and He, H., 2021. New geochronological constraints for the Lower Cretaceous Jiufotang Formation in Jianchang Basin, NE China, and their implications for the late Jehol Biota. Palaeogeography, Palaeoclimatology, Palaeoecology, 583, p.110657.

Zeffer, A., Norberg, U.M.L., 2003a. Leg morphology and locomotion in birds: requirements for force and speed during ankle flexion. Journal of Experimental Biology 206, 1085–1097. https://doi.org/10.1242/jeb.00208

Zeffer, A., Johansson, L.C. and Marmebro, Å., 2003b. Functional correlation between habitat use and leg morphology in birds (Aves). Biological Journal of the Linnean Society, 79(3), pp.461-484.

Zheng, X., O'Connor, J.K., Huchzermeyer, F., Wang, X., Wang, Y., Zhang, X., Zhou, Z., 2014. New specimens of *Yanornis* indicate a piscivorous diet and modern alimentary canal. PLoS ONE 9, e95036. https://doi.org/10.1371/journal.pone.0095036

Zhong, Y., Huyskens, M.H., Yin, Q.Z., Wang, Y., Ma, Q. and Xu, Y.G., 2021. High-precision geochronological constraints on the duration of 'Dinosaur Pompeii'and the Yixian Formation. National Science Review, 8(6), p.nwab063.

Zhou, Z., 2002. A new and primitive enantiornithine bird from the Early Cretaceous of China. Journal of Vertebrate Paleontology 22, 49–57. https://doi.org/10.1671/0272-4634(2002)022[0049:ANAPEB]2.0.CO;2

Zhou, Z., Clarke, J., Zhang, F., 2008. Insight into diversity, body size and morphological evolution from the largest Early Cretaceous enantiornithine bird. Journal of Anatomy 212, 565–577. https://doi.org/10.1111/j.1469-7580.2008.00880.x

Zhou, Z., Zhang, F., 2003. Anatomy of the primitive bird *Sapeornis chaoyangensis* from the Early Cretaceous of Liaoning, China. Can. J. Earth Sci. 40, 731–747. https://doi.org/10.1139/e03-011

Zhou, Z., Zhang, F., 2006. A beaked basal ornithurine bird (Aves, Ornithurae) from the Lower Cretaceous of China. Zoologica Scripta 35, 363–373. https://doi.org/10.1111/j.1463-6409.2006.00234.x

Figure 1 Click here to access/download;Figure;Figure 1.png

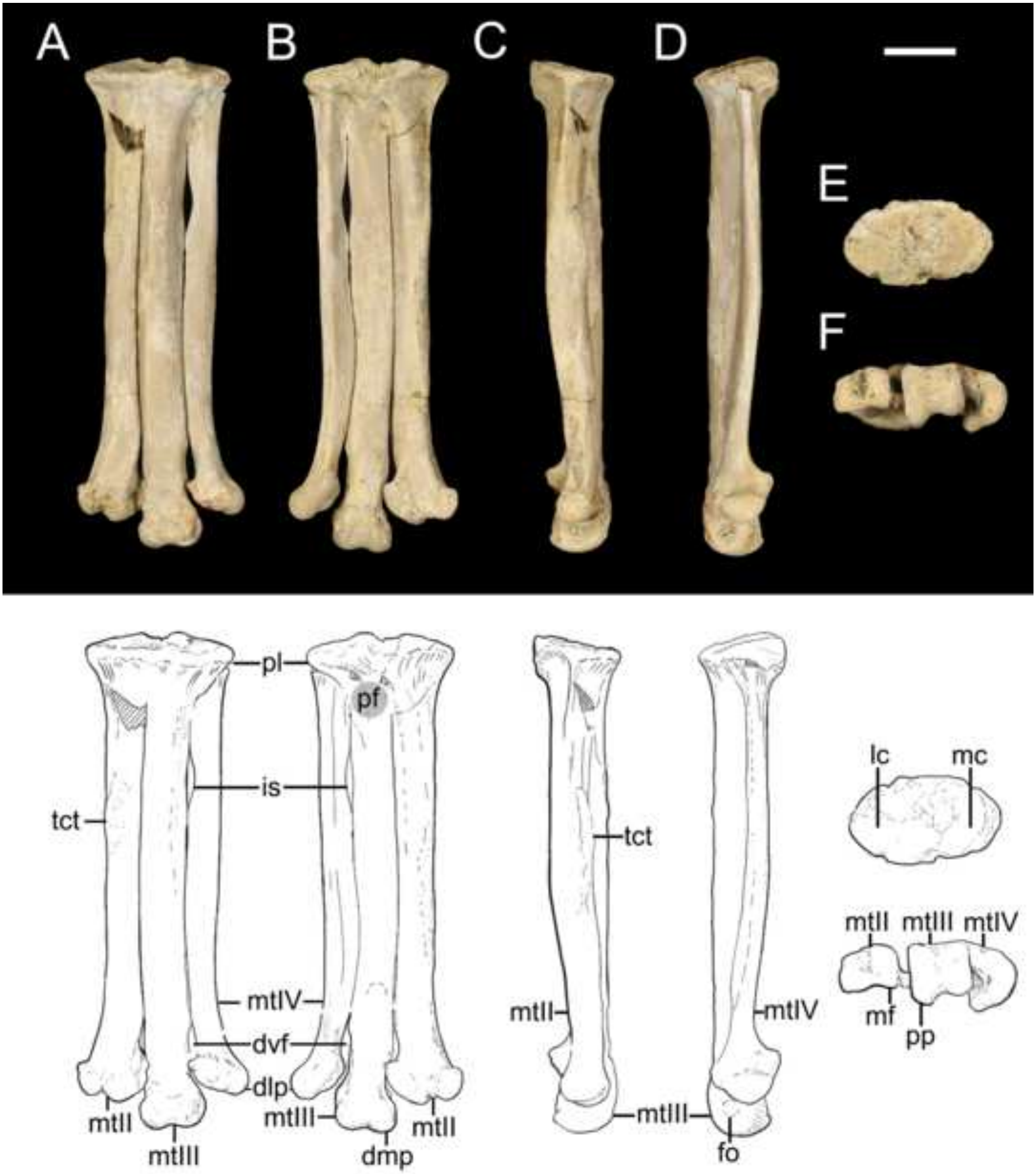

Figure 2

Click here to access/download;Figure;Figure 2.png

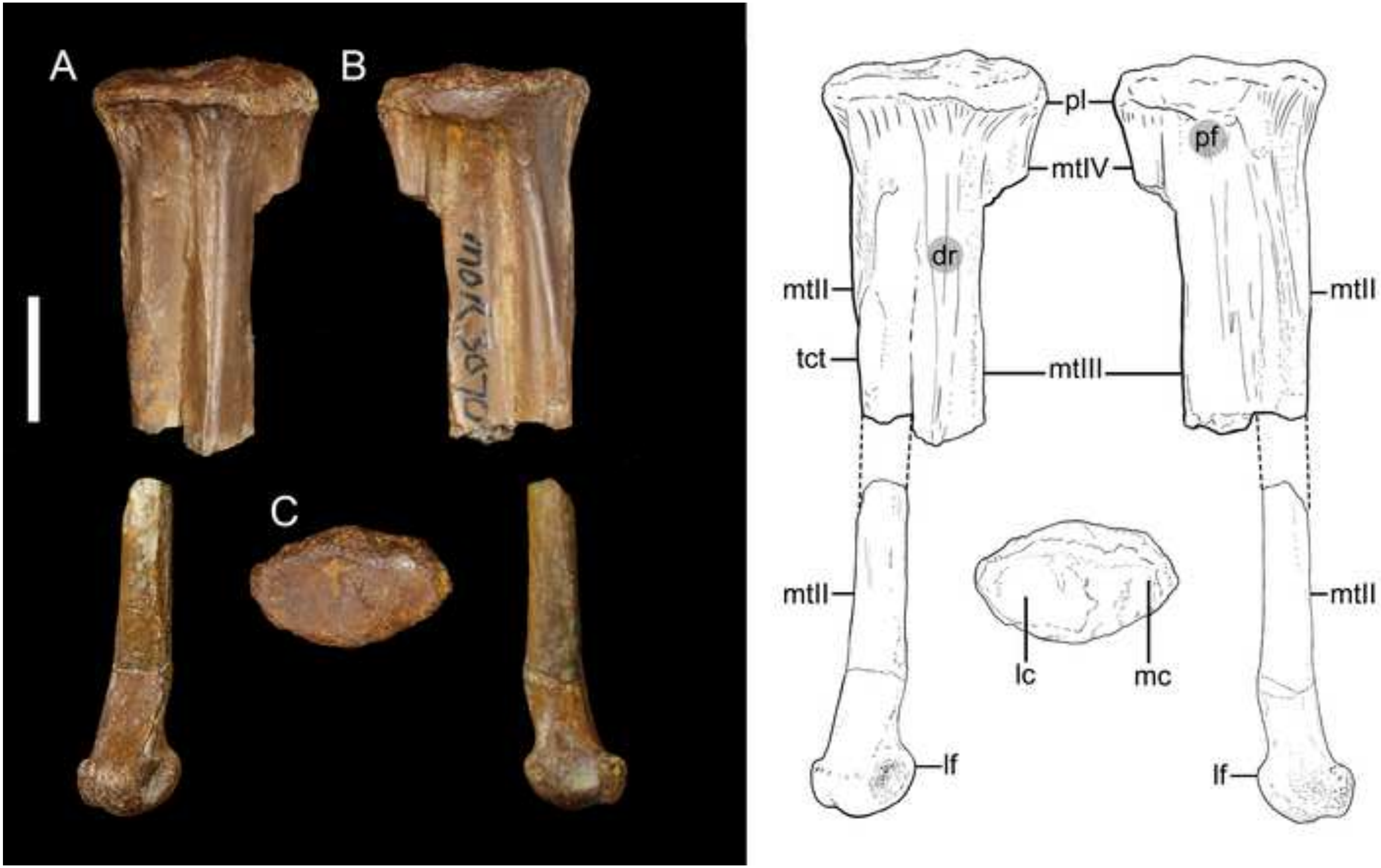

Figure 3

Click here to access/download;Figure;Figure 3.png

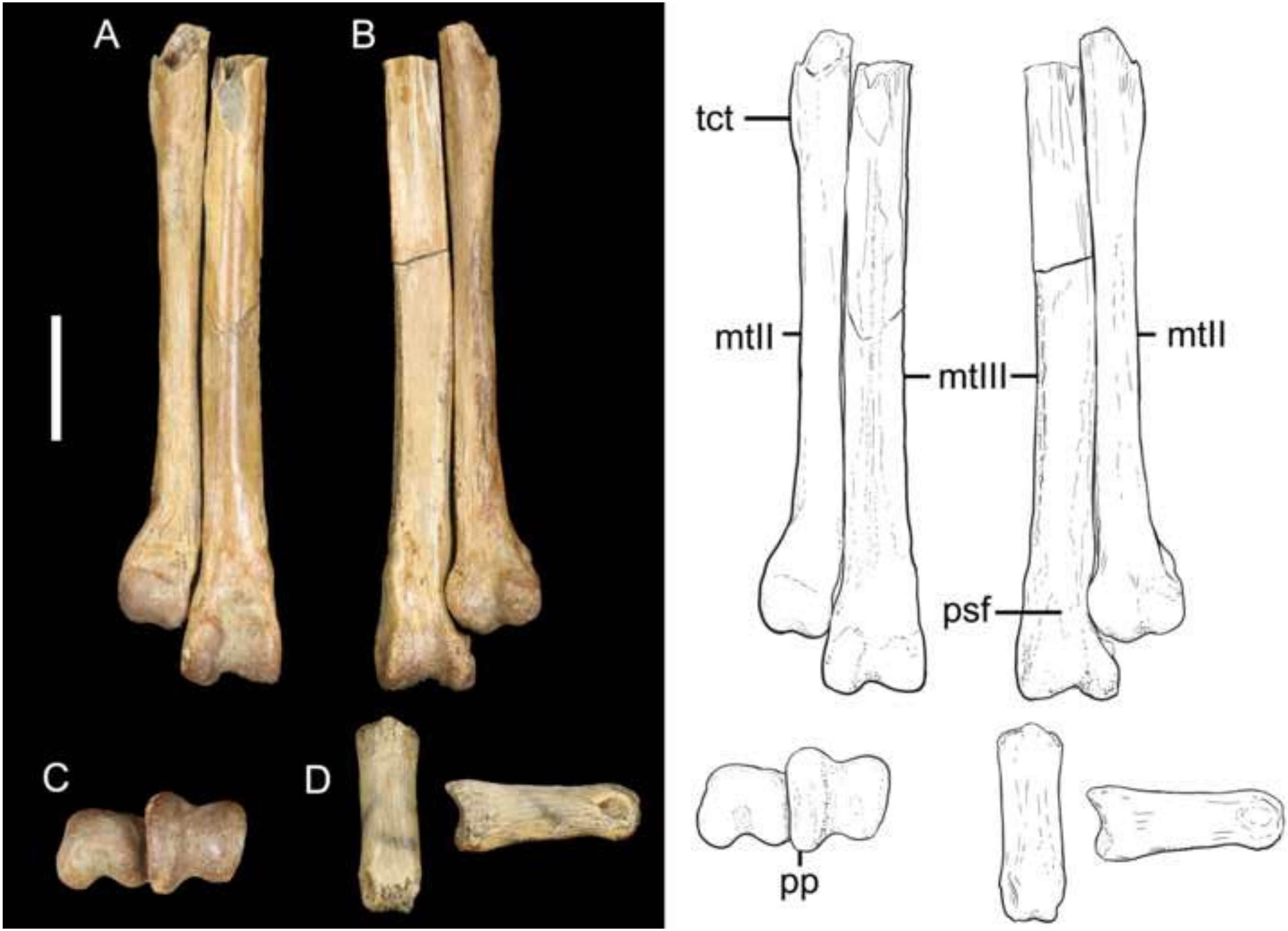

Figure 4 Click here to access/download;Figure;Figure 4.png

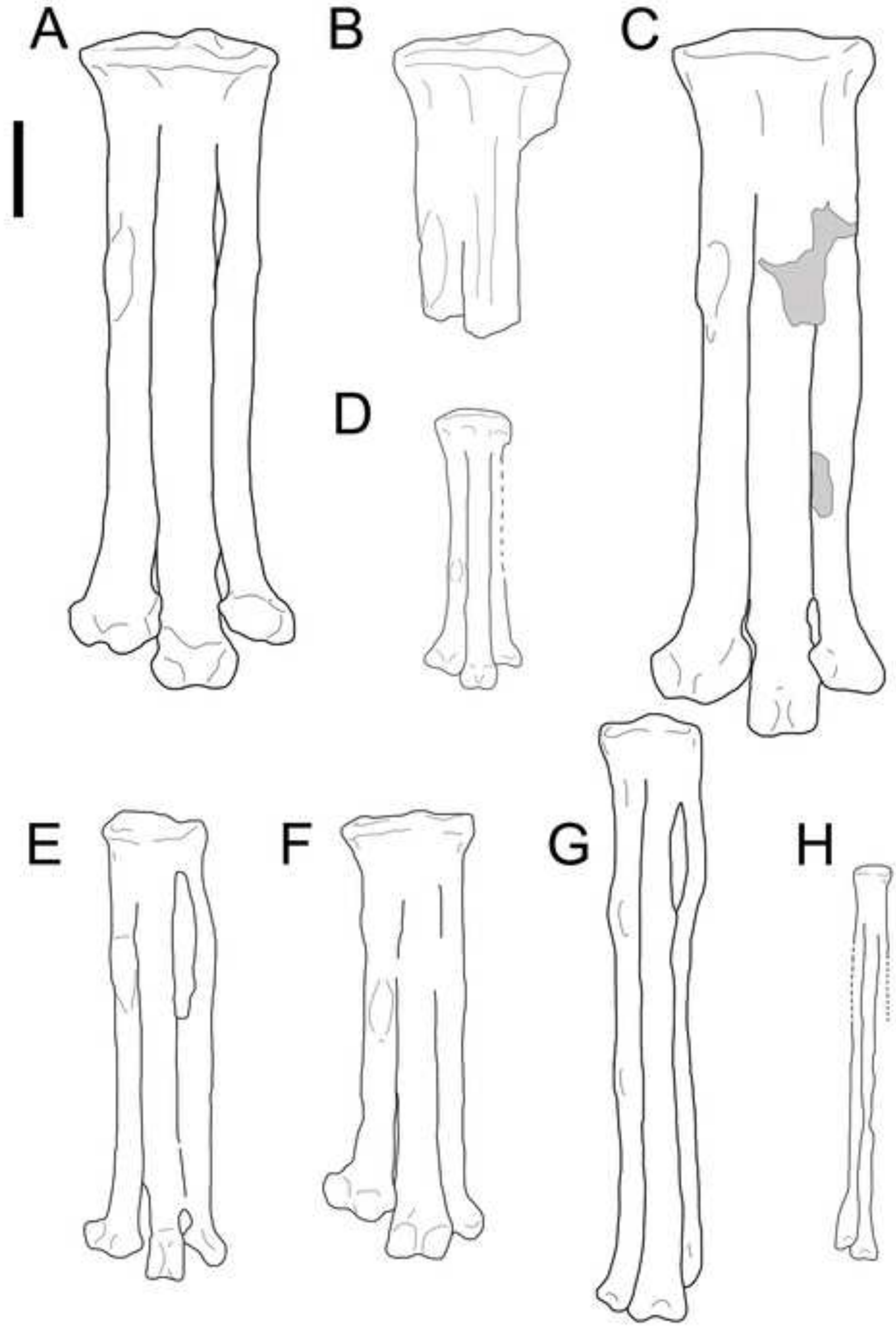

Figure 5 revised

Click here to access/download;Figure;Figure 5.png

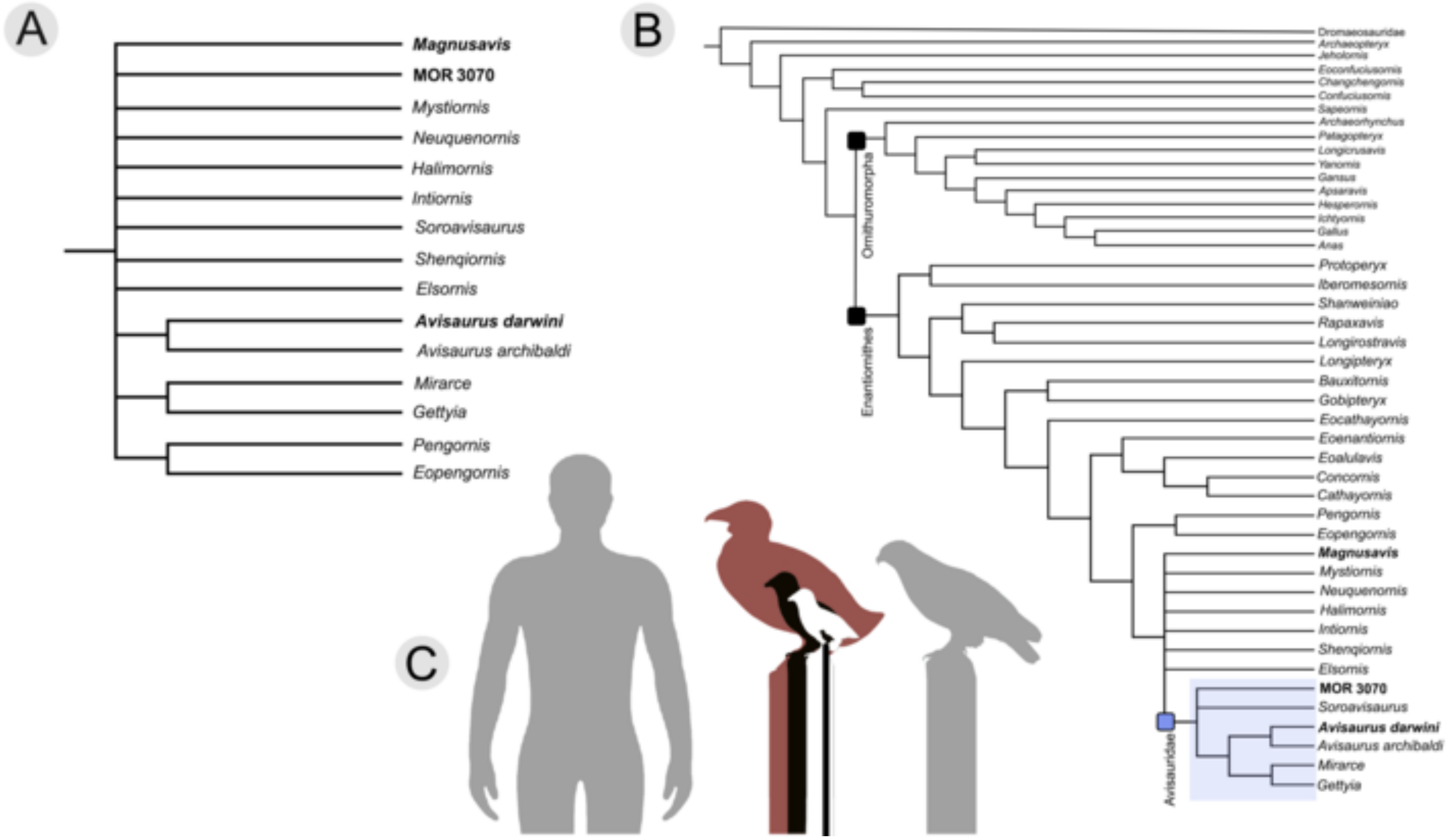

Figure 6 revised

Click here to access/download;Figure;Figure 6 (rev).png

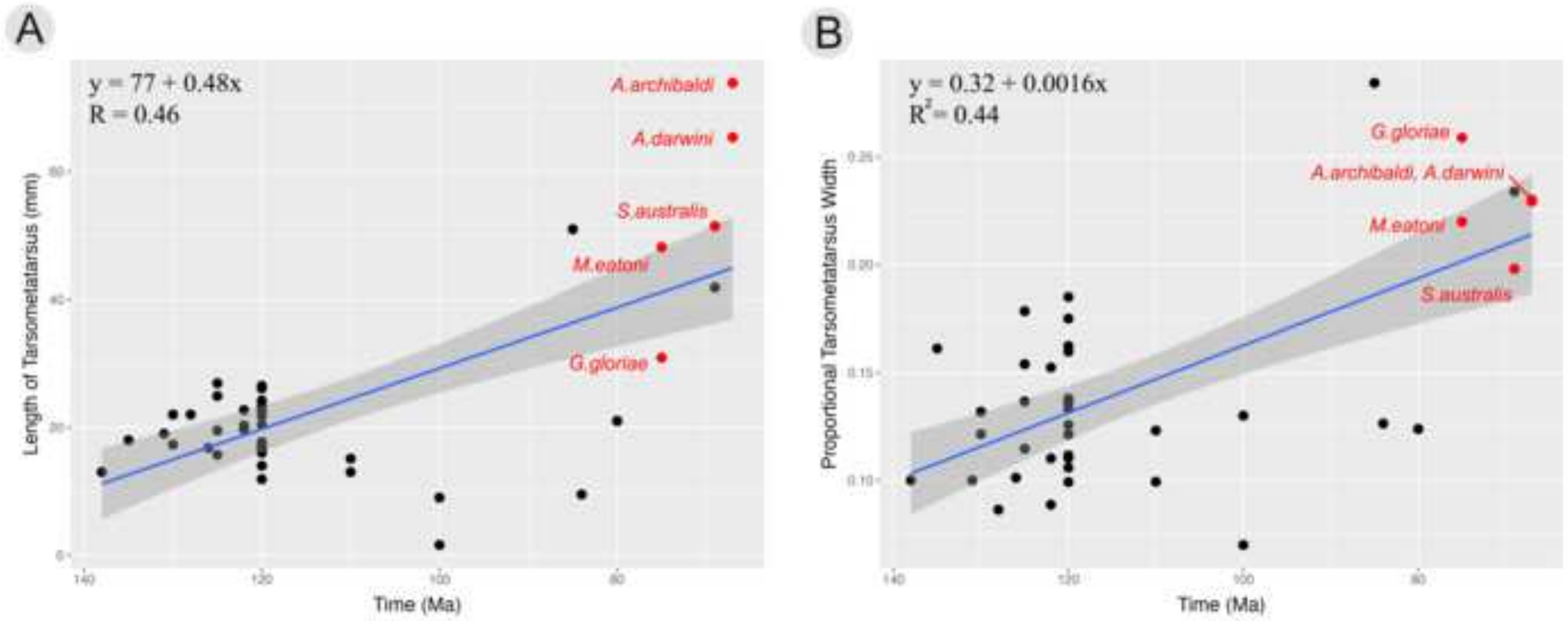

Figure 7

Click here to access/download;Figure;Figure 7.png

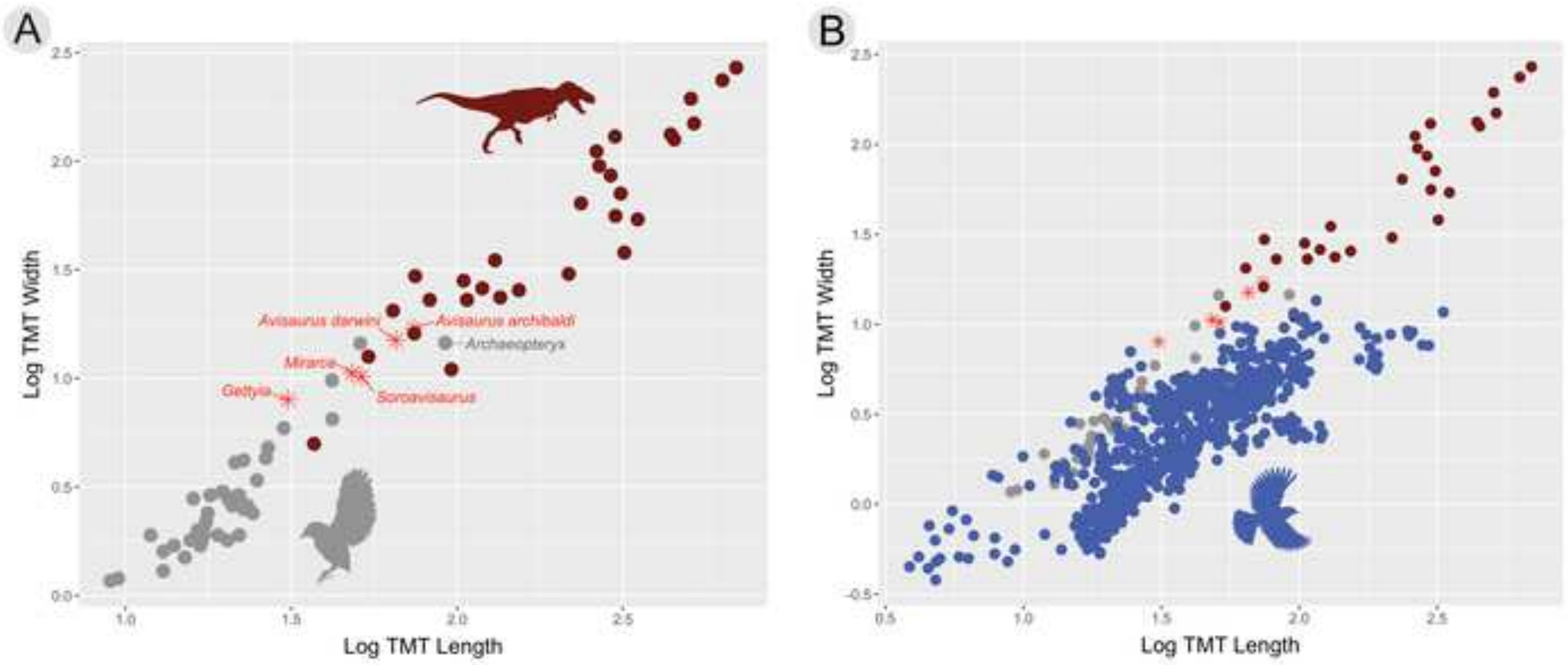

Figure 8 revised

Click here to access/download;Figure;Figure 8 (rev).png

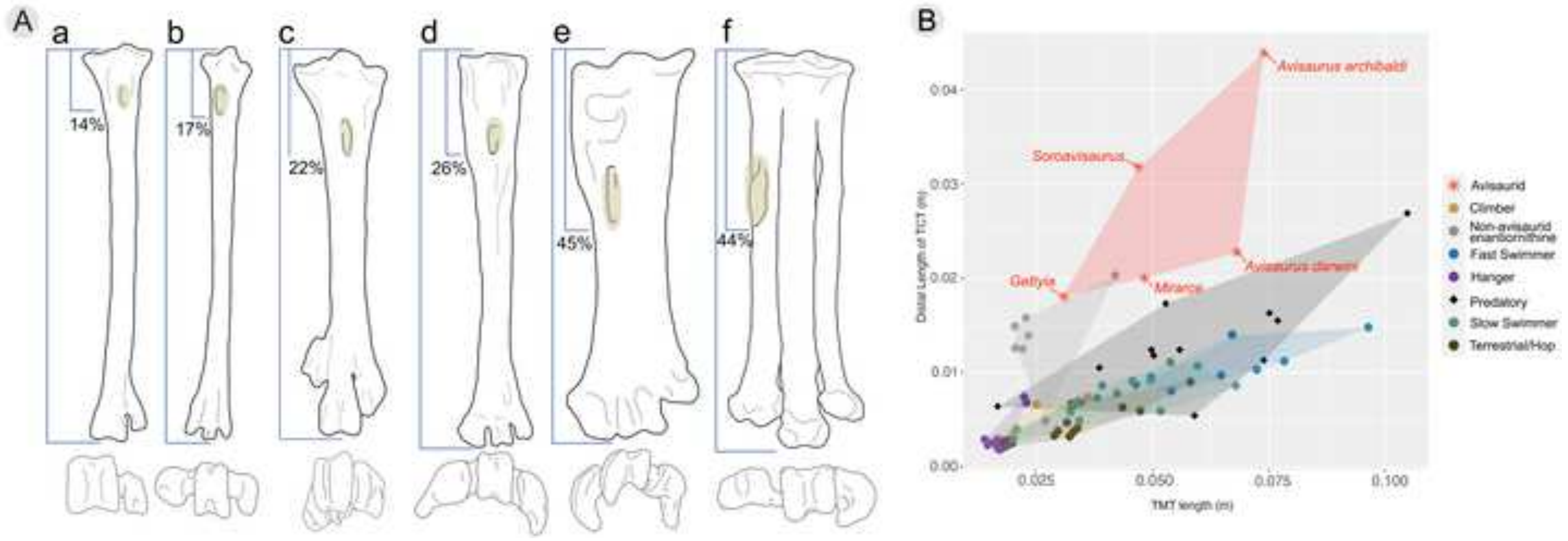

Figure 9

Click here to access/download;Figure;Figure 9.png

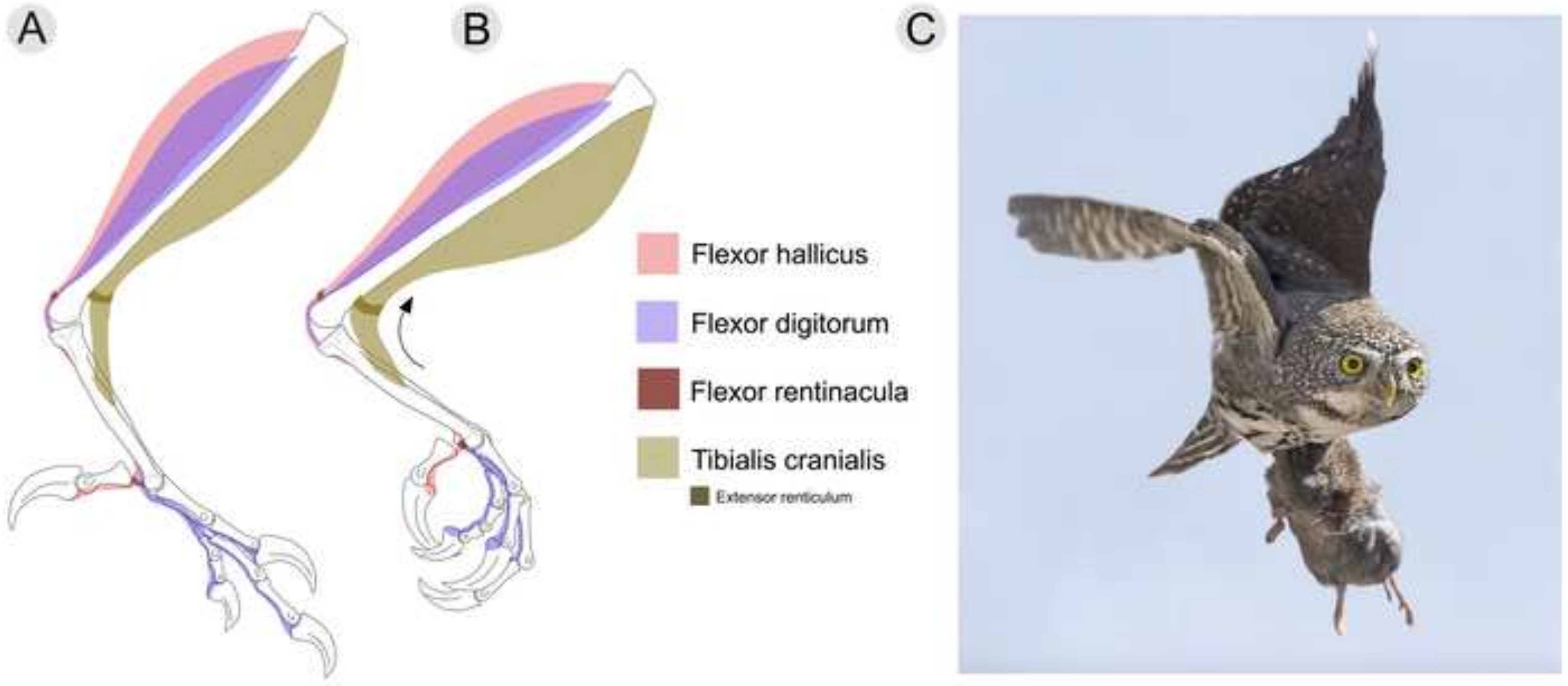

Figure 10

Click here to access/download;Figure;Figure 10.png

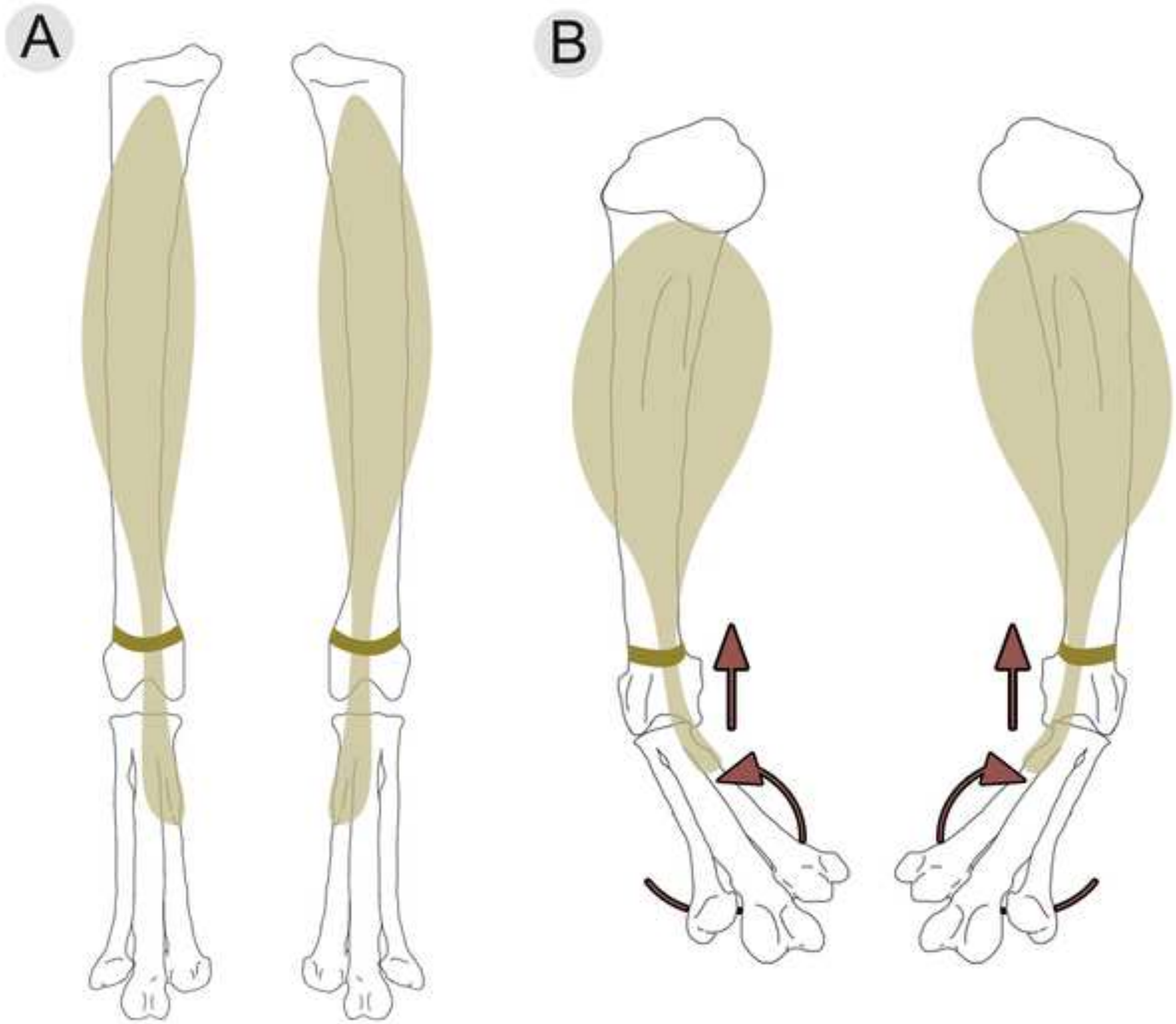

Figure 11 (revised)

Click here to access/download;Figure;Figure 11 (revised).png

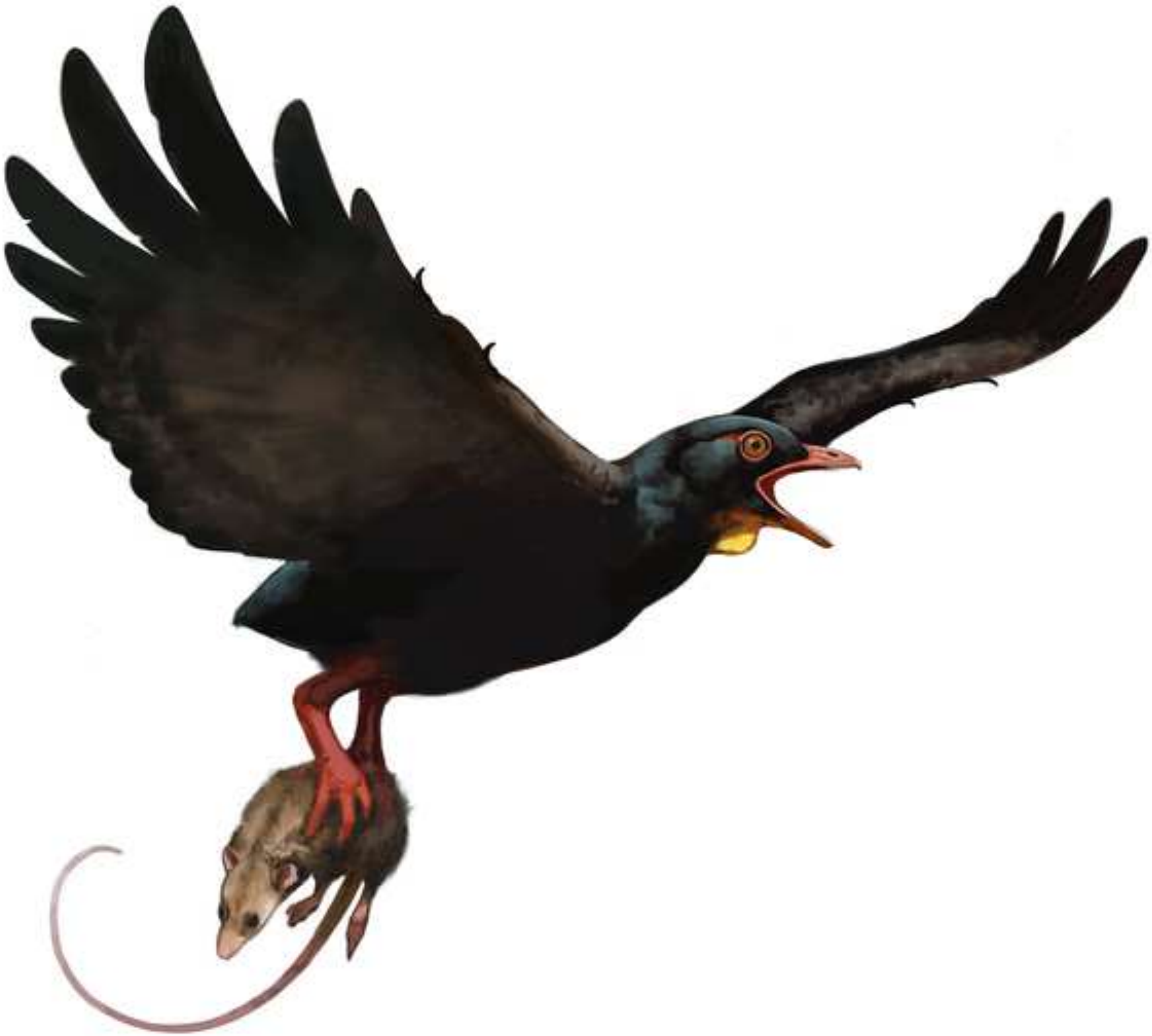

Character Matrix

Click here to access/download
Supporting Information
Phylogenetic Characters.docx

Character Matrix Grading

Click here to access/download
Supporting Information
Grading_file.txt

Figure S1 revised

Click here to access/download
Supporting Information
Figure S1 (rev).png

Fig. S2 revised

Click here to access/download
Supporting Information
FigS2.png

Figure S3

Click here to access/download
Supporting Information
Figure S3.png

Table S1 (csv format)

Click here to access/download
Supporting Information
Table S1.csv

Table S2 (csv format)

Click here to access/download
Supporting Information
Table S2.csv

Table S3 (csv format)

Click here to access/download
Supporting Information
Table S3.csv

Table S4 (csv format)

Click here to access/download
Supporting Information
Table S4.csv

R Code (revised with proper sources)

Click here to access/download

Other

AvisaurusTMT_Rcode (for journal).R

Full title: New enantiornithine diversity in the Hell Creek Formation and the functional morphology of the avisaurid tarsometatarsus

Short title: New Hell Creek enantiornithines and avisaurid tarsometatarsi functional morphology

Alexander D. Clark[1,2*], Jessie Atterholt[3], John B. Scannella[4], Thomas D. Carr[5], Nathan Carroll[6], Jingmai K. O'Connor[2]

[1]Committee on Evolutionary Biology, University of Chicago, Chicago, IL 60637 USA

[2]Negaunee Integrative Resource Center, Field Museum of Natural History, 1400 S Dusable Lake Shore Drive, Chicago, IL. 60605 USA

[3]College of Osteopathic Medicine of the Pacific, Western University of Health Sciences, Pomona, CA, USA

[4]Museum of the Rockies and Department of Earth Sciences, Montana State University, 600 W. Kagy Blvd, Bozeman, Montana, 59717, USA

[5]Department of Biology, Carthage College, Kenosha, WI 53140, USA

[6]Carter County Museum, 306 N Main St. Ekalaka, MT, 59324 USA

Corresponding author: Alexander D. Clark, adclark@uchicago.edu

Abstract

Enantiornithines were the most diverse group of birds during the Cretaceous, comprising over half of all known species from this period. The fossil record and subsequently our knowledge of this clade is heavily skewed by the wealth of material from Lower Cretaceous deposits in China. In contrast, specimens from Upper Cretaceous deposits are rare and typically fragmentary, yet critical for understanding the extinction of this clade across the K-Pg boundary. The most complete North American Late Cretaceous enantiornithine is *Mirarce eatoni*, a member of the diverse clade Avisauridae. Except for *Mirarce*, avisaurids are known only from isolated hindlimb elements from North and South America. Here we describe three new enantiornithines from the Maastrichtian Hell Creek Formation, two of which represent new avisaurid taxa. These materials represent a substantial increase in the known diversity of Enantiornithes in the latest Cretaceous. Re-examination of material referred to Avisauridae through phylogenetic analysis provides strong support for a more exclusive Avisauridae consisting of six taxa. Exploration of the functional morphology of the avisaurid tarsometatarsus indicates potential strong ~~grip~~ constriction and raptorial attributes. The lower aspect ratio of the tarsometatarsus facilitates a more biomechanically efficient lever system ~~for~~ which in extant birds of prey equates to lifting proportionally heavier ~~objects~~prey items. In addition, the proportional size and distal position of the *m. tibialis cranialis* tubercle of the tarsometatarsus is similar to the morphology seen in extant birds of prey. Together with the deeply-grooved metatarsal trochlea facilitating robust and likely powerful pedal digits, morphologies of the hindlimb suggest avisaurids as Late Cretaceous birds of prey.

Keywords: Enantiornithes, paleobiology, Hell Creek Formation, paleoecology, Mesozoic birds

Introduction

Our understanding of Mesozoic avian diversity and evolution is limited by available material, increasing with each new described taxon. Several Lower Cretaceous Lagerstätte~~n~~ have yielded abundant fossils which provide insights to the diversity, ecology, and taxonomy of Mesozoic birds relatively early in their evolution (Zhou and Zhang, 2006). However, information from Upper Cretaceous deposits is far more limited, with the only possible ~~Lagerstätten~~ productive bonebed being a newly emerging Brazilian site dubbed “William’s Quarry” (Chiappe et al., 2022; Wu et al., 2021). Late Cretaceous fossil birds are critical for understanding one of the most important aspects of avian evolution: the selectivity of the end Cretaceous Mass Extinction in which only neornithines survived.

The most speciose clade of Mesozoic birds, constituting over half of all described species, are the enantiornithines (Chiappe and Walker, 2002; O’Connor et al., 2011; Walker, 1981). Members of this clade exhibit morphological diversity and are found on every continent except for Antarctica and continental Africa (Chiappe and Walker, 2002; O’Connor and Chiappe, 2011; Walker, 1981, Zhou et al., 2008). The vast majority of known specimens and described taxa come from Hauterivian-Aptian Lower Cretaceous deposits in Spain and China. As a result, our knowledge of this clade is heavily biased to a narrow time interval of approximately 10 million years (Buscalioni and Poyato-ariza, 2016; Fregenal-Martínez et al., 2017; Yu et al., 2021; Zhong et al., 2021; Zhou and Zhang, 2006).

Enantiornithine fossils from the Late Cretaceous are far more limited but critical for understanding the extinction of this once successful lineage. The only widely recognized clade from Upper Cretaceous deposits is the Avisauridae, known exclusively from North and South American deposits 86-66 Ma. Six genera have been named in the literature: *Avisaurus* (Brett-Surman & Paul, 1985), *Soroavisaurus* (Chiappe, 1993), *Neuquenornis* (Chiappe & Calvo, 1994), *Intiornis* (Novas et al., 2010), *Mirarce* (Atterholt et al., 2018), and *Gettyia* (formerly *A. gloria)* (Atterholt et al., 2018; Varrichio and Chiappe, 1995). Members of this group are mostly described from fragmentary remains, with the tarsometatarsus being the only commonly recovered element and thus critical for recognizing members of this clade. Here, we reevaluate the taxa assigned to the Avisauridae using cladistic analysis and in doing so, remove *Intiornis* and *Neuquenornis* from this group.

Previously, three synapomorphies were hypothesized to unite Avisauridae: a strongly convex dorsal margin of metatarsal III; the plantar projection of the medial condyle of metatarsal III; and a J-shaped laterally compressed metatarsal I (Chiappe and Calvo, 1994). One cladistic analysis suggested this clade also included *Enantiophoenix, Halimornis* and *Concornis* (Cau and Arduini, 2008). However, support for this hypothesis is weak. The tarsometatarsus is unknown in *Halimornis,* and thus referral to the Avisauridae is ambiguous. Similarly, the tarsometatarsus of *Enantiophoenix* is poorly preserved, making the assignment to the Avisauridae based on the convexity of the dorsal surface equivocal. Convexity of the dorsal margin of metatarsal III appears to be more widespread than previously thought, being present in non-avisaurid enantiornithines (e.g., *Imparavis, Feitianius*) (O'Connor et al., 2016; Wang et al., 2024). Additionally, as in *Enantiophoenix,* the lack of convexity in other enantiornithines may be taphonomic.

Assignment to the Avisauridae for *Concornis* was also based on the convexity of the dorsal margin of metatarsal III, and stoutness of the first phalanx of pedal digit I (Cau and Arduini, 2008). In this study, *Halimornis* and *Concornis* were regarded as distinct lineages within the Avisauridae, although with little morphological support (Cau and Arduini, 2008).

The taxon from which this family derives its name, *Avisaurus archibaldi*, is a large enantiornithine, with a tarsometatarsal length (~ 75 mm) comparable to large extant larids (e.g., *Larus marinus, L. glaucescens*~~, 900 – 1800 g~~) and accipitrids (e.g., *Buteo jamaicensis, Accipiter gentilis*~~, ~1400 g~~) (Brett-Surman and Paul, 1985; Winkler et al., 2020a,b). This is in stark contrast to nearly all Early Cretaceous enantiornithines, whose average tarsometatarsal length varies between extant emberizids ~~(old-world sparrows, ~20 g)~~ and turdids ~~(thrushes, ~80 g)~~, tending not to exceed 25 mm in length (Chiappe and Walker, 2002) (Table S1). Avisaurids not only add to the morphological diversity of enantiornithines, but also contribute considerably to our understanding of the apparent trend of increasing body size diversity in the Late Cretaceous (Atterholt et al., 2018; Brett-Surman and Paul, 1985; Chiappe 1996; Chiappe and Walker, 2002).

Here we describe three new enantiornithine specimens, all of which come from the Hell Creek Formation (Latest Cretaceous 68-66 Ma) (Fowler, 2020). One of these new taxa is considered a new species within the genus *Avisaurus* and another is assigned to ~~the~~ *Avisaurus* ~~genus~~sp.. Previously, *A. archibaldi* was the only avisaurid and enantiornithine described from these deposits (Brett-Surman and Paul, 1985). Like previously described North American avisaurids except for *Mirarce*, the new taxa are all represented by isolated tarsometatarsi. Although the material is limited, they possess morphological differences from previously known Late Cretaceous enantiornithines. Here, we revise morphological and phylogenetic diagnoses for the Avisauridae and discuss the significance of these new specimens with regards to their phylogenetic position, implications for size diversity among enantiornithines, and the functional morphology of the avisaurid tarsometatarsus.

INSTITUTIONAL ABBREVIATIONS – DDM, Dinosaur Discovery Museum Kenosha, Wisconsin, United States; MOR, Museum of the Rockies Bozeman, Montana, United States; CCM, Carter County Museum Ekalaka, Montana, United States.

Methods

All necessary permits were obtained for the described study, which complied with all relevant regulations. Specimens were photographed under normal lighting conditions with a Canon 70D dslr camera with a Canon EF 100mm f/2.8 macro lens. Photos of each specimen were assessed in Adobe Photoshop version 24.3.0 and if needed, had aspects of contrast, and color balance edited. Anatomical nomenclature primarily follows Baumel and Witmer (1993) using the English equivalents of the Latin terminology. All measurements used for description and comparisons were recorded in-person using digital calipers (OriginCal1P54). Additional measurements were recorded with ImageJ (Schneider et al., 2012). Aspect ratios of tarsometatarsi were calculated by dividing the mediolateral width at the

proximodistal midpoint by the total proximodistal length at the mediolateral midpoint of the proximal-most margin to the distal-most margin of the proximodistally longest metatarsal.

Size estimates for three avisaurids (*Mirarce*, DDM 1577.730, *A. archibaldi*) were calculated to better compare body sizes to other enantiornithines and extant birds. Even though the tarsometatarsus scales with size in extant birds (Fig. S1), Field et al. (2013) found the greatest predictive power (highest R^2 scores) for determining the mass of birds was the maximum diameter of the glenoid facet of the coracoid (maximum diameter of the coracoid's humeral articulation facet – HAF) and the length of the humerus, both which can be measured in the avisaurid *Mirarce*. To estimate the mass of the two larger avisaurids known only from tarsometatarsi (DDM 1577.730 and *A. archibaldi*) the diameter of the coracoid glenoid and the length of the humerus were approximated by scaling them from *Mirarce* by using a bone which all three taxa preserve: the tarsometatarsus. The tarsometatarsus of DDM 1577.730 and *A. archibaldi* measure 140% and 155% that of *Mirarce* respectively. Using these percentages, we scaled the diameter of the coracoid glenoid and the length of the humerus in *Mirarce* to estimated lengths for DDM 1577.730 and *A. archibaldi* to allow for mass predictions. For all three avisaurids, we averaged the masses of the nearest ten extant species according to data collected by Field et al., (2013) using both the diameter of the coracoid glenoid and the length of the humerus.

Nomenclatural Acts

The electronic edition of this article conforms to the requirements of the amended International Code of Zoological Nomenclature, and hence the new names contained herein are available under that Code from the electronic edition of this article. This published work and the nomenclatural acts it contains have been registered in ZooBank, the online registration system for the ICZN. The ZooBank LSIDs (Life Science Identifiers) can be resolved and the associated information viewed through any standard web browser by appending the LSID to the prefix ""http://zoobank.org/"".

Formatted: Font: Italic

Formatted: Font: Italic

Formatted: Font: Italic

Formatted: Font: Italic

Formatted: Indent: First line: 0"

Formatted: Font: 11 pt

Formatted: Indent: First line: 0.5", Line spacing: Double

Formatted: Font: (Default) Times New Roman, 11 pt

Formatted: Font: (Default) Times New Roman, 11 pt

Formatted: Font: (Default) Times New Roman, 11 pt

The LSID for this publication is: urn:lsid:zoobank.org:pub:53881EA9-CD01-4EAD-9339-ED912FE5C952

The electronic edition of this work was published in a journal with an ISSN, and has been archived and is available from the following digital repositories: PubMed Central, LOCKSS

Systematic Paleontology

Aves - Linnaeus, 1758

Pygostylia Chiappe, 2002

Ornithothoraces Chiappe, 1995

Enantiornithes Walker, 1981

Avisauridae Brett-Surman and Paul, 1985

Diagnosis: Enantiornithines characterized by the combination of a robust tarsometatarsus (mediolateral width at the midpoint at least 20% that of the total proximodistal length); a mediolaterally broad trochlea of metatarsal II relative to III; the presence of a hypertrophied tubercle for the attachment of the *m. tibialis cranialis* on metatarsal II; the *m. tibialis cranialis* tubercle located at least 20% down the length of the tarsometatarsus from the proximal margin; and a well-developed plantar projection of the medial margin of the trochlea of metatarsal III (modified from Atterholt et al., 2018).

Included genera: *Avisaurus*– UCMP 117600, Hell Creek Formation, Late Cretaceous, Maatstrichtian, Garfield County, Montana (Brett-Surman and Paul, 1985); *Soroavisaurus*– PVL 4690, Lecho Formation, Late Cretaceous, Maastrichtian, Salta Province, Argentina (Chiappe, 1993); *Mirarce*– UCMP 139500, Kaiparowits Formation, Late Cretaceous, Campanian, Grand Staircase-Escalante National Monument,

Formatted: Font: (Default) Times New Roman

Formatted: Line spacing: Double

Formatted: Font: (Default) Times New Roman

Formatted: Font: 11 pt

Formatted: Font: (Default) Times New Roman, 12 pt, Font color: Auto

Formatted: Space Before: 0 pt, After: 0 pt

Formatted: Font: 11 pt

Formatted: Normal, Indent: First line: 0", Space Before: Auto, After: Auto

Formatted: Font: (Default) TimesNewRomanPSMT, Font color: Black

Utah (Atterholt et al., 2018); *Gettyia*– MOR 553E/1.19.91.64, Two Medicine Formation Late Cretaceous, Campanian, Glacier County, Montana (Atterholt et al., 2018).

Avisaurus Brett-Surman and Paul, 1985

Diagnosis: Avisaurid enantiornithines characterized by the following combination of characters: distal extent of metatarsal II approximately equal to that of metatarsal IV; the proximoplantar surface excavated by a pair of small fossae located where the medial and lateral margins of metatarsal III meet the plantar labrum, hereafter referred to as the proximoplantar fossae (*nomen novum*) (modified from Chiappe, 1993) (Fig. S2).

Formatted: Font: Italic

Included species: *Avisaurus archibaldi* – UCMP 117600, Late Cretaceous, Maatstrichtian, Garfield County, Montana (Brett-Surman and Paul, 1985); *Avisaurus darwini* – DDM 1577.730, Hell Creek Formation, Late Cretaceous, Maastrichtian, Carter County, Montana (this publication).

Avisaurus darwini sp. nov. (Fig. 1, Table 1)

Holotype: DDM 1577.730 is a complete right tarsometatarsus collected in July of 2022

Etymology: The specific name “*darwini*”, is in honor of Charles Darwin, whose momentous research and publications helped define the field of evolutionary biology. *Avisaurus darwini*, Darwin’s bird lizard.

Type horizon and locality: Hell Creek Formation, Latest Cretaceous, Maastrichtian (approximately 66 Ma). DDM 1577.730 was collected in Carter County, Montana at a locality under the auspices of the Bureau of Land management. The specimen was collected from the Madison Shark Spine locality (DDM-18-234,1N 55E 11) within a grey sand multitaxic microvertebrate site.

FIGURE 1. The left tarsometatarsus of *Avisaurus darwini* (DDM 1577.730) in A) dorsal, B) plantar, C) right lateral, D) left lateral, E) proximal, and F) distal aspects. Abbreviations: dlp, distal lateral projection; dmp, distal medial projection; dvf, distal vascular foramen; fo, fossa (~~possible~~ collateral ligament fossa); is, intermetatarsalian space; lc, lateral cotyle; lf, laterally-projecting flange; mc, medial cotyle; mtII, metatarsal II; mtIII, metatarsal III; mtIV, metatarsal IV; pf, proximoplantar fossae; pl, proximal labrum; pp, plantar projection; tct, *m. tibialis cranialis* tubercle. Scale bar 10mm. Line illustrations done by Samantha Clark.

	Avisaurus darwini	***MOR 3070***	***Magnusavis ekalakaensis***
**Proximal Surface**			
Width	20.24	18.56	-
Height	11.65	11.15	-
**Cranial Aspect**			
MT II Length	62.93	*58.68	-
MT II Width (midpoint)	4.91	-	3.12
MTIII Length	65.38	-	-
MT III Width (midpoint)	6	-	4.8
MT IV Length	60.4	-	-
MT IV Width (midpoint)	4.98	-	-
**Distal Aspect**			
MT II Trochlea Width	8.07	-	7.38
MT II Trochlea Height	5.22	-	5.78
MT III Trochlea Width	8.18	-	8.27
MT III Trochlea Height	6.62	-	5.87
MT IV Trochlea Width	9.48	-	-
MT IV Trochlea Height	4.64	-	-
**Aspect ratio of Tmt**	1:4	*1:4	-

TABLE 1. Measurements (in mm) of each newly described specimen. For MOR 3070, asterisks represent approximations. Total length of metatarsal II was estimated by scaling MOR 3070 to *A. darwini*, ~~who~~ which is nearly the same size.

Diagnosis –A large accipitrid-sized bird identified as an avisaurid based on the strong plantar projection of the medial condyle of the trochlea of metatarsal III; a robust tarsometatarsus (mediolateral width at the midpoint greater than 20% that of the total proximodistal length); a mediolaterally broad trochlea of metatarsal II relative to III; the presence of a hypertrophied *m. tibialis cranialis* tubercle on metatarsal II located ~ 30% down the tarsometatarsus. DDM 1577.730 is referable to the genus *Avisaurus* based on the following features: the distal extent of metatarsal II approximately equal to that of metatarsal IV; the presence of proximoplantar fossae; a metatarsal II exhibiting a laterally-projecting flange of bone from the plantar margin. DDM 1577.730 can be differentiated from other avisaurids based on the following unique combination of features: a proximally located intermetatarsal space between metatarsals III and IV which is proximodistally proportionally shorter than that of *Soroavisaurus*; metatarsal II's laterally-projecting flange of bone closes the intertrochlear incisure, contacting the distomedial margin of the metatarsal III trochlea (this feature is more pronounced than that in the lateroplantarly-projecting flange in *A. archibaldi*); both medial and lateral margins of the trochlea of metatarsal III terminate distally at the same level, disparate from *Soroavisaurus* (lateral margin extending distally) and *A. archibaldi* (medial margin extending distally).

Avisaurus sp. (Fig. 2, Table 1)

Material: MOR 3070 is a partial right tarsometatarsus preserving most of metatarsal II (distal portion disarticulated), < 50% of metatarsal III, and the proximal portion of metatarsal IV. Some additional disarticulated fragments are probably referable to the specimen but lack diagnostic morphologies.

Locality: Hell Creek Formation, Latest Cretaceous, Maastrichtian (approximately 68 - 66 Ma). MOR locality no. HC-720 "Daigo's Site". MOR 3070 was discovered by Daigo Yamamura in Makoshika State

Park, Glendive, Montana and collected by a field crew led by Frankie Jackson of Montana State University (Yamamura, 2013).

FIGURE 2. The left partial tarsometatarsus of *Avisaurus* sp. MOR3070 in A) dorsal, B) plantar, and C) proximal aspects. The distal portion of metatarsal II is placed in the approximate place if it was articulated. Abbreviations: dr, dorsal ridge: lc, lateral cotyle; lf, laterally-projecting flange; mc, medial cotyle; mtII, metatarsal II; mtIII, metatarsal III; mtIV, metatarsal IV; pf, proximoplantar fossae; pl, proximal labrum; tct, *m. tibialis cranialis* tubercle. Scale bar 10mm. Line illustrations done by Samantha Clark.

Comment – A large accipitrid-sized enantiornithine with plantar-projecting medial and lateral plantar crests; dorsal face of metatarsal II is mediolaterally wider than metatarsal III proximally; the lateral cotyle elevated slightly proximally relative to the medial cotyle. MOR 3070 is referable to the genus *Avisaurus* based on the two proximoplantar fossae in plantar aspect, and a metatarsal II trochlea exhibiting a lateroplantarly-projecting flange of bone from the plantar margin. MOR 3070 differs from other closely related enantiornithines (avisaurids) based on the following unique combination of features: lateral to medial tapering of the proximal labrum's dorsal margin; a dorsally-projecting *m. tibialis cranialis* tubercle rather than dorsomedially projection as in *A. archibaldi* and *A. darwini*; metatarsal II mediolaterally greater than that of III in dorsal aspect along its proximal preserved portion; a well-developed dorsally-projecting ridge on the dorsal face of metatarsal III beginning prominently below the labrum shelf and continuing distally down the dorsal surface of the remaining portions of metatarsal III (forming a triangular cross section in plantar aspect); plantar margin of the trochlea of metatarsal II with a lateroplantarly-projecting flange, similar to *A. archibaldi* but disparate from the lateral-projecting morphology in *A. darwini*.

Aves Linnaeus, 1758

Pygostylia Chiappe, 2002

Ornithothoraces Chiappe, 1995

Enantiornithes Walker, 1981

Magnusavis ekalakaenis gen. et sp. nov. (Fig. 3, Table 1)

Holotype: CCM V2019.5.1 is a partial right tarsometatarsus, missing the proximal portions of metatarsals II and II, and all of metatarsal IV. A single phalanx is preserved, though digit assignment is uncertain.

Etymology: In Latin, "*Magnus*" meaning big, and "*avis*" meaning bird, and "*ekalakaensis*" in honor of the town of Ekalaka, Montana, close to where this specimen was discovered. Ekalaka is Lakota for "one who wanders". *Magnusavis ekalakaenis*, Ekalaka's big bird.

Locality: Hell Creek Formation, Late Cretaceous, Maastrichtian (approximately 66 Ma). The specimen was collected in Carter County, Montana at a locality under the auspices of the Bureau of Land management (Permit MTM 108846).

FIGURE 3. The partial left tarsometatarsus of *Magnusavis ekalakaensis* (CCM V2019.5.1) in A) dorsal, B) plantar, and C) distal aspects, D) and the single preserved pedal phalanx in dorsal and right lateral aspects. Abbreviations; mtII, metatarsal II; mtIII, metatarsal III; pp, proximal projection; psf, plantar supratrochanter fossa; tct, *m. tibialis cranialis* tubercle. Scale bar 10mm. Line illustrations done by Samantha Clark.

Diagnosis – A larid-sized enantiornithine differentiated from other taxa, including the closely-related avisaurids, based on the following unique combination of features: distal expansion of metatarsals II and

III (all avisaurids retain nearly equal medial and lateral margins of the metatarsals down their lengths); a medial and lateral-rimmed (i.e., well-excavated) plantar supratrochanter fossa on metatarsal III; and an articular face on metatarsal III (for metatarsal IV) terminating distal to that in *A. archibaldi*.

Phylogenetic Analysis

These three new specimens were added to a modified version of the character matrix by Atterholt et al. (2018). We added three new characters to assess the relative robustness of the tarsometatarsus [Character 253], presence of the proximoplantar fossae bordering metatarsal III [Character 254], and the presence of the intermetatarsal space between metatarsals III and IV proximal to the midpoint of the tarsometatarsus [Character 255]. The final matrix, consisting of 45 taxa with scores across 255 characters, was analyzed in TNT (Goloboff et al., 2008). For scoring, see Supplementary Information. Neornithes were represented by *Anas* and *Gallus*, and Dromaeosauridae was used as the outgroup. We conducted a heuristic search retaining the single shortest tree out of every 1000 trees followed by a second round of tree-bisection reconnection (TBR). The first round of TBR produced 36 trees with a length of 803 steps; the second round of TBR produced 4207 trees of the same length.

Results

Morphological descriptions

DDM 1577.730 – Holotype of *Avisaurus darwini*

urn:lsid:zoobank.org:act:E5235AC4-571C-4606-90D7-C25BCDC3CF2A

Formatted: Font: (Default) Times New Roman

Formatted: Font: 11 pt

Formatted: Font: (Default) Times New Roman

Formatted: Font: 11 pt

Formatted: Indent: First line: 0"

The metatarsals are all fused proximally to each other and the distal tarsals. The proximal articular surface is oval in shape with the lateral cotyle slightly elevated and larger relative to the medial cotyle as in *A. archibaldi*. It is greater than the cross-section of metatarsals II-IV such that it forms a well-developed labrum that overhangs the metatarsal shafts in both dorsal and plantar aspects as in *Mirarce* and *A. archibaldi* (Atterholt et al., 2018; Brett-Surman & Paul, 1985). Like *A. archibaldi*, this is more pronounced in plantar aspect. In plantar view, just below the labrum, the proximal-most lateral and medial portions of the metatarsal III shaft are each excavated by a small fossa, referred to as the proximoplantar fossae (Fig. 1, B). These fossae are also present, although less pronounced, in *A. archibaldi* and MOR 3070, but absent in other avisaurids.

In dorsal aspect, the metatarsals do not distally expand mediolaterally, similar to *A. archibaldi*, *Gettyia*, and *Mirarce* (Atterholt et al., 2018; Brett-Surman & Paul, 1985) (Fig. 4). As in *Soroavisaurus*, there is a large intermetatarsal space between metatarsals III and IV (Fig. 1, A&B), originally referred to as a “fenestra” Chiappe (1993). This structure may be analogous to the lateral proximal vascular foramen in extant birds which allows for anastomosis between the dorsal and plantar surfaces (Chiappe, 1993; Jadwiszczak, 2015; Schwehn et al., 2024). The mediolateral width of the space measures approximately 45% of the midpoint of metatarsal IV. In *Soroavisaurus*, this space is proximodistally longer and begins more proximally, extending from nearly 5% to 36% the length of the tarsometatarsus (Chiappe, 1993). In *Intiornis* the space extends from 12% to 30% the length of the tarsometatarsus (Novas et al., 2010).

Metatarsal III is the longest, as in all avisaurids, and most enantiornithines (Atterholt et al., 2018; Chiappe and Walker, 2002). The proximodistal length of metatarsals II and IV measures 96% and 92% that of metatarsal III respectively. Metatarsal III also exhibits the greatest mediolateral width measured at the midpoint. Metatarsals II and IV measure 82% and 83% the width of metatarsal III, respectively (Table 1). Like other avisaurids (but also some non-avisaurid enantiornithines), the dorsal surface of metatarsal III is strongly convex forming a weak ridge for most of its length. The shaft of metatarsal II is similarly convex, unlike other avisaurids where this face is comparatively flatter. The distal portion of metatarsal II and its trochlea deflects medially as in *A. archibaldi*, *Gettyia*, and *Mirarce* (Atterholt et al., 2018; Brett-

Surman & Paul, 1985). The shaft of metatarsal IV is cranioplantarly compressed similar to *A. archibaldi* and *Gettyia*. The distal portion of the metatarsal gently curves laterally just proximal to the mediolaterally expanded trochlea more so than *Soroavisaurus* and *Mirarce* (Atterholt et al., 2018; Brett-Surman & Paul, 1985; Chiappe, 1993).

FIGURE 4. Tarsometatarsus size variation among the Avisauridae and close relatives. A) *Avisaurus darwini*, B) MOR3070, C) *Avisaurus archibaldi*, D) *Gettyia*, E) *Soroavisaurus*, F) *Mirarce*, G) *Intiornis*, H) *Neuquenornis*. All to scale. Scale bar 10 mm.

On the craniomedial surface of metatarsal II there is a large and rugose tubercle for the attachment of the *m. tibialis cranialis* (Fig. 1, A&C). This structure is elongate, beginning 29% of the way down metatarsal II and extending for 15% of its total length, similar to *A. archibaldi* both in size and position (Brett-Surman & Paul, 1985). Distally, there is a weakly-developed intertrochlear incisure between metatarsals II and III. The space extends for approximately 11% that of the total length of metatarsal III. The distal vascular foramen between trochlea III and IV begins 78% down the length of metatarsal III and terminates at approximately 86%. The mediolateral width of this space measures approximately 34% that of the midpoint width of metatarsal IV.

The laterally-projecting flange of the lateral trochlea of metatarsal II expands such that the trochlea along the plantar margin is wider than that of metatarsal III, unlike *Soroavisaurus*. In *A. archibaldi*, this flange is proportionally smaller, projects lateroplantarly, and does not fully close the gap between the trochlea of metatarsal II and III(Brett-Surman & Paul, 1985). In *Mirarce*, this feature is also present, but is located more distally on metatarsal II and expands distolaterally (Atterholt et al., 2018). In *A. darwini*, this distal laterally-projecting flange contacts that distomedial margin of metatarsal III, closing the gap between the trochlea of metatarsals II and III. This closure creates a condition analogous to the distal vascular foramen between the trochlea of metatarsal III and IV (Atterholt et al., 2018).

The medial and lateral condyles of the metatarsal III trochlea terminate approximately at the same level, whereas the medial margin extends farther distally in *A. archibaldi* (Brett-Surman & Paul, 1985). As in *A. archibaldi* and *Mirarce*, the medial condyle of the trochlea of metatarsal III exhibits greater plantar projection than the lateral condyle (Atterholt et al., 2018; Brett-Surman & Paul, 1985). The trochlea of both metatarsals II and III are ginglymous, and exhibit well-developed, excavated, intercondylar grooves. These well-developed grooves are also present in *A. archibaldi*, *Mirarce*, *Gettyia*, and *Soroavisaurus* (Atterholt et al., 2018; Brett-Surman & Paul, 1985; Chiappe, 1993). The trochlea of metatarsal IV has a reduced medial condyle such that it appears as a single, plantomedially excavated condyle, similar to other enantiornithines (e.g., *A. archibaldi, Avimaia*, *Imparavis*, *Mirarce)* (Atterholt et al., 2018; Bailleul et al., 2019; Brett-Surman & Paul, 1985; Wang et al., 2024). In distal view, the trochlea of metatarsal IV is C-shaped (medially concave), and projects farther plantarly than even the medial condyle of the metatarsal III trochlea, as in other avisaurids (e.g., *Mirarce, A. archibaldi*) (Atterholt et al., 2018; Brett-Surman & Paul, 1985).

Specimen MOR 3070 - *Avisaurus* sp.

The metatarsals are all fused proximally to each other and the distal tarsals (Fig. 2). Similar to other *Avisaurus*, the proximal articular surface is oval with the lateral cotyle slightly elevated relative to the medial cotyle. The proximal labrum is well-developed as in *A. archibaldi*, *A. darwini*, and *Mirarce* (Atterholt et al., 2018; Brett-Surman & Paul, 1985). However, unlike *A. darwini*, and *Mirarce*, MOR 3070 exhibits greater lateral to medial tapering of the labrum thickness along the dorsal margin (Fig. 2, A; Atterholt et al., 2018). In plantar aspect, the labrum is dorsoventrally thickest at its midpoint (i.e., above the midline of metatarsal III). Characteristic of the genus *Avisaurus*, two proximoplantar fossae bordering metatarsal III are present. These fossae are not as deep as in *A. darwini*.

Though incomplete, MOR 3070 would have had a low aspect ratio of the tarsometatarsus (~ 1:4) like other avisaurids (e.g., *Mirarce*, *Gettyia*, and *Avisaurus*). Proximally, metatarsal II is the

mediolaterally widest metatarsal, with the width of metatarsal III measuring approximately 87% that of the metatarsal II. This in contrast to *Mirarce*, *A. archibaldi*, and *A. darwini*, where even proximally the width of metatarsal II is less than that of III (Atterholt et al., 2018). Similar to members of *Avisaurus*, MOR 3070 exhibits a well-developed *m. tibialis cranialis* tubercle which if scaled to *Avisaurus* would place it approximately 30% down the length of metatarsal II. In contrast to other avisaurids, the *m. tibialis cranialis* tubercle of MOR 3070 projects more dorsally as opposed to dorsomedially. The disarticulated distal portion of metatarsal II exhibits similarities to *A. archibaldi* in that it exhibits a lateroplantarly-projecting flange (Brett-Surman & Paul, 1985). This is disparate from the laterally-projecting morphology exhibited in *A. darwini*. The relationship of this flange with the trochlea metatarsal III is unknown, but similar to *A. archibaldi*, it likely did not contact metatarsal III (this flange contacts the trochlea of metatarsal III in *A. darwini*). Beginning prominently below the labrum shelf, a well-developed dorsally-projecting ridge extends distally down the remaining portion of the dorsal surface of metatarsal III (forming a triangular cross section in plantar aspect). This feature is absent in *A. darwini*, and weakly developed in *A. archibaldi*.

CCM V2019.5.1 – Holotype of *Magnusavis ekalakaenis*

urn:lsid:zoobank.org:act:4A5DF7A9-06FD-45A0-B7CA-726B1936D7DA

The holotype of *M. ekalakaenis* consists of partial metatarsals II and III and a single phalanx (Fig. 3). Unlike avisaurids, both preserved metatarsals exhibit subtle distal expansion. The proximal-most mediolateral width of metatarsal III measures 76% that of the distal-most portion (proximal to the trochlea) (*A. darwini*, 100%, *A. archibaldi*, 96%). Metatarsal III is both proximodistally longer and mediolaterally wider than metatarsal II along its length, typical of most enantiornithines (Chiappe and Walker, 2002).

Formatted: Font: (Default) Times New Roman
Formatted: Font: 11 pt
Formatted: Font: (Default) Times New Roman
Formatted: Font: 11 pt

The shaft of metatarsal II is rounded and smooth, in contrast to the well-developed peaked ridge in MOR 3070 and *A. darwini*. The distomedial deflection of metatarsal II is weakly-developed compared to the condition seen in avisaurids, exhibiting a nearly closed intertrochlear incisure between metatarsal II and III. Though not as pronounced or as large as the condition seen in avisaurids, the *m. tibialis cranialis* tubercle is well developed when compared to most enantiornithines (Atterholt et al., 2018; Brett-Surman & Paul, 1985). Similar to metatarsal II, the dorsal surface of metatarsal III is only weakly convex, in contrast to the strongly convex morphology in avisaurids (e.g., *Mirarce, A. archibaldi,* MOR 3070, and, *A. darwini*).

The condyles of the metatarsal III trochlea terminate at the same level distally. Like avisaurids, the intercondylar grooves are well-developed in both metatarsals II and III, particularly in III. The medial condyle of the metatarsal III trochlea exhibits greater dorsal and plantar projection relative to the lateral condyle. The plantar supratrochanter fossa is also well developed, and clearly demarcated lateral and medially. The mediolateral width of this depression measures approximately 75% the total width of the trochlea of metatarsal III. The lateral fossa of metatarsals II and III are well-developed.

Though digit assignment of the phalanx is uncertain, it resembles the first phalanx of pedal digit II in *Mirarce*, albeit much more dorsoventrally deep (lateral aspect ratio *Magnusavis* 1:3, *Mirarce* 1:6). The aspect ratio of the phalanx is approximately 1:3, in contrast to that seen in Mirarce (~ 1:5).

Mass Estimates

Based on the maximum diameter of the glenoid facet of the coracoid and the length of the humerus in *Mirarce*, we calculated an estimated mass of ~ 540 g (range 432 – 808 g) (Table S2). Similarly, the resulting estimated body mass for *A. darwini* (DDM 1577.730) was ~ 1200 g (range 914 – 1712 g) and ~ 1700 g for *A. archibaldi* (range 1488 – 2283 g) (Fig. S3, Table S2).

Formatted: Indent: First line: 0.5"

Formatted: Indent: First line: 0"

Phylogenetic Analysis Results

Both the Nelson strict consensus tree and the 50% majority rule tree place all three new taxa within enantiornithines, with *A. darwini* and MOR 3070 (*Avisaurus* sp.) resolved as part of the Avisauridae clade ~~in the 50% majority rule tree only~~ (Fig. 5).

FIGURE 5. Phylogenetic placement of new Hell Creek enantiornithines based on cladistic analysis. A) A subset of the strict consensus tree focusing on taxa around the Avisauridae, and B) the full majority tree. In the majority tree, the newly diagnosed Avisauridae family is comprised of six taxa, two of which are described in this publication. C) A 1.8 m tall human to scale with *A. darwini* (maroon), the largest known Early Cretaceous enantiornithine *Pengornis* (black), a medium-sized early enantiornithine, *Imparavis* (white), and finally, a minuscule enantiornithine, *Elektorornis* (smaller inset black). The extant *Buteo jamaicensis* (Red-tailed Hawk) is shown in grey.

Within the Avisauridae, *A. archibaldi* and *A. darwini* form a more exclusive clade supported by a metatarsal II that is approximately equal in distal extent to metatarsal IV [Character 236 1 → 0]. *Mirarce* and *Gettyia* form a clade supported by the absence of a plantarly excavated tarsometatarsus [Character 230 1 → 0], a *m. tibialis cranialis* tubercle located near or distal to the midpoint [Character 247 1 → 2], and the absence of proximoplantar fossae [Character 254 1 → 0]. These two clades form a polytomy with MOR 3070 and *Soroavisaurus*. The Avisauridae clade formed by these six OTU (operational taxonomic units) is supported by a tarsometatarsus whose midpoint width measures at least 20% that of its length (i.e., low aspect ratio) [Character 253 1 → 0], and a metatarsal II trochlea that is broader than that of metatarsal III [Character 238 0 → 1].

Magnusavis, *Neuquenornis*, and *Intiornis* are part of a larger polytomy outside of the Avisauridae along with *Mystiornis*, *Halimornis*, *Shenqiornis*, and *Elsornis*. With the removal of two previously identified avisaurids (*Neuquenornis*, and *Intiornis*), this family is now primarily defined not by the convex dorsal surface of metatarsal III or plantar excavation of the tarsometatarsus, characters that are

both widespread in enantiornithines (e.g., *Imparavis*, *Elektorornis*, *Qiliania*) (Ji et al., 2011; Xing et al., 2019; Wang et al., 2024), but instead by a proportionately robust tarsometatarsus (mediolateral width at the midpoint at least 20% that of the total proximodistal length). The removal of these two taxa differs from previous phylogenetic results which place them alongside avisaurids based on a plantar projection of the medial trochlea of metatarsal III (Atterholt et al., 2018). These results support interpretations that MOR 3070 is an avisaurid and likely a member of the genus *Avisaurus* (e.g., robust morphology, two proximoplantar fossae), and one that differs from other avisaurids; however, in light of the fragmentary remains available at present, we refrain from naming this specimen as a new taxon.

Formatted: Font color: Text 1

Discussion

Size Diversity in Late Cretaceous Enantiornithines

Here we describe three new large birds from the Maastrichtian Hell Creek Formation in Montana, all of which are placed in the clade Enantiornithes (Fig. 5). These three new enantiornithines not only increase the known taxonomic diversity of the Hell Creek avifauna, but they also add to existing evidence that members of this speciose clade achieved proportionally larger body sizes near the end of the Cretaceous.s.

Most enantiornithines described from Lower Cretaceous deposits, notably the Hauterivian to Aptian Lagerstätt[illegible] Spain and China (~ 130 – 120 Ma), and Upper Cretaceous amber specimens from Myanmar (~ 99 – –100 Ma), are on average similar in size- to extant sparrows (families Passeridae, Emberizidae) within the range of (~ 10 – 70 grams) and thrushes (family Turdidae) within the range of (~ 230 – 17 80 grams using the length of the humerus as a means of comparison) ((Chiappe and Walker, 2002; O'Connor et al., 2009; Tobias et al., 2022; Wang et al., 2024; Winkler et al., 2020c-e; Zhou, 2002). Currently, the smallest Upper Upper Cretaceous enantiornithines are Burmese amber specimens

(*Elektorornis*, approximately predicted to the size of 3 - 5 g extant trochilids based on hindlimb measurements) and the largest is being the holotype of *Pengornis houi* which is [similar in size to larger members within Ppsittaciformes within the range of (600-750 grams) (Billerman, 2020; Tobias et al., 2022; Zhou et al., 2008)]. Mass estimates for avisaurids reveal even larger Late Cretaceous enantiornithines with *Mirarce*, *A. darwini*, and *A. archibaldi* estimated as having body masses of ~ 540 g, ~ 1200 g, and ~ 1700 g respectively (see methods and results sections).

The tarsometatarsus scales with size in extant birds (Field et al., 2013) (Fig. S1). Extant birds with similar tarsometatarsal lengths to avisaurids are found among modern gulls (family Laridae) and hawks (family Acciptridae) whose masses average approximately 1500 grams (Billerman et al., 2022; Zeffer and Norberg, 2003). Field et al. (2013) found the greatest predictive power (highest R^2 scores) in determining mass of birds was the maximum diameter of the glenoid facet of the coracoid (maximum diameter of the coracoid's humeral articulation facet – HAF) and the length of the humerus, both which can be measured in the avisaurid *Mirarce* and suggests a body mass of 540 g in this taxon (range 432 – 808 g) (Atterholt et al., 2018). To estimate the mass of larger avisaurids known only from tarsometatarsi (*A. archibaldi* and *A. darwini*), the diameter of the coracoid glenoid and the length of the humerus were approximated by scaling them to *Mirarce* based on the tarsometatarsus, which measures 155% and 140% that of *Mirarce* in *A. archibaldi* and *A. darwini*, respectively. By averaging the masses of the nearest ten extant species to these measurements, the resulting estimated body mass for *A. archibaldi* equals ~ 1700 g (range 1488 – 2283 g), and ~1200 g for *A. darwini* (range 914 – 1712 g) (Fig. S2, Table S2).

The description of two new large-bodied avisaurids (*A. darwini*, *Avisaurus sp.*), and another proportionally large enantiornithine (*Magnusavis*), provides additional evidence in support of an observed trend in enantiornithines towards greater body size diversity over time (Atterholt et al., 2018; Chiappe, 1996; Chiappe and Walker, 2002; O'Connor, 2022) (Fig. 6). This increase in body size disparity towards the end Cretaceous is also observed in the Ornithuromorpha, the clade that includes Neornithes (Longrich et al., 2011). It has been suggested that larger body size among ornithuromorphs may have been a key contributing factor in surviving the end Cretaceous mass-extinction (Longrich et al., 2011; Torres et al.,

Formatted: Font: Italic
Formatted: Font: Italic
Formatted: Font: Italic
Formatted: Font: Italic
Formatted: Font: Italic

2021). Although depositional environments yielding bird fossils in Upper Cretaceous North American deposits are biased toward the preservation of larger organisms, this does not explain the apparent absence of larger enantiornithines in Lower Cretaceous Lagerstätten like the Jehol, which records larger birds like *Jeholornis* and *Sapeornis* but only small enantiornithines (Hu et al., 2023; Zhou and Zhang, 2003). Therefore, the signal indicating increased size diversity in the Late Cretaceous is likely a genuine one (Fig. 6). Fragmentary smaller-bodied enantiornithines have been recovered from the Late Cretaceous of North America, both in the Hell Creek Formation and the William's quarry site in Brazil (Longrich et al., 2011; Wu et al., 2021).

FIGURE 6. Regression analyses of A) tarsometatarsus length against time, and B) proportional tarsometatarsus mediolateral width against time. Both result in significant ($p < 0.01$) positive associations with an increase in body size over time. Avisaurids are colored red and all other enantiornithine are colored black.

In enantiornithines, both an increase in tarsometatarsus length and proportional mediolateral width (at the midpoint) are significantly positively associated with time ($p < 0.01$) (Fig. 6). Though well-preserved enantiornithine tarsometatarsal material from 119 – 80 Ma is limited, represented by two specimens from Myanmar preserved in amber (e.g., DIP V 19354, *Elektorornis*) (Xing et al., 2019, 2020), before 119 Ma the average tarsometatarsus length was 19 mm and the proportional mediolateral thickness was 12% (~ 2.28 mm) (aspect ratio of ~ 1:8). In contrast, these averages are 43 mm and 21% (mediolateral thickness of 9 mm) (aspect ratio 1:4 – 5) after ~ 80 Ma (Fig. 6). This pattern is likely exaggerated by the poor Upper Cretaceous fossil record and the fact no postcranial material has yet been described from William's Qquarry, although it is reportedly present in abundance (Wu et al., 2021).

Functional Morphology of the Avisaurid Tarsometatarsus

The Avisauridae are known almost entirely from tarsometatarsi. Members of the Avisauridae exhibit a combination of morphologies of the tarsometatarsus that are distinct from other enantiornithines and suggest raptorial behaviors including: the aspect ratio of the tarsometatarsus; the morphology and location of the *m. tibialis cranialis* tubercle; and the well-developed grooves of the trochleae. Specifically, these characteristics in avisaurids show striking similarities to strigids (owls, order Strigiformes) and accipitrids (hawks, eagles, order Accipitriformes). However, the differences between avisaurids and these extant clades indicates that neither provides a direct analogy for the ecological role occupied by avisaurids in the latest Cretaceous. The unique union of morphologies observed in avisaurids demonstrates that they likely occupied a predatorial ecological niche morphologically unrepresented in extant forms.

Aspect Ratio of the Tarsometatarsus

The morphology and topography of the tarsometatarsus greatly varies among extant birds, and can signal certain ecological behaviors such as wading, cursoriality, perching, prey grasping abilities, and pedal digit to substrate interaction (De Mendoza and Gómez, 2022; Grant, 1971; Madan et al., 2017; Tronrud, 2022; Volkov, 2004; Zeffer and Norberg, 2003a, b). When assessing the length and width of the tarsometatarsus of Mesozoic taxa, avisaurids overlap with a range of terrestrial nonvolant theropods (Fig. 7, Table S4). Thus, it is not surprising avisaurids were originally interpreted as non-avian, or as having reduced flight capabilities (Brett-Surman and Paul, 1985). However, when modern birds are added to the dataset, avisaurids cluster with larger birds like pelicans, owls, and hornbills weakening support for using the aspect ratio of the tarsometatarsus alone to predict volancy (Fig. 7).

FIGURE 7. Assessing aspect ratio of A) extinct avian and non-avian theropods, and then with the addition of B) extant volant birds. The grey dots represent extinct volant theropod genera. The red dots represent non-avian theropod genera. Blue dots represent extant avian genera. Refer to Table S4 for data.

~~The aspect ratio of the tarsometatarsus can be indicative of hindlimb function (Madan et al., 2017). Superficially, the proportions of avisaurid tarsometatarsi are similar to spheniscids (penguins) [*A. archibaldi*. *A. darwini*, *Mirarce*, and *Gettyia* ~ 1:4, *Pygoscelis adeliae* 1:2.5, *Spheniscus humboldti* 1:2, *Eudyptes chrysocome* 1:2, *Waimanu manneringi* (CM zfa 35) 1:5, *Kupoupou stilwelli* (NMNZ S.47312) 1:3, *Delphinornis larseni* (IB/P/B-0062) 1:3], whose tarsometatarsi are robust and proximodistally reduced (Bloklan et al., 2019; Jadwiszcak and Mörs, 2019; Slack et al., 2006). On land, the short hindlimbs of spheniscids facilitate a waddling gait, which is biomechanically efficient over long distances (Griffin and Kram, 2000). Though present in many other extant birds, notably spheniscid tarsometatarsi (particularly immature individuals) exhibit proportionally large lateral proximal vascular foramina. This structure may be functionally analogous to the intermetatarsal space between metatarsals III and IV in *A. darwini*, and *Soroavisaurus*. This space houses the lateral proximal intermetatarsal vein which eventually anastomoses with the common dorsal metatarsal vein on the dorsal side of the tarsometatarsus (Schwehn et al., 2024).~~

~~However, avisaurid tarsometatarsi are otherwise quite different from penguins, which lack a hypertrophied attachment for the *m. tibialis cranialis*, have much more well-developed intertrochlear space, and possess an unmodified, ginglymous metatarsal IV trochlea. Furthermore, evidence from *Mirarce* indicates that the overall skeletal anatomy is very different from spheniscids, lacking modified forelimbs and other morphologies associated with the specialized ecology of spheniscids.~~

Small bodied strigids (< 350 g) exhibit tarsometatarsi aspect ratios ~~even closer~~very similar to avisaurids (e.g., *Surnia ulula*, 1:4, *Aegolius funereus*, 1:5, *Glaucidium brasiliensis* 1:5) (Fig. 8). ~~Together with other features of the tarsometatarsus~~Similarities such as the aspect ratio of the tarsometatarsus, location of the *m. tibialis cranialis* tubercle, and the deeply notched trochlea of the metatarsal all ~~, this similarity~~ suggest~~s~~ extant raptorial birds, and more specifically ~~small~~ strigids, may be the best functional ~~analog~~analogues for understanding hindlimb function in avisaurids. Strigids are able to produce greater grip (i.e., constriction of pedal digits) force than other similar sized birds of prey (Fowler et al., 2009;

Formatted: Font: Italic

Formatted: Font: Italic

Formatted: Font: Italic

Tronrud, 2022; Ward et al., 2002). The proportionally ~~longer~~ more elongate tarsometatarsi of accipitrids (aspect ratios of 1:20 – 1:10) are better suited for rapid movements (i.e., grabbing, snatching), whereas the proportionally stouter tarsometatarsi of strigids are better equipped for forceful ~~grip and crushing~~ constriction (Tronrud, 2022; Ward et al., 2002). By lowering the aspect ratio of the tarsometatarsus relative to their tibiotarsus, strigids reduce the length between the fulcrum (ankle joint), moment of action (muscle tendon insertion), and the resistance force (the prey), ~~,~~ therefore reducing the required muscular force (i.e., torque) to lift or grip prey compared to longer-legged accipitrids (Vogel, 2013). In combination with having more mechanically efficient legs for producing ~~force~~dorsiflexing torque, strigids also possess mediolaterally wider muscle tendons and proportionally larger flexor muscles (e.g., *m. flexor digitorum longus*) than similarly sized accipitrids (Fowler et al., 2009; Tronrud, 2022; Volkov, 2004; Ward et al., 2002). The lower aspect ratio of the tarsometatarsus accommodates these wide muscle tendons, resulting in more applied force (i.e., contraction) over a given area. The *m. flexor digitorum longus*, which is responsible for contracting pedal digits II-IV, is the largest observable lower hindlimb muscle in strigids; the muscle belly, located on the caudal surface tibiotarsus, is so large that it also protrudes around the medial and lateral borders of the tibiotarsus (Volkov, 2004; Ward et al., 2002). The combination of potentially mediolaterally wider tendon insertions on the tarsometatarsus and greater ~~potential~~ force from increased flexor muscle mass in avisaurids may have resulted~~s~~ in comparatively more powerful and efficient pedal grip relative to other enantiornithines.

Formatted: Not Highlight

FIGURE 8. A) The left tarsometatarsi in dorsal and distal aspects with the *m. tibialis cranialis* tubercle highlighted in a) *Struthio camelus* (terrestrial ratite), b) *Turdus olivator* (arboreal passerine), c) *Cygnus buccinator* (semi-aquatic anseriform), d) *Buteo jamaicensis* (raptorial accipitrid), e) *Surnia ulula* (raptorial strigid), f) *Avisaurus darwini*. The distal view of each taxon is represented underneath the dorsal view to show variations of the intertrochlear grooves. Tarsometatarsi are not to scale. Reference photos of tarsometatarsi sourced from morphosource. B) Tarsometatarsus length (in meters) against the proportional distal location of the *m. tibialis cranialis* tubercle (TCT) in extant birds and enantiornithines,

including avisaurids (marked as asterisks). Extant birds are divided into their primary means of locomotion, following data from Zeffer and Norberg (2003).

Similarly, the tarsometatarsi of avisaurids exhibit lower aspects ratios than all other enantiornithines (with the exception of *Bauxitornis*), suggesting that members within this clade had ~~similarly~~ analogously powerful flexion of pedal digits ~~D~~II-IV (Dyke and [illegible]010). However, strigids are zygodactyl (with digits I and IV reversed), whereas avisaurids and accipitrids are anisodactyl (only digit I reversed). In contrast to strigids, accipitrids exhibit greater developed ~~have proportionally larger insertions of the~~ *m. flexor hallucis* ~~*hallicus*~~ *longus* tendons ~~-~~(Volkov, 2004). This ~~permits~~ facilitates greater force ~~given~~ to the contraction of the hallux ~~upon contraction~~ (and partially to digit II) ~~,~~which is necessary ~~for it to oppose~~as it opposes the combined forces generated by the three forward facing digits (II-IV) (Volkov, 2004; Ward et al., 2002). ~~-~~The mediolaterally wide and well-developed plantar excavation of the tarsometatarsus in large avisaurids (e.g., *Mirarce*, *Gettyia*, *Avisaurus*) is a space to accommodate mediolaterally expanded muscle tendons (of the *m. flexor hallucis longus* and *m. flexor digitorum longus*) as well as presence of a large *m. flexor hallucis brevis* belly (originating on the proximal part of the plantar surface), facilitating greater contractile grip. Avisaurids appear to unite the pedal arrangement of accipitrids and the mechanical benefits of the low aspect ratio tarsometatarsi in strigids.

Additionally, the well-developed, shelf-like labrum along the proximoplantar surface suggests the presence of an in-vivo cartilaginous hypotarsus, similar to that described in *Confuciusornis* (Jiang et al., 2017). If present, this feature would both aid in supporting the tendons of the digital flexor muscles and function as a moment arm for the ankle joint, allowing for greater tendon stability and ventroflexion of the lower hindlimb, respectively (Clifton and Carr, 2018; Mayr, 2016).

Functional Significance of the *M. Tibialis Cranialis* Tubercle

Formatted: Font: Not Bold

Formatted: Font: (Default) Times New Roman, Italic

Formatted: Not Highlight

Present in confuciusornithiforms, ornithuromorphs, and enantiornithines, the tubercle on metatarsal II has been hypothesized to represent the insertion point for the *m. tibialis cranialis* (Chiappe and Walker, 2002). This feature is particularly pronounced (dorsally projecting) in enantiornithines, present in nearly all taxa in which preservation allows observation. Interestingly, this feature is noticeably absent in non-avian theropods, and located on metatarsal III in a select number of Mesozoic birds (e.g., *Mystiornis*, *Confuciusornis*, *Ichthyornis*) (O'Connor et al., 2014). In extant birds, the *m. tibialis cranialis* originates on the proximocranial surface of the tibiotarsus and inserts on the dorsoproximal surface of the tarsometatarsus (Moreno and Carrascal, 1993; Zeffer and Norberg, 2003). As the muscle tendon dorsodistally extends from the distal tibiotarsus to the tarsometatarsus, it is secured in place by a retinaculum on the craniodistal surface of the tibiotarsus, just proximal to the ankle joint (Moreno and Carrascal, 1993; Zeffer and Norberg, 2003). Contraction of this muscle dorsiflexes the ankle, bringing the dorsal face of the tarsometatarsus towards the cranial surface of the tibiotarsus (Moreno and Carrascal, 1993; Zeffer and Norberg, 2003). The larger the insertion point (i.e., tubercle), the larger the corresponding muscle tendon, resulting in more powerful dorsiflexion. Likewise, as the insertion point migrates distally, it increases the in-lever of the system while the out-lever remains the same, increasing the system's mechanical advantage and ultimately allowing for increased dorsiflexion while carrying weight in the pes ~~Likewise, the more distal the insertion point, the greater the applied force relative to the fulcrum (ankle joint) to dorsiflex the ankle or compensate for the weight of an object held in the pes~~ (Gaslow, 1967; Moreno and Carrascal, 1993; Vogel, 2013) (Fig. 9).

FIGURE 9. A) Reconstruction of an articulated left tibiotarsus, tarsometatarsus, and pes of *Avisaurus darwini* with muscular reconstruction of the primary pedal flexors (caudal surface) and *m. tibialis cranialis* (cranial surface) in medial aspect. A) ankle joint shown in neutral position. B) ankle joint shown in dorsiflexion. ~~Tibiotarsus and pedal elements based on *Mirarce*, but scaled to appropriate size **(Atterholt et al., 2018).**~~ Pedal elements are based on *Mirarce* which preserves nearly all pedal phalanges (Atterholt et al., 2018). The tibiotarsus is based on the distal portion ~~).~~preserved in Mira[illegible]d the

Formatted: Font: (Default) Times New Roman
Formatted: Font: (Default) Times New Roman
Formatted: Font: (Default) Times New Roman
Formatted: Font: (Default) Times New Roman
Formatted: Font: Italic
Formatted: Font: Not Bold

reconstruction given in Atterholt et al. (2018). Muscles such as the gastrocnemius and extensors are not included to more clearly show the two major flexor muscles and the *m. tibialis cranialis*. The tibialis cranialis muscle attaches to the hypertrophied tubercle of metatarsal II. The action of the tibialis cranialis is flexed~~it is~~ to dorsiflex the ankle and invert the tarsometatarsus. This feature is hypertrophied in birds of prey compared to other extant birds, and is associated with an increased capacity to grasp prey with the pes. C) A member of the genus *Glaucidium* exhibiting the carrying strength that comes from having an enlarged and distally-migrated *m. tibialis cranialis* tubercle. Within this taxon, prey items may exceed three times the body mass of the owl (~~Billerman et al., 2022;~~ del Hoyo, 1999; Deshler, 2023). Photo used with permission and credited to Roy Priest.

Variation in the location of this muscle attachment among extant birds makes it a useful osteological correlate for determining interaction between substrate (or prey) and the pedes (Moreno and Carrascal, 1993; Zeffer and Norberg, 2003). Dorsiflexion is necessary in maintaining balance by lowering the center of mass when perching, which is why this feature is often more well-developed and proximally located in passerines than in terrestrial birds (Zeffer and Norberg, 2003), and similarly why this feature is well-developed in arboreal enantiornithines (Fig. 8). Extant birds that use their legs for swimming have even more prominent *m. tibialis cranialis* tubercles that are more distally located than perching birds. This facilitates dorsiflexion of the foot against the resistance of water, to subsequently extend and produce thrust (i.e., paddling). However, raptorial birds exhibit both the largest and most distally-located *m. tibialis cranialis* tubercles (Zeffer and Norberg, 2003) (Fig. 8). Similar to the relationship between raptorial and non-raptorial birds, avisaurid *m. tibialis cranialis* tubercles differ from other enantiornithines in being larger and more distally located (Fig. 8; Table S3).

Variation in both the size and distal location of this muscle attachment among raptorial birds reflects differences in acquisition and proportional size of prey (Volkov, 2004; Zeffer and Norberg, 2003). Along with the size and distal location of the tubercle, the relative medial location on the tarsometatarsus also affects the degree of pedal inversion that occurs when the *m. tibialis cranialis* is

contracted (Fig. 10). Inversion of the pes while grasping prey increases grip stability as the force being generated pushes against the opposing foot. The more medial the location of the tubercle, the greater the degree of pedal inversion (Volkov, 2004; Zeffer and Norberg, 2003). Compared to accipitrids and falconids, strigids also have the most distomedially located *m. tibialis cranialis* tubercles. In particular, smaller bodied owls, like *Glaucidium* tend to exhibit greater distomedial migration of the attachment compared to larger strigids, further aiding in supporting proportionally larger prey when in flight. .For example, *Glaucidium* *gnoma* (Pygmy owls), though diminutive (up to ~ 8080 g) will subdue and travel with prey items that often rival or exceed their own body mass (e.g., *Microtus* sp., 60 g; *Coccothraustes* sp., 60 g; *Callipepla sp.,* 160 g) (Billerman et al., 2022Deshler, 2023; Zeffer and Norberg 2003) (Fig. 9B).

Formatted: Indent: First line: 0"

FIGURE 10. Action of the tibialis cranialis muscle on the ankle joint, shown in a A) resting position and a B) contracted position. As the muscle contracts, not only does the ankle dorsiflex, bringing the tibiotarsus and tarsometatarsus closer together, but the tarsometatarsus and phalanges invert (i.e., elevation of the medial border of the foot).

The *m. tibialis cranialis* tubercle in avisaurids is larger and more distomedially located on the tarsometatarsus than in any Mesozoic bird, non-avian theropod, and nearly every extant bird (Brett-Surman and Paul, 1985). The presence of the large distomedially located *m. tibialis cranialis* tubercle in avisaurids, together with the lower aspect ratio of the tarsometatarsus, suggests a functional similarity with extant birds of prey that distinguishes them from other enantiornithines. Based on the numerous shared morphologies with strigids, avisaurid tarsometatarsi were likely adapted for strong dorsiflexion and carrying proportionally large prey items while volant (Fig. 11). However, it should be noted that the largest avisaurids are estimated to be nearly twenty times larger than *G. gnoma*, (*G. gnoma* 80 g, *A. archibaldi* ~ 1700 g) which limits the proportional size of prey that could likely be lifted given that as an

Formatted: Font: Italic

Formatted: Font: Italic

Formatted: Font: Italic

Formatted: Font: Italic

object’s surface area increases, its volume increases at a greater rate (i.e., the square-cube law). Carrying similar proportionally-sized prey as some strigids (e.g., *Surnia*, *Glaucidium*, *Aegolius*) may have been more plausible for smaller-bodied avisaurids like *Gettyia* (Zeffer and Norberg, 200). Among avisaurids, *Gettyia* (Fig. 4D) exhibits the proportionally distal-most *m. tibialis cranialis* tubercle. It is also the smallest known avisaurid (~ 540 g), with a tarsometatarsal length that is less than half that of *A. darwini archibaldi* (~1700 g) 30.9 mm, *A. archibaldi* 73.9 mm) (Atterholt et al., 2018) (Fig. 8A). This suggests that like diminutive strigids, *Gettyia* was adapted for subduing and carrying proportionally large prey (e.g., *Surnia*, *Glaucidium*, *Aegolius*) (Zeffer and Norberg, 2003).

FIGURE 11. Reconstruction of an avisaurid (e.g., *A. darwini*). Morphology of the tarsometatarsus suggests that these large birds engaged in raptorial behavior and could carry proportionally large prey. Illustration done by Ville Sinkkonen.

Significance of Trochlear Groove Development

Strigids Birds which rely on increased constriction of prey (e.g., birds of prey) exhibit well-developed and deeply-grooved metatarsal trochleae, which correspond to both proportionally longer and more robust pedal digits than similarly sized accipitrids (Einoder and Richardson, 2007; Gianechini et al., 2020; Madan et al., 2017; Ward et al., 2002) (Fig. 8). This grooved morphology exhibits distinctly developed excavations between the medial and lateral condyles. The deeply-grooved metatarsal trochlea corresponds to phalanges with greater-projecting intercotylar crests, and excavated articular cotyla. This morphology results in a larger articular surface between the proximal phalanges and the tarsometatarsus, resulting in a restricted range of motion at the metatarsophalangeal joint when the flexor tendons contract, (i.e., gripping an object) (Madan et al., 2017; Tronrud, 2022; Usherwood et al., 2014). Pedal digits II-IV of strigids have mediolaterally wider tendons of the *m. flexor digitorum* than in accipitrids, facilitating stronger contraction and therefore grip strength (Madan et al., 2017; Usherwood et al., 2014). Along

Formatted: Font: Italic

Formatted: Font: Italic

Together with the low aspect ratio of the tarsometatarsus, the more robust pedal digits of strigids ~~are~~ are suggested to also aid in ~~crushing~~ constricting prey ~~and importantly, securing proportionally large prey in conditions where visual acuity is low (i.e., low light)~~ (Fowler et al., 2009; Goslow, 1967, 1972; Madan et al., 2017; Tronrud, 2022). ~~Unlike diurnal accipitrids, pursuit of prey in low light conditions makes recapture unlikely should prey escape upon initial attack, making lethal first contact imperative (Goslow, 1967, 1972; Madan et al., 2017).~~ This may explain why strigiforms have been found to apply more ~~crushing~~ constrictive force than is necessary to kill their prey items (Madan et al., 2017; Usherwood et al., 2014) Well-developed (compared to other enantiornithines) trochlear grooves are present in the mediolaterally wide trochleae of metatarsals II and III of *A. archibaldi*, *A. darwini*, *Mirarce*, *Soroavisaurus*, and *Gettyia*, suggesting similarly robust and powerful pedal digits, reminiscent of those seen in extant birds of prey (Atterholt et al., 2018; Brett-Surman & Paul, 1985; Einoder and Richardson, 2007; Madan et al., 2017; Tronrud, 2022). Together with the absolute larger size of avisaurids compared to other enantiornithines, a proportionally high level of applied pedal digit pressure (i.e., ~~grip~~constriction) is plausible for members of this clade.

Conclusion

Here we describe three new enantiornithines, two of which are new avisaurids, all from exposures of the latest Cretaceous Hell Creek Formation in Montana. Both new avisaurids are larger than the largest Early Cretaceous enantiornithine. This supports the apparent trend towards increased body mass diversity in the Late Cretaceous among enantiornithines with large-bodied forms being most heavily represented in the Late Cretaceous fossil record. By increasing body size, this unique bird lineage explored new ecospaces suggesting that enantiornithines were still diversifying near the end Cretaceous. The morphology of the avisaurid tarsometatarsus suggests some Late Cretaceous enantiornithines adopted raptorial ecologies. Avisaurid tarsometatarsi exhibit low aspect ratios, proportionally large and

distomedially located *m. tibialis cranialis* tubercles, and well-developed grooved metatarsal trochleae. All of these morphologies are uniquely found in members of extant raptorial birds, specifically strigids and accipitrids, and suggest similar powerful pedal constriction behaviors in an anisodactyl pedal formation. By combining multiple aspects of both the tarsometatarsus of both extant accipitrids and strigids, , avisaurids represent a group of enantiornithines which employed raptorial behaviors that lack modern morphological analogues do not exist.

Supporting Information

FIGURE S1. Analysis of 863 extant birds (317 species) measuring mass in grams against length of the tarsometatarsus. Data from Field et al., (2013).

FIGURE S2. Close-up photos of the proximoplantar fossa on the plantar face of (A) *Avisaurus darwini*, and (B) *Avisaurus* sp. MOR 3070. The depressions are indicated by the black arrows.

Formatted: Font: Italic

Formatted: Font: Italic

Formatted: Font: Italic

FIGURE S32. Plotting three avisaurids to approximate mass. Utilizing the dataset from Field et al., (2013), three avisaurids (*A. archibaldi, A. darwini, Mirarce*) were plotted against (A) all sampled extant birds, and then against all birds, (B) by family. Once closely associated families were identified, an averaged mass estimate could be calculated using data collected within Field et al., (2013).

TABLE S1. Sampled enantiornithine tarsometatarsi length and width at the midpoint (mm).

TABLE S2. Field et al. (2013) measurements used for weight estimations in extant birds and predictive masses for selected avisaurids based on averaged results.

TABLE S3. Dataset from Zeffer and Norberg (2003) with added enantiornithines to assess distal location of the *m. tibialis cranialis* tubercle.

TABLE S4. Dataset used for comparative aspect ratios of the tarsometatarsus between avian and non-avian theropods. All extant taxa used are from Field et al., 2013.

Acknowledgements

Thank you to the editor and to both reviewers for their constructive suggestions for our manuscript. We would like to acknowledge the field crews of Nathan Carroll (Carter County Museum), Thomas Carr (Dinosaur Discovery Museum), and Frankie Jackson (Montana State University). Without their effort, these new taxa would not have been found. Thank you to Daigo Yamamura for providing info on the collection of MOR 3070. Thank you to the Bureau of Land Management and Montana Fish, Wildlife & Parks for providing access to lands where these discoveries were made. Thank you to Samantha Clark who illustrated the tarsometatarsi in figures 1 – 3, and to Ville Sinkkonen who illustrated figure 11. Thank you to Akiko Shinya for assistance in additional preparation of the fossils.

References

Abourachid, A., Fabre, A., Cornette, R., Höfling, E., 2017. Foot shape in arboreal birds: two morphological patterns for the same pincer‐like tool. Journal of Anatomy 231, 1‐11. https://doi.org/10.1111/joa.12614

Atterholt, J., Hutchison, J.H., O’Connor, J.K., 2018. The most complete enantiornithine from North America and a phylogenetic analysis of the Avisauridae. PeerJ 6, e5910. https://doi.org/10.7717/peerj.5910

Bailleul, A.M., O'Connor, J., Zhang, S., Li, Z., Wang, Q., Lamanna, M.C., Zhu, X., Zhou, Z., 2019. An Early Cretaceous enantiornithine (Aves) preserving an unlaid egg and probable medullary bone. Nat Commun 10, 1275. https://doi.org/10.1038/s41467-019-09259-x

Baumel, J.J., Witmer , L.M., 1993. Osteologia, in: Handbook or Avian Anatomy. Nuptial Ornithological Club, Cambridge, Massachusetts.

Biewener, A.A., 2011. Muscle function in avian flight: achieving power and control. Phil. Trans. R. Soc. B 366, 1496–1506. https://doi.org/10.1098/rstb.2010.0353

Billerman, S. M. 2020. Old World Parrots (Psittaculidae), version 1.0. In Birds of the World (S. M. Billerman, B. K. Keeney, P. G. Rodewald, and T. S. Schulenberg, Editors). Cornell Lab of Ornithology, Ithaca, NY, USA. https://doi.org/10.2173/bow.psitta4.01

~~Billerman, S.M., Keeney, B.K., Rodewald, P.G., Schulenberg, T.S., 2022. Birds of the World. Cornell Lab of Ornithology, Ithaca, NY, USA.~~

~~Blokland, J., Reid, C., Worthy, T., Tennyson, A., Clarke, J., Scofield, R., 2019. Chatham Island Paleocene fossils provide insight into the palaeobiology, evolution, and diversity of early penguins (Aves, Sphenisciformes). Palaeontol Electron. https://doi.org/10.26879/1009~~

Brett-Surman, M.K., Paul, G.S., 1985. A new family of bird-like dinosaurs linking Laurasia and Gondwanaland. Journal of Vertebrate Paleontology 5, 133–138. https://doi.org/10.1080/02724634.1985.10011851

Buscalioni, A.D., Poyato-ariza, F.J., 2016. Las Hoyas: a unique Cretaceous ecosystem. New Mexico Museum of Natural History and Science Bulletin.

Cau, A., Arduini, P., 2008. *Enantiophoenix electrophyla* gen. et sp. nov. (Aves, Enantiornithes) from the Upper Cretaceous (Cenomanian) of Lebanon and its phylogenetic relationships. Atti Soc. it. Sci. nat. Museo civ. Stor. nat. Milano 149, 293–324.

Chiappe, L.M., Navalón, G., Martinelli, A.G., Nava, W., Field, D.J., 2022. Fossil basicranium clarifies the origin of the avian central nervous system and inner ear. Proc. R. Soc. B. 289, 20221398. https://doi.org/10.1098/rspb.2022.1398

Formatted: Font: Italic

Formatted: Font: Italic

Chiappe, L., 1993. Enantiornithine (Aves) Tarsometatarsi from the Cretaceous Lecho Formation of Northwestern Argentina. American Museum Novitates 3083.

Chiappe, L.M., 1992. Enantiornithine (Aves) tarsometatarsi and the avian affinities of the Late Cretaceous Avisauridae. Journal of Vertebrate Paleontology 12, 344–350. https://doi.org/10.1080/02724634.1992.10011464

Chiappe, L.M., Calvo, J.O., 1994. *Neuquenornis volans*, a new Late Cretaceous bird (Enantiornithes: Avisauridae) from Patagonia, Argentina. Journal of Vertebrate Paleontology 14, 230–246. https://doi.org/10.1080/02724634.1994.10011554

Chiappe, L.M., Walker, C.A., 2002. Skeletal morphology and systematics of the Cretaceous Euenantiornithines (Ornithothoraces: Enantiornithes)., in: Mesozoic Birds: Above the Heads of Dinosaurs. University of California Press, Berkley, CA, p. pp 240-267.

Clifton, G.T., Carr, J.A., Biewener, A.A., 2018. Comparative hindlimb myology of foot-propelled swimming birds. Journal of Anatomy 232, 105–123. https://doi.org/10.1111/joa.12710

del Hoyo, J., Elliot, A., Sargatal, J., Collar, N.J., 1999. Handbook of the birds of the world. Volume 5. Lynx edicions, Barcelona.

De Mendoza, R.S., Gómez, R.O., 2022. Ecomorphology of the tarsometatarsus of waterfowl (Anseriformes) based on geometric morphometrics and its application to fossils. The Anatomical Record 305, 3243–3253. https://doi.org/10.1002/ar.24891

Deshler, J. F. 2023. Northern Pygmy-Owl (Glaucidium gnoma), version 2.0. In Birds of the World (P. G. Rodewald, Editor). Cornell Lab of Ornithology, Ithaca, NY, USA. https://doi.org/10.2173/bow.nopowl.02

Dyke, G.J., Ősi, A., 2010. A review of Late Cretaceous fossil birds from Hungary. Geological Journal 45, 434–444. https://doi.org/10.1002/gj.1209

Einoder, L.D., Richardson, A.M.M., 2007. Aspects of the h~~H~~indlimb m~~M~~orphology of s~~S~~ome a~~A~~ustralian b~~B~~irds of p~~P~~rey: a c~~A C~~omparative and q~~Q~~uantitative s~~S~~tudy. The Auk 124, 773–788. https://doi.org/10.1093/auk/124.3.773

Field, D.J., Lynner, C., Brown, C., Darroch, S.A.F., 2013. Skeletal Correlates for Body Mass Estimation in Modern and Fossil Flying Birds. PLoS ONE 8, e82000. https://doi.org/10.1371/journal.pone.0082000

Fowler, D.W., Freedman, E.A., Scannella, J.B., 2009. Predatory f~~F~~unctional m~~M~~orphology in r~~R~~aptors: i~~I~~nterdigital v~~V~~ariation in t~~T~~alon s~~S~~ize is r~~ Is R~~elated to p~~P~~rey r~~R~~estraint and i~~I~~mmobilisation t~~T~~echnique. PLoS ONE 4, e7999. https://doi.org/10.1371/journal.pone.0007999

Fowler, D., 2020. The Hell Creek Formation, Montana: A stratigraphic review and revision based on a sequence stratigraphic approach. Geosciences, 10(11), p.435.

Fregenal-Martínez, M., Nieves Meléndez, N., Muñoz-García, M.B., Elez, J. and Horra, R.D.L., 2017. The stratigraphic record of the Late Jurassic-Early Cretaceous rifting in the Alto Tajo-Serranía de Cuenca region (Iberian Ranges, Spain).

Gianechini, F.A., Ercoli, M.D. and Díaz-Martínez, I., 2020. Differential locomotor and predatory strategies of Gondwanan and derived Laurasian dromaeosaurids (Dinosauria, Theropoda, Paraves): Inferences from morphometric and comparative anatomical studies. Journal of Anatomy, 236(5), pp.772-797.

Goslow, G.E., 1972. Adaptive Mechanisms of the Raptor Pelvic Limb. The Auk 89, 47–64. https://doi.org/10.2307/4084059

Goslow, G.E., 1967. Functional analysis of the striking mechanisms of raptorial birds. ~~FUNCTIONAL ANALYSIS OF THE STRIKING MECHANISMS OF RAPTORIAL BIRDS.~~ University of California Davis.

Goloboff, P.A., Farris, J.S., Nixon, K.C., 2008. TNT, a free program for phylogenetic analysis. Cladistics 24, 774–786. https://doi.org/10.1111/j.1096-0031.2008.00217.x

Grant, P.R., 1971. Variation in the t~~T~~arsus l~~L~~ength of b~~B~~irds in i~~I~~sland and m~~M~~ainland r~~R~~egions. Evolution 25, 599. https://doi.org/10.2307/2406943

~~Griffin, T.M., Kram, R., 2000. Penguin waddling is not wasteful. Nature 408, 929–929. https://doi.org/10.1038/35050167~~

Höfling, E., Abourachid, A., 2021. The skin of birds' feet: Morphological adaptations of the plantar surface. Journal of Morphology 282, 88–97. https://doi.org/10.1002/jmor.21284

Hone, D.W.E., Dyke, G.J., Haden, M., Benton, M.J., 2008. Body size evolution in Mesozoic birds. J of Evolutionary Biology 21, 618–624. https://doi.org/10.1111/j.1420-9101.2007.01483.x

Hu, H., Wang, Y., Fabbri, M., O'Connor, J.K., McDonald, P.G., Wroe, S., Yin, X., Zheng, X., Zhou, Z., Benson, R.B.J., 2023. Cranial osteology and palaeobiology of the Early Cretaceous bird *Jeholornis prima* (Aves: Jeholornithiformes). Zoological Journal of the Linnean Society 198, 93–112. https://doi.org/10.1093/zoolinnean/zlac089

~~Jadwiszczak, P., Mörs, T., 2019. First partial skeleton of Delphinornis larseni Wiman, 1905, a slender-footed penguin from the Eocene of Antarctic Peninsula. Palaeontol Electron. https://doi.org/10.26879/933~~

Jadwiszczak, P., 2015. Another look at tarsometatarsi of early penguins. Polish Polar Research 36, 343–354. https://doi.org/10.1515/popore-2015-0024

Jiang, B., Zhao, T., Regnault, S., Edwards, N.P., Kohn, S.C., Li, Z., Wogelius, R.A., Benton, M.J., Hutchinson, J.R., 2017. Cellular preservation of musculoskeletal specializations in the Cretaceous bird *Confuciusornis*. Nat Commun 8, 14779. https://doi.org/10.1038/ncomms14779

Longrich, N.R., Tokaryk, T., Field, D.J., 2011. Mass extinction of birds at the Cretaceous–Paleogene (K–Pg) boundary. Proc. Natl. Acad. Sci. U.S.A. 108, 15253–15257. https://doi.org/10.1073/pnas.1110395108

Madan, M.A., Rayfield, E.J., Bright, J.A., 2017. Scaling and functional morphology in strigiform hind limbs. Sci Rep 7, 44920. https://doi.org/10.1038/srep44920

Martin, E.M., Sherratt, E., 2023. Grasping hold of functional trade-offs using the diversity of foot forms in Australian birds. Evol Ecol 37, 945–959. https://doi.org/10.1007/s10682-023-10261-5

Mayr, G., 2016. Variations in the hypotarsus morphology of birds and their evolutionary significance. Acta Zoologica 97, 196–210. https://doi.org/10.1111/azo.12117

Formatted: Font: Italic

Formatted: Font: Italic

Formatted: Font: Italic

Mohamed, R., 2019. Histomorphological study on the tongue of the duck in the Caribbean with relation to feeding habit and #8195; J Adv Vet Anim Res 74. https://doi.org/10.5455/javar.2019.f315

Moreno, E., Carrascal, L.M., 1993. Leg m~~M~~orphology and f~~F~~eeding p~~P~~ostures in f~~F~~our *P~~P~~arus* s~~S~~pecies: a ~~A~~n e~~E~~xperimental e~~E~~comorphological a~~A~~pproach. Ecology 74, 2037–2044. https://doi.org/10.2307/1940849

Novas, F.E., Agnolín, F.L., Scanferla, C.A., 2010. New enantiornithine bird (Aves, Ornithothoraces) from the Late Cretaceous of NW Argentina. Comptes Rendus Palevol 9, 499–503. https://doi.org/10.1016/j.crpv.2010.09.005

O'Connor, J.K., Chiappe, L.M., 2011. A revision of enantiornithine (Aves: Ornithothoraces) skull morphology. Journal of Systematic Palaeontology 9, 135–157. https://doi.org/10.1080/14772019.2010.526639

O'Connor, J., 2022. Enantiornithes. Current Biology 32, R1166–R1172. https://doi.org/10.1016/j.cub.2022.07.074

O'Connor, J.K., Wang, X., Chiappe, L.M., Gao, C., Meng, Q., Cheng, X., Liu, J., 200~~9~~9~~a~~. Phylogenetic support for a specialized clade of Cretaceous enantiornithine birds with information from a new species. Journal of Vertebrate Paleontology 29, 188–204. https://doi.org/10.1080/02724634.2009.10010371

~~O'Connor, J.K., Wang, X., Chiappe, L.M., Gao, C., Meng, Q., Cheng, X., Liu, J., 2009b. Phylogenetic support for a specialized clade of Cretaceous enantiornithine birds with information from a new species. Journal of Vertebrate Paleontology 29, 188–204. https://doi.org/10.1080/02724634.2009.10010371~~

O'Connor, J.K., Clark. , A.D., Herrera, F., Zhou, Z., Xiaoli , W., Zheng, X., 2023~~b~~. *Longipteryx* stomach contents indicate that morphology does not accurately predict diet in enantiornithines ~~LONGIPTERYX STOMACH CONTENTS INDICATE THAT MORPHOLOGY DOES NOT ACCURATELY PREDICT DIET IN ENANTIORNITHINES~~. Cincinnati, OH

Formatted: Font: Italic

Formatted: Font: Italic

O'Connor, J.K., Averianov, A.O., Zelenkov, N.V., 2014. A confuciusornithiforms from the Early Cretaceous of Siberia and a discussion of the evolution of avian hindlimb musculatureA CONFUCIUSORNITHIFORM (AVES, PYGOSTYLIA)-LIKE TARSOMETATARSUS FROM THE EARLY CRETACEOUS OF SIBERIA AND A DISCUSSION OF THE EVOLUTION OF AVIAN HIND LIMB MUSCULATURE. Journal of Vertebrate Paleontology 34.

O'Connor, J.K., Li, D.-Q., Lamanna, M.C., Wang, M., Harris, J.D., Atterholt, J., You, H.-L., 2016. A new Early Cretaceous enantiornithine (Aves, Ornithothoraces) from northwestern China with elaborate tail ornamentation. Journal of Vertebrate Paleontology 36, e1054035. https://doi.org/10.1080/02724634.2015.1054035 Schneider, C. A., Rasband, W. S., & Eliceiri, K. W. 2012. NIH Image to ImageJ: 25 years of image analysis. Nature Methods, 9(7), 671–675. doi:10.1038/nmeth.2089

Schwehn, R., Engelke, E., Seiler, C., Fischer, D., Seifert, H., Pfarrer, C., Fehr, M., Legler, M., 2024. Blood Vessel Topography of the Feet in Selected Species of Birds of Prey and Owls. Veterinary Sciences 11, 88. https://doi.org/10.3390/vetsci11020088

Slack, K.E., Jones, C.M., Ando, T., Harrison, G.L. (Abby), Fordyce, R.E., Arnason, U., Penny, D., 2006. Early Penguin Fossils, Plus Mitochondrial Genomes, Calibrate Avian Evolution. Molecular Biology and Evolution 23, 1144–1155. Tobias, J.A., Sheard, C., Pigot, A.L., Devenish, A.J., Yang, J., Sayol, F., Neate-Clegg, M.H., Alioravainen, N., Weeks, T.L., Barber, R.A. and Walkden, P.A., 2022. AVONET: morphological, ecological and geographical data for all birds. Ecology Letters, 25(3), pp.581-597.

Torres, C.R., Norell, M.A., Clarke, J.A., 2021. Bird neurocranial and body mass evolution across the end-Cretaceous mass extinction: The avian brain shape left other dinosaurs behind. Sci. Adv. 7, eabg7099. https://doi.org/10.1126/sciadv.abg7099

Tronrud, B., 2022. Evolution and ecomorphology of the avian foot: the morphology of the tarsometatarsus in birds and its responses to evolutionary changes in foot use. University of Oxford.

Usherwood, J.R., Sparkes, E.L., Weller, R., 2014. Leap and Strike kinetics of an acoustically “hunting” barn owl *Tyto alba*. Journal of Experimental Biology jeb.107169. https://doi.org/10.1242/jeb.107169

Varricchio, D.J., Chiappe, L.M., 1995. A n~~N~~ew e~~E~~nantiornithine b~~B~~ird f~~F~~rom the U~~U~~pper C~~C~~retaceous t~~T~~wo m~~M~~edicine f~~F~~ormation of Montana. Journal of Vertebrate Paleontology 15, 201–204. https://doi.org/10.1080/02724634.1995.10011219

Vogel, S., 2013. Comparative biomechanics: life’s physical world, Second edition. ed. Princeton University Press, Princeton.

Volkov, S.V., 2004. The hindlimb musculature of the true owls (Strigidae: Strigiformes): morphological peculiarities and general adaptations ~~THE HINDLIMB MUSCULATURE OF THE TRUE OWLS (STRIGIDAE: STRIGIFORMES): MORPHOLOGICAL PECULIARITIES AND GENERAL ADAPTATIONS~~. Ornithologia 31.

Walker, C.A., 1981. New subclass of birds from the Cretaceous of South America. Nature 292, 51–53. https://doi.org/10.1038/292051a0

Wang, X., Clark, A.D., O’Connor, J.K., Zhang, X., Wang, X., Zheng, X., Zhou, Z. 2024. First edentulous enantiornithines (Aves: Ornithothoraces) from the Lower Cretaceous Jehol Avifauna. Cretaceous Research.

Ward, A.B., Weigl, P.D., Conroy, R.M., 2002. Functional m~~M~~orphology of r~~R~~aptor h~~H~~indlimbs: i~~I~~mplications for r~~R~~esource p~~P~~artitioning. The Auk 119, 1052–1063. https://doi.org/10.1093/auk/119.4.1052

Winkler, D. W., S. M. Billerman, and I. J. Lovette. 2020a. Hawks, Eagles, and Kites (Accipitridae), version 1.0. In Birds of the World (S. M. Billerman, B. K. Keeney, P. G. Rodewald, and T. S. Schulenberg, Editors). Cornell Lab of Ornithology, Ithaca, NY, USA. https://doi.org/10.2173/bow.accipi1.01

Winkler, D. W., S. M. Billerman, and I. J. Lovette. 2020b. Gulls, Terns, and Skimmers (Laridae), version 1.0. In Birds of the World (S. M. Billerman, B. K. Keeney, P. G. Rodewald, and T. S.

Formatted: Font: Italic

Formatted: Font: Italic

Schulenberg, Editors). Cornell Lab of Ornithology, Ithaca, NY, USA. https://doi.org/10.2173/bow.larida1.01

Winkler, D. W., S. M. Billerman, and I. J. Lovette. 2020c. Old World Buntings (Emberizidae), version 1.0. In Birds of the World (S. M. Billerman, B. K. Keeney, P. G. Rodewald, and T. S. Schulenberg, Editors). Cornell Lab of Ornithology, Ithaca, NY, USA. https://doi.org/10.2173/bow.emberi2.01

Winkler, D. W., S. M. Billerman, and I. J. Lovette. 2020d. Old World Sparrows (Passeridae), version 1.0. In Birds of the World (S. M. Billerman, B. K. Keeney, P. G. Rodewald, and T. S. Schulenberg, Editors). Cornell Lab of Ornithology, Ithaca, NY, USA. https://doi.org/10.2173/bow.passer4.01

Winkler, D. W., S. M. Billerman, and I. J. Lovette. 2020e. Thrushes and Allies (Turdidae), version 1.0. In Birds of the World (S. M. Billerman, B. K. Keeney, P. G. Rodewald, and T. S. Schulenberg, Editors). Cornell Lab of Ornithology, Ithaca, NY, USA. https://doi.org/10.2173/bow.turdid1.01

Wu, Y.-H., Chiappe, L.M., Bottjer, D.J., Nava, W., Martinelli, A.G., 2021. Dental replacement in Mesozoic birds: evidence from newly discovered Brazilian enantiornithines. Sci Rep 11, 19349. https://doi.org/10.1038/s41598-021-98335-8

Xing, L., O'Connor, J.K., Chiappe, L.M., McKellar, R.C., Carroll, N., Hu, H., Bai, M., Lei, F., 2019. A n~~N~~ew e~~E~~nantiornithine b~~B~~ird with u~~U~~nusual p~~P~~edal p~~P~~roportions f~~F~~ound in a~~A~~mber. Current Biology 29, 2396-2401.e2. https://doi.org/10.1016/j.cub.2019.05.077

Xing, L., Cockx, P., O'Connor, J.K., Mckellar, R.C., 2020. A newly discovered enantiornithine foot preserved in mid-Cretaceous Burmese amber. PE 3, 212–219. https://doi.org/10.11646/palaeoentomology.3.2.11

Xu, X., Zhou, Z., Wang, Y., Wang, M., 2020. Study on the Jehol Biota: Recent advances and future prospects. Sci. China Earth Sci. 63, 757–773. https://doi.org/10.1007/s11430-019-9509-3

Yamamura, Daigo. Effects of depositional environment, asssemblage taphonomy and diagenesis on vertebrate skeletal preservation in a crevasse splay sandstone, upper Cretaceous Hell Creek

Formation, eastern Montana. Diss. Montana State University-Bozeman, College of Letters & Science, 2013.

Yu, Z., Wang, M., Li, Y., Deng, C. and He, H., 2021. New geochronological constraints for the Lower Cretaceous Jiufotang Formation in Jianchang Basin, NE China, and their implications for the late Jehol Biota. Palaeogeography, Palaeoclimatology, Palaeoecology, 583, p.110657.

Zeffer, A., Norberg, U.M.L., 2003a. Leg morphology and locomotion in birds: requirements for force and speed during ankle flexion. Journal of Experimental Biology 206, 1085–1097. https://doi.org/10.1242/jeb.00208

Zeffer, A., Johansson, L.C. and Marmebro, Å., 2003b. Functional correlation between habitat use and leg morphology in birds (Aves). Biological Journal of the Linnean Society, 79(3), pp.461-484.

Zheng, X., O'Connor, J.K., Huchzermeyer, F., Wang, X., Wang, Y., Zhang, X., Zhou, Z., 2014. New s~~S~~pecimens of *Yanornis* i~~I~~ndicate a p~~P~~iscivorous d~~D~~iet and m~~M~~odern a~~A~~limentary c~~C~~anal. PLoS ONE 9, e95036. https://doi.org/10.1371/journal.pone.0095036

Zhong, Y., Huyskens, M.H., Yin, Q.Z., Wang, Y., Ma, Q. and Xu, Y.G., 2021. High-precision geochronological constraints on the duration of 'Dinosaur Pompeii'and the Yixian Formation. National Science Review, 8(6), p.nwab063.

Zhou, Z., 2002. A new and primitive enantiornithine bird from the Early Cretaceous of China. Journal of Vertebrate Paleontology 22, 49–57. https://doi.org/10.1671/0272-4634(2002)022[0049:ANAPEB]2.0.CO;2

Zhou, Z., Clarke, J., Zhang, F., 2008. Insight into diversity, body size and morphological evolution from the largest Early Cretaceous enantiornithine bird. Journal of Anatomy 212, 565–577. https://doi.org/10.1111/j.1469-7580.2008.00880.x

Zhou, Z., Zhang, F., 2003. Anatomy of the primitive bird *Sapeornis chaoyangensis* from the Early Cretaceous of Liaoning, China. Can. J. Earth Sci. 40, 731–747. https://doi.org/10.1139/e03-011

Zhou, Z., Zhang, F., 2006. A beaked basal ornithurine bird (Aves, Ornithurae) from the Lower Cretaceous of China. Zoologica Scripta 35, 363–373. https://doi.org/10.1111/j.1463-6409.2006.00234.x

Formatted: Font: (Default) Times New Roman

Formatted: Font: Italic

Formatted: Font: Italic

Formatted: Font: Italic

1063

We would like to thank the editor, and both reviewers for their very constructive and helpful suggestions for our manuscript. We find in having incorporated them and making other suggested changes, that we have made our manuscript more robust and clearer concerning our conclusions and lines of reasoning. Thank you for your contributions to our manuscript.

Sincerely,
Alex Clark (corresponding author)

Editor

1. Please ensure that your manuscript meets PLOS ONE's style requirements, including those for file naming. The PLOS ONE style templates can be found at https://journals.plos.org/plosone/s/file?id=wjVg/PLOSOne_formatting_sample_main_body.pdf and https://journals.plos.org/plosone/s/file?id=ba62/PLOSOne_formatting_sample_title_authors_affiliations.pdf

Completed

2. In your manuscript, please provide additional information regarding the specimens used in your study. Ensure that you have reported human remain specimen numbers and complete repository information, including museum name and geographic location.
If permits were required, please ensure that you have provided details for all permits that were obtained, including the full name of the issuing authority, and add the following statement:
'All necessary permits were obtained for the described study, which complied with all relevant regulations.'
If no permits were required, please include the following statement:
'No permits were required for the described study, which complied with all relevant regulations.'
For more information on PLOS ONE's requirements for paleontology and archeology research, see https://journals.plos.org/plosone/s/submission-guidelines#loc-paleontology-and-archaeology-research.

Completed

3. Please take this opportunity to be sure you have met all of our guidelines for new species. For proper registration of a new zoological taxon, we require two specific statements to be included in your manuscript.
1) In the Results section, the globally unique identifier (GUID), currently in the form of a Life Science Identifier (LSID), should be listed under the new species name, for example:

Anochetus boltoni Fisher sp. nov. urn:lsid:zoobank.org:act:B6C072CF-1CA6-40C7-8396-534E91EF7FBB
Another LSID for the manuscript itself should also appear within the Nomenclature statement. You will need to contact Zoobank (zoobank.org/About) to obtain a GUID (LSID). You should receive one LSID for your manuscript and a separate, unique LSID for the new species.

Completed

2) Please also insert the following text into the Methods section, in a sub-section to be called ""Nomenclatural Acts"":

Completed

The electronic edition of this article conforms to the requirements of the amended International Code of Zoological Nomenclature, and hence the new names contained herein are available under that Code from the electronic edition of this article. This published work and the nomenclatural acts it contains have been registered in ZooBank, the online registration system for the ICZN. The ZooBank LSIDs (Life Science Identifiers) can be resolved and the associated information viewed through any standard web browser by appending the LSID to the prefix ""http://zoobank.org/"".
The LSID for this publication is: urn:lsid:zoobank.org:pub: XXXXXXX.

The electronic edition of this work was published in a journal with an ISSN, and has been archived and is available from the following digital repositories: PubMed Central, LOCKSS [author to insert any additional repositories].

Completed

All PLOS ONE articles are deposited in PubMed Central and LOCKSS. If your institute, or those of your co-authors, has its own repository, we recommend that you also deposit the published online article there and include the name in your article.

Following a recent ruling by the International Commission on Zoological Nomenclature, electronic journals are now a valid format for publication of new zoological taxa. In order to ensure the valid publication of your new species, please be sure to include the updated version of Nomenclatural Acts (above).

A complete explanation of our guidelines for publishing new species can be found on our website: http://www.plosone.org/static/guidelines#zoological.

4. When completing the data availability statement of the submission form, you indicated that you will make your data available on acceptance. We strongly recommend all authors decide on a data sharing plan before acceptance, as the process can be lengthy and hold up publication timelines. Please note that, though access restrictions are acceptable now, your entire data will need to be made freely accessible if your manuscript is accepted for publication. This policy applies to all data except where public deposition would breach compliance with the protocol approved by your research ethics board. If you are unable to adhere to our open data policy, please kindly revise your statement to explain your reasoning and we will seek the editor's input on an exemption. Please be assured that, once you have provided your new statement, the assessment of your exemption will not hold up the peer review process.

Completed

5. Please amend either the title on the online submission form (via Edit Submission) or the title in the manuscript so that they are identical.

Completed

6. We are unable to open your Supporting Information file [AvisaurusTMT_Rcode (for journal).R]. Please kindly revise as necessary and re-upload.

Completed

7. Please review your reference list to ensure that it is complete and correct. If you have cited papers that have been retracted, please include the rationale for doing so in the manuscript text, or remove these references and replace them with relevant current references. Any changes to the reference list should be mentioned in the rebuttal letter that accompanies your revised manuscript. If you need to cite a retracted article, indicate the article's retracted status in the References list and also include a citation and full reference for the retraction notice.

Completed

Reviewer #1: Clark et al. describe three new specimens of avisaurids birds, and use them to support the existing hypothesis that avisaurids were raptorial birds. Attached to my review is a PDF of the manuscript with comments made directly.

The comments made throughout the manuscript by the reviewer were both helpful and productive, and we thank the reviewer for their advice and assistance in creating a stronger manuscript. We found the section concerning the mass ranges of birds particularly helpful and constructive. We have made sure that our wording is clearer when referring to a particular group or when referring to the higher percentile range of that particular group [i.e., we have made it clear when referring to a portion of a family ("larger members") or to the family as a whole]. We have also included better and more specific sources for where size ranges are sourced from.

All comments or suggestions made by the reviewer which required a simple fix or alteration have been corrected throughout the manuscript and figures. There were a number of points brought up within

the manuscript notes that we will address here. Lastly, we have also revised all figures as requested including figure 6, 8, 11 and included a new supplemental figure with better images of the proximoplantar fossa as requested (Fig. S2).

Comparison with penguins: We removed the brief section in the discussion that covered comparisons with penguins as requested. Originally, we wanted to include a brief section comparing this group as we felt that including them made sense given the superficially similar aspect ratios. However, as commented on, the manuscript does not require this section and it has since been removed to make the discussion more streamline.

Pengornathids as late-diverging enantiornthines: Thank you for pointing this phylogenetic result out! Our results do differ from those in Atterholt et al., (2018), but pengornithids are recovered as late-diverging enantiornithines in other published literature as well:

O'Connor et al., 2013 - 10.1080/02724634.2012.719176
Wang et al., 2022 - 10.1017/jpa.2022.12
Li et al., 2021 - 10.1111/joa.13588
O'Conner et al., 2020 - 10.1038/s41586-020-2945-x

Introduction comment: We find that the section within the introduction which discusses previous criteria for the Avisauridae is needed both for the reader to understand the history of avisaurids, and for the introduction to be a proper review of previous literature. For readers with limited knowledge of this clade of enantiornithines, we find that introducing these topics (discussing prior synapomorphies) within the introduction to be important and relevant. By discussing these taxa within the introduction, we feel that we have given the reader a thorough and relevant summary of the current state of this clade of enantiornithines. Additionally, we briefly cover why there are initial issues with previous criteria for admittance to this clade. With this section in the introduction, it allows the reader to see that prior criteria for consideration into the Avisauridae requires an update – which is something we state as an objective in the introduction, and also why some taxa are not considered to be avisaurids here.

Figure 6: With regards to additional work for figure 6 (regression analysis): We have made sure to include both slope and resulting R^2 values for both A and B in figure 6 (and for figure S1 were requested). However, we find that phylogenetically influenced results when dealing with enantiornithines can lead to further confusion given that the phylogeny of this entire clade is still highly debated. Our objective in this figure is to only direct the reader to the fact that over time, as far as we can tell from the fossil record, enantiornithine body size remains fairly conserved early in their evolution and expands to both lower and higher thresholds by the end Cretaceous.

Line 545 (relating to lowering aspect ratio equating to greater efficiency) "This is untrue. Chiefly, a lower aspect ratio does not inherently affect the relative length of the tmt. Aspect ratio chiefly describes the relative length and width within the tmt, but not its muscle tendon insertion location nor its length relative to any other bone in the hindlimb. If an accipitrid had its tmt midshaft stretched laterally its aspect ratio would reduce, but the tmt would still be just as long and have the same fulcrum, moment of action, and resistance locations."

In birds, we typically do see that lower aspect ratio tarsometatarsi do change the proportional lengths against the tibiotarsus. Additionally, when the aspect ratio diminishes, particularly in birds of prey, we also see an increase in the proportional size (i.e., surface area) of the tubercle. In the hypothetical example given by the reviewer (stretching a hawk's tarsometatarsus), we would expect, based on all other birds of prey, that the relative position and surface area of the tubercle would also change, creating a whole different lever system. If the tarsometatarsus was stretched mediolaterally, it would alter how the hindlimb works and in reality, other parts of the system would change. To the point, we see this very

thing happen but in reverse in the Barn owl (*Tyto alba*). Barn owls have essentially taken the owl-like tarsometatarsus and increased the aspect ratio, elongating it. The result is not little change to the lever system, but alternatively, it has taken the form of a much more hawk-like tarsometatarsus, resulting in an altogether different lever system than other strigiforms. In this relevant example, by changing the aspect ratio, you change the relative length compared to the tibiotarsus, and the position of the tubercle. These results are actually forthcoming in another manuscript soon to be submitted focused on accipitrid and strigid tarsometatarsi.

We find that changes in aspect ratios do in fact change relative location of the tubercle and the lever system as a whole. Lastly, by lowering the aspect ratio, you inherently reduce the proportional length against the other hindlimb bones among birds. Aspect ratio does not scale proportionally among birds of prey – which is made evident in owls and hawks assuming different methods of prey acquisition. Again, when a member of either order evolves aspect ratios closer to the other order, it then exhibits prey acquisition strategies closer to the latter.

Comment: "Perhaps the most obvious association with a higher aspect ratio goes unmentioned, though: Higher resistance to bending forces. As elongate bones thicken in a dimension perpendicular to their long axis they tends to better resist bending forces (see e.g. Cuff and Rayfield). So a tmt with a relatively low aspect ratio will be relatively better able to resist forces like those in locomotion and hunting".

Thank you for bringing this point up. This thought did occur to us during our initial research, however, a key feature that differs between neornithines and enantiornithines kept this point from our discussion. In neornithines rigidity of the foot is far better facilitated as they have fully fused metatarsals while enantiornithines do not. Even robust avisaurids still retain the enantiornithine feature of fusion limited to the proximal portion. Torsion of the individual metatarsals would be more likely for enantiornithines compared to extant birds of prey today. However, as the reviewer states, this point is debatable. However, we feel it would not contribute enough to be included in our discussion due to the high level of uncertainty regarding rigidity in unfused metatarsals.

Owl vs hawk comments: There appeared to a few points of misunderstanding with the reviewer that we believe were both addressed in the original draft and are further clarified in the revised manuscript. Particularly with the comments addressing lines 539 and 545 (above), we felt that we made it clear that avisaurids did not function either as owls or as accipitrids in their given environment, but they do display morphological similarities to both groups, suggesting a unique morphological solution to perhaps a similar predatory niche. We lean more so into strigids throughout our discussion because they show more similarities with the lower aspect ratio of the tarsometatarsus and the subsequent lever system implications. However, we have made sure throughout the revised manuscript to state that we are drawing from strigid morphology, not stating it as a direct analogue for how avisaurids functioned. We have more our discussion points, including the ability to lift proportionally heavy things, clearer and address limitations of increasing size. Thank you for making this clearer.

Line 553. "These are not just driven by the low aspect ratio of the tmt, though. The cranial face of owl tarsometatarsi have deep excavations that run down nearly half the length for additional adductor muscle attachment area, and the plantar side is even more dramatically incised to accommodate the large flexor muscles. While the low aspect ratio of the avisaurid tmt does allow for relatively broader muscle attachments than other enantiornithines, it seems unlikely to me that gripping abilities analagous to owls can be attributed to Avisauridae."

We agree with the reviewer; however, we do not state that avisaurids had the gripping abilities as owls – only that they were likely increased relative to other enantiornithines (which we have clarified in the text). We have made sure to include words such as "analogous" to strigid grip where requested in the manuscript. The objective of this portion of the discussion was to signal that owls have morphologies that allow for increased grip relative to accipitrids. In the same way, avisaurids show similar, uniquely-strigid reminiscent morphologies.

Fig. 9 comment: “This is likely more an effect of body size than musculature. Lift scales with wing area and muscle cross-sectional area, both of which scale to body mass^2/3. As such, larger raptorial birds tend to either hunt relatively less massive prey or to kill prey on the ground without lifting it. The Northern pygmy owl in the figure has an average mass of around 70 g, less than a tenth of mass estimates for A. darwini. So feats like this would be unlikely for the avisaurid.”

We agree with the reviewer. Assuredly, a bird the size of a large avisaurid could not lift items nearly three times its own weight (similar to much smaller strigids). However, the biomechanics for increased compensation for carrying weight are present. We have made sure to make this clear in the figure’s description and the discussion/conclusion sections.

Line 689: “Pedal phalanges are known from Mirarce. Do they have similar aspect ratios to owls'? To other raptorial birds?”

This will be discussed in a forthcoming manuscript! Thank you for pointing it out. For this manuscript we wanted to remain fixed on morphology and function of the tarsometatarsus (which includes the deeply grooved trochlea).

The descriptive portions of the manuscript are well-executed. Morphology is detailed and comparisons to establish new species are appropriate. I have requested some more detail on the Methods, but many of these requests could easily be met with a supplemental file.

Thank you again for your constructive comments throughout the manuscript. I am not sure why the supplemental tables were unavailable to you, but we have made sure to upload them again in csv format this time so that the R code will read them correctly.

However, there are some issues in the authors’ evidence presented for avisaurids as raptorial birds. In particular, the authors place much emphasis on the similarity between avisaurids and owls. In doing so, they ignore several major differences between owls and avisaurids and cite several similarities also shared with other raptorial birds. The authors mention briefly in the Abstract and Conclusion that avisaurids likely inhabited a raptorial niche not reminiscent of any extant group, and I believe this hypothesis has more support than using owls as analogues and should be more fleshed-out in the body of the manuscript.

(see above comment). Thank you again for pressing on this central point of our manuscript. We have made sure to clarify our points both as directed and where needed.

Finally, most of the underlying data for this study has not been provided. None of the supplemental tables nor the .csv files which the uploaded R code relies on were a part of the submission (at least not a part I could access).

(see above comment). We apologize for this issue. All files were previosuly uploaded correctly. However, we have reuploaded all supplemental files in the correct way according to the PLOS one submission system. Additionally, we have changed all excel files to .csv and altered the Rcode to better read these files. We hope that all uploads function correctly during the next revision. Thank you.

Overall, I believe this work is publishable with relatively minor revisions (including all supplemental files, more detailed Methods, removing commented inaccuracies from Discussion and lessening emphasis on owls as full analogues) but can be made much more impactful with more major revisions (building a more cohesive argument around Avisauridae’s unique raptorial niche).

Thank you very much again for your suggestions and constructive points.

The authors' work is both interesting and well-executed and would be an excellent addition to PLOS ONE.

Reviewer #2: The manuscript is in good shape and ready for publication. I have only a few comments:

1- I wouldn't call "Williams Quarry" a Lagerstatte. There is no evidence of soft tissue preserved or any type of other exceptional type of preservation. I believe WQ is more appropriately referred to as a 'bonebed'. You may want to also cite a recent paper on a basicranium from WQ as this paper refers to the discovery of over 1000 enantiornithine bones at this quarry (Chiappe LM, et al 2022. Proc. R. Soc. B 289: 20221398. https://doi.org/10.1098/rspb.2022.1398)

Thank you for the correction. This has been revised in the manuscript to read as “a productive bonebed” rather than a Lagerstatte. The new reference has also been cited.

2. The passage in lines 446-448 is confusing. The authors say that "Currently, the smallest Upper Cretaceous
enantiornithines are Burmese amber specimens (Elektorornis, approximately the size of 3 g extant trochilids) and the largest is the holotype of Pengornis houi [similar in size to large psittaciforms (600-448 750 grams) (Zhou et al., 2008)]." However Pengornis is an early Cretaceous taxon--please revise this section, and note that Chiappe (among others) highlighted the fact that Avisaurids are relatively large birds. This is stated on several papers, including those cited in this papers.

The amber specimens are younger than 100.5 My, making them Upper Cretaceous specimens.

3. The discussion about a trend of increased body size during the evolutionary history of enantiornithines needs a bit more of historical context, as the authors--based on the references they cite--make it look as this has only been noticed recently. In fact, almost 30 years ago (e.g. Chiappe 1996) this trend was clearly noticed together with the fact that the entire spectrum of body size increased significantly towards the Late Cretaceous. As an example, I'm including a section from Chiappe & Walker (2002), a paper already cited by the authors:

"During their evolution throughout the Cretaceous there is a general increment in size and size range among the eu- enantiornithine species. All known Early Cretaceous eu- enantiornithines are small to medium-size birds (see com- parative measurements in Bochenski, 1996). Sinornis and Eoalulavis, for example, were the size of a sparrow or finch, and the size of Nanantius and Concornis was comparable to that of a medium-sized thrush. Late Cretaceous euenan- tiornithines, however, are generally larger: Gobipteryx was the size of a jackdaw and Neuquenornis that of a small fal- con. Some of the Late Cretaceous taxa were much larger. Enantiornis had a wingspan of about 1.2 meters, ranging in size between a skua and a turkey vulture (Chiappe, 1996b), and a recently discovered enantiornithine from France has been compared in size to a herring gull (Buffetaut, 1998). Some Late Cretaceous euenantiornithines, however, re- tained the small size of their Early Cretaceous relatives; Alexornis was sparrow-sized."

Corrected. We have made sure to acknowledge that this trend has been previously recognized. Thank you for the correction
