# Supplementary material for: New enantiornithine diversity in the Hell Creek Formation and the functional morphology of the avisaurid tarsometatarsus
Source: PLoS One. 2024 Oct 9;19(10):e0310686. doi: 10.1371/journal.pone.0310686 (PMC11463745; doi:10.1371/journal.pone.0310686)
Supplement: Attachment — Submitted filename: Response to Reviewers.docx [file pone.0310686.s009.docx]

We would like to thank the editor, and both reviewers for their very constructive and helpful suggestions for our manuscript. We find in having incorporated them and making other suggested changes, that we have made our manuscript more robust and clearer concerning our conclusions and lines of reasoning. Thank you for your contributions to our manuscript.

Sincerely,

Alex Clark (corresponding author)

**Editor**

1. Please ensure that your manuscript meets PLOS ONE's style requirements, including those for file naming. The PLOS ONE style templates can be found at

<https://journals.plos.org/plosone/s/file?id=wjVg/PLOSOne_formatting_sample_main_body.pdf> and 
<https://journals.plos.org/plosone/s/file?id=ba62/PLOSOne_formatting_sample_title_authors_affiliations.pdf>

Completed

2. In your manuscript, please provide additional information regarding the specimens used in your study. Ensure that you have reported human remain specimen numbers and complete repository information, including museum name and geographic location.

If permits were required, please ensure that you have provided details for all permits that were obtained, including the full name of the issuing authority, and add the following statement:

'All necessary permits were obtained for the described study, which complied with all relevant regulations.'

If no permits were required, please include the following statement:

'No permits were required for the described study, which complied with all relevant regulations.'

For more information on PLOS ONE's requirements for paleontology and archeology research, see <https://journals.plos.org/plosone/s/submission-guidelines#loc-paleontology-and-archaeology-research>.

Completed

3. Please take this opportunity to be sure you have met all of our guidelines for new species. For proper registration of a new zoological taxon, we require two specific statements to be included in your manuscript.

1) In the Results section, the globally unique identifier (GUID), currently in the form of a Life Science Identifier (LSID), should be listed under the new species name, for example:
 
Anochetus boltoni Fisher sp. nov. urn:lsid:[zoobank.org](http://zoobank.org):act:B6C072CF-1CA6-40C7-8396-534E91EF7FBB
Another LSID for the manuscript itself should also appear within the Nomenclature statement. You will need to contact Zoobank ([zoobank.org/About](http://zoobank.org/About)) to obtain a GUID (LSID). You should receive one LSID for your manuscript and a separate, unique LSID for the new species.

Completed

2) Please also insert the following text into the Methods section, in a sub-section to be called ""Nomenclatural Acts"":
 	Completed
The electronic edition of this article conforms to the requirements of the amended International Code of Zoological Nomenclature, and hence the new names contained herein are available under that Code from the electronic edition of this article. This published work and the nomenclatural acts it contains have been registered in ZooBank, the online registration system for the ICZN. The ZooBank LSIDs (Life Science Identifiers) can be resolved and the associated information viewed through any standard web browser by appending the LSID to the prefix ""<http://zoobank.org/>"".

The LSID for this publication is: urn:lsid:[zoobank.org](http://zoobank.org):pub: XXXXXXX.

The electronic edition of this work was published in a journal with an ISSN, and has been archived and is available from the following digital repositories: PubMed Central, LOCKSS [author to insert any additional repositories].
 	Completed
All PLOS ONE articles are deposited in PubMed Central and LOCKSS. If your institute, or those of your co-authors, has its own repository, we recommend that you also deposit the published online article there and include the name in your article.

Following a recent ruling by the International Commission on Zoological Nomenclature, electronic journals are now a valid format for publication of new zoological taxa. In order to ensure the valid publication of your new species, please be sure to include the updated version of Nomenclatural Acts (above).

A complete explanation of our guidelines for publishing new species can be found on our website: <http://www.plosone.org/static/guidelines#zoological>.

4. When completing the data availability statement of the submission form, you indicated that you will make your data available on acceptance. We strongly recommend all authors decide on a data sharing plan before acceptance, as the process can be lengthy and hold up publication timelines. Please note that, though access restrictions are acceptable now, your entire data will need to be made freely accessible if your manuscript is accepted for publication. This policy applies to all data except where public deposition would breach compliance with the protocol approved by your research ethics board. If you are unable to adhere to our open data policy, please kindly revise your statement to explain your reasoning and we will seek the editor's input on an exemption. Please be assured that, once you have provided your new statement, the assessment of your exemption will not hold up the peer review process.

Completed

5. Please amend either the title on the online submission form (via Edit Submission) or the title in the manuscript so that they are identical.

Completed

6. We are unable to open your Supporting Information file [AvisaurusTMT_Rcode (for journal).R]. Please kindly revise as necessary and re-upload.

Completed

7. Please review your reference list to ensure that it is complete and correct. If you have cited papers that have been retracted, please include the rationale for doing so in the manuscript text, or remove these references and replace them with relevant current references. Any changes to the reference list should be mentioned in the rebuttal letter that accompanies your revised manuscript. If you need to cite a retracted article, indicate the article’s retracted status in the References list and also include a citation and full reference for the retraction notice.

Completed

**Reviewer #1:** Clark et al. describe three new specimens of avisaurids birds, and use them to support the existing hypothesis that avisaurids were raptorial birds. Attached to my review is a PDF of the manuscript with comments made directly.

The comments made throughout the manuscript by the reviewer were both helpful and productive, and we thank the reviewer for their advice and assistance in creating a stronger manuscript. We found the section concerning the mass ranges of birds particularly helpful and constructive. We have made sure that our wording is clearer when referring to a particular group or when referring to the higher percentile range of that particular group [i.e., we have made it clear when referring to a portion of a family (“larger members”) or to the family as a whole]. We have also included better and more specific sources for where size ranges are sourced from.

All comments or suggestions made by the reviewer which required a simple fix or alteration have been corrected throughout the manuscript and figures. There were a number of points brought up within the manuscript notes that we will address here. Lastly, we have also revised all figures as requested including figure 6, 8, 11 and included a new supplemental figure with better images of the proximoplantar fossa as requested (Fig. S2).

Comparison with penguins: We removed the brief section in the discussion that covered comparisons with penguins as requested. Originally, we wanted to include a brief section comparing this group as we felt that including them made sense given the superficially similar aspect ratios. However, as commented on, the manuscript does not require this section and it has since been removed to make the discussion more streamline.

Pengornathids as late-diverging enantiornthines: Thank you for pointing this phylogenetic result out! Our results do differ from those in Atterholt et al., (2018), but pengornithids are recovered as late-diverging enantiornithines in other published literature as well:

O’Connor et al., 2013 - 10.1080/02724634.2012.719176

Wang et al., 2022 - 10.1017/jpa.2022.12

Li et al., 2021 - 10.1111/joa.13588

O’Conner et al., 2020 - 10.1038/s41586-020-2945-x

Introduction comment: We find that the section within the introduction which discusses previous criteria for the Avisauridae is needed both for the reader to understand the history of avisaurids, and for the introduction to be a proper review of previous literature. For readers with limited knowledge of this clade of enantiornithines, we find that introducing these topics (discussing prior synapomorphies) within the introduction to be important and relevant. By discussing these taxa within the introduction, we feel that we have given the reader a thorough and relevant summary of the current state of this clade of enantiornithines. Additionally, we briefly cover why there are initial issues with previous criteria for admittance to this clade. With this section in the introduction, it allows the reader to see that prior criteria for consideration into the Avisauridae requires an update – which is something we state as an objective in the introduction, and also why some taxa are not considered to be avisaurids here.

Figure 6: With regards to additional work for figure 6 (regression analysis): We have made sure to include both slope and resulting R^2^ values for both A and B in figure 6 (and for figure S1 were requested). However, we find that phylogenetically influenced results when dealing with enantiornithines can lead to further confusion given that the phylogeny of this entire clade is still highly debated. Our objective in this figure is to only direct the reader to the fact that over time, as far as we can tell from the fossil record, enantiornithine body size remains fairly conserved early in their evolution and expands to both lower and higher thresholds by the end Cretaceous.

Line 545 (relating to lowering aspect ratio equating to greater efficiency) “This is untrue. Chiefly, a lower aspect ratio does not inherently affect the relative length of the tmt. Aspect ratio chiefly describes the relative length and width within the tmt, but not its muscle tendon insertion location nor its length relative to any other bone in the hindlimb. If an accipitrid had its tmt midshaft stretched laterally its aspect ratio would reduce, but the tmt would still be just as long and have the same fulcrum, moment of action, and resistance locations.”

In birds, we typically do see that lower aspect ratio tarsometatarsi do change the proportional lengths against the tibiotarsus. Additionally, when the aspect ratio diminishes, particularly in birds of prey, we also see an increase in the proportional size (i.e., surface area) of the tubercle. In the hypothetical example given by the reviewer (stretching a hawk’s tarsometatarsus), we would expect, based on all other birds of prey, that the relative position and surface area of the tubercle would also change, creating a whole different lever system. If the tarsometatarsus was stretched mediolaterally, it would alter how the hindlimb works and in reality, other parts of the system would change. To the point, we see this very thing happen but in reverse in the Barn owl (*Tyto* *alba*). Barn owls have essentially taken the owl-like tarsometatarsus and increased the aspect ratio, elongating it. The result is not little change to the lever system, but alternatively, it has taken the form of a much more hawk-like tarsometatarsus, resulting in an altogether different lever system than other strigiforms. In this relevant example, by changing the aspect ratio, you change the relative length compared to the tibiotarsus, and the position of the tubercle. These results are actually forthcoming in another manuscript soon to be submitted focused on accipitrid and strigid tarsometatarsi.

We find that changes in aspect ratios do in fact change relative location of the tubercle and the lever system as a whole. Lastly, by lowering the aspect ratio, you inherently reduce the proportional length against the other hindlimb bones among birds. Aspect ratio does not scale proportionally among birds of prey – which is made evident in owls and hawks assuming different methods of prey acquisition. Again, when a member of either order evolves aspect ratios closer to the other order, it then exhibits prey acquisition strategies closer to the latter.

Comment: “Perhaps the most obvious association with a higher aspect ratio goes unmentioned, though: Higher resistance to bending forces. As elongate bones thicken in a dimension perpendicular to their long axis they tends to better resist bending forces (see e.g. Cuff and Rayfield). So a tmt with a relatively low aspect ratio will be relatively better able to resist forces like those in locomotion and hunting”.

Thank you for bringing this point up. This thought did occur to us during our initial research, however, a key feature that differs between neornithines and enantiornithines kept this point from our discussion. In neornithines rigidity of the foot is far better facilitated as they have fully fused metatarsals while enantiornithines do not. Even robust avisaurids still retain the enantiornithine feature of fusion limited to the proximal portion. Torsion of the individual metatarsals would be more likely for enantiornithines compared to extant birds of prey today. However, as the reviewer states, this point is debatable. However, we feel it would not contribute enough to be included in our discussion due to the high level of uncertainty regarding rigidity in unfused metatarsals.

Owl vs hawk comments: There appeared to a few points of misunderstanding with the reviewer that we believe were both addressed in the original draft and are further clarified in the revised manuscript. Particularly with the comments addressing lines 539 and 545 (above), we felt that we made it clear that avisaurids did not function either as owls or as accipitrids in their given environment, but they do display morphological similarities to both groups, suggesting a unique morphological solution to perhaps a similar predatory niche. We lean more so into strigids throughout our discussion because they show more similarities with the lower aspect ratio of the tarsometatarsus and the subsequent lever system implications. However, we have made sure throughout the revised manuscript to state that we are drawing from strigid morphology, not stating it as a direct analogue for how avisaurids functioned. We have more our discussion points, including the ability to lift proportionally heavy things, clearer and address limitations of increasing size. Thank you for making this clearer.

Line 553. “These are not just driven by the low aspect ratio of the tmt, though. The cranial face of owl tarsometatarsi have deep excavations that run down nearly half the length for additional adductor muscle attachment area, and the plantar side is even more dramatically incised to accommodate the large flexor muscles. While the low aspect ratio of the avisaurid tmt does allow for relatively broader muscle attachments than other enantiornithines, it seems unlikely to me that gripping abilities analagous to owls can be attributed to Avisauridae.”

We agree with the reviewer; however, we do not state that avisaurids had the gripping abilities as owls – only that they were likely increased relative to other enantiornithines (which we have clarified in the text). We have made sure to include words such as “analogous” to strigid grip where requested in the manuscript. The objective of this portion of the discussion was to signal that owls have morphologies that allow for increased grip relative to accipitrids. In the same way, avisaurids show similar, uniquely-strigid reminiscent morphologies.

Fig. 9 comment: “This is likely more an effect of body size than musculature. Lift scales with wing area and muscle cross-sectional area, both of which scale to body mass^2/3. As such, larger raptorial birds tend to either hunt relatively less massive prey or to kill prey on the ground without lifting it. The Northern pygmy owl in the figure has an average mass of around 70 g, less than a tenth of mass estimates for A. darwini. So feats like this would be unlikely for the avisaurid.”

We agree with the reviewer. Assuredly, a bird the size of a large avisaurid could not lift items nearly three times its own weight (similar to much smaller strigids). However, the biomechanics for increased compensation for carrying weight are present. We have made sure to make this clear in the figure’s description and the discussion/conclusion sections.

Line 689: “Pedal phalanges are known from Mirarce. Do they have similar aspect ratios to owls'? To other raptorial birds?”

This will be discussed in a forthcoming manuscript! Thank you for pointing it out. For this manuscript we wanted to remain fixed on morphology and function of the tarsometatarsus (which includes the deeply grooved trochlea).

The descriptive portions of the manuscript are well-executed. Morphology is detailed and comparisons to establish new species are appropriate. I have requested some more detail on the Methods, but many of these requests could easily be met with a supplemental file.

Thank you again for your constructive comments throughout the manuscript. I am not sure why the supplemental tables were unavailable to you, but we have made sure to upload them again in csv format this time so that the R code will read them correctly.

However, there are some issues in the authors’ evidence presented for avisaurids as raptorial birds. In particular, the authors place much emphasis on the similarity between avisaurids and owls. In doing so, they ignore several major differences between owls and avisaurids and cite several similarities also shared with other raptorial birds. The authors mention briefly in the Abstract and Conclusion that avisaurids likely inhabited a raptorial niche not reminiscent of any extant group, and I believe this hypothesis has more support than using owls as analogues and should be more fleshed-out in the body of the manuscript.

(see above comment). Thank you again for pressing on this central point of our manuscript. We have made sure to clarify our points both as directed and where needed.

Finally, most of the underlying data for this study has not been provided. None of the supplemental tables nor the .csv files which the uploaded R code relies on were a part of the submission (at least not a part I could access).

(see above comment). We apologize for this issue. All files were previosuly uploaded correctly. However, we have reuploaded all supplemental files in the correct way according to the PLOS one submission system. Additionally, we have changed all excel files to .csv and altered the Rcode to better read these files. We hope that all uploads function correctly during the next revision. Thank you.

Overall, I believe this work is publishable with relatively minor revisions (including all supplemental files, more detailed Methods, removing commented inaccuracies from Discussion and lessening emphasis on owls as full analogues) but can be made much more impactful with more major revisions (building a more cohesive argument around Avisauridae’s unique raptorial niche).

Thank you very much again for your suggestions and constructive points.

The authors' work is both interesting and well-executed and would be an excellent addition to PLOS ONE.

**Reviewer #2**: The manuscript is in good shape and ready for publication. I have only a few comments:

1- I wouldn't call "Williams Quarry" a Lagerstatte. There is no evidence of soft tissue preserved or any type of other exceptional type of preservation. I believe WQ is more appropriately referred to as a 'bonebed'. You may want to also cite a recent paper on a basicranium from WQ as this paper refers to the discovery of over 1000 enantiornithine bones at this quarry (Chiappe LM, et al 2022. Proc. R. Soc. B 289: 20221398. <https://doi.org/10.1098/rspb.2022.1398>)

Thank you for the correction. This has been revised in the manuscript to read as “a productive bonebed” rather than a Lagerstatte. The new reference has also been cited.

2. The passage in lines 446-448 is confusing. The authors say that "Currently, the smallest Upper Cretaceous
enantiornithines are Burmese amber specimens (Elektorornis, approximately the size of 3 g extant
trochilids) and the largest is the holotype of Pengornis houi [similar in size to large psittaciforms (600-
448 750 grams) (Zhou et al., 2008)]." However Pengornis is an early Cretaceous taxon--please revise this section, and note that Chiappe (among others) highlighted the fact that Avisaurids are relatively large birds. This is stated on several papers, including those cited in this papers.

The amber specimens are younger than 100.5 My, making them Upper Cretaceous specimens.

3. The discussion about a trend of increased body size during the evolutionary history of enantiornithines needs a bit more of historical context, as the authors--based on the references they cite--make it look as this has only been noticed recently. In fact, almost 30 years ago (e.g. Chiappe 1996) this trend was clearly noticed together with the fact that the entire spectrum of body size increased significantly towards the Late Cretaceous. As an example, I'm including a section from Chiappe & Walker (2002), a paper already cited by the authors:

"During their evolution throughout the Cretaceous there is a general increment in size and size range among the eu- enantiornithine species. All known Early Cretaceous eu- enantiornithines are small to medium-size birds (see com- parative measurements in Bochenski, 1996). Sinornis and Eoalulavis, for example, were the size of a sparrow or ﬁnch, and the size of Nanantius and Concornis was comparable to that of a medium-sized thrush. Late Cretaceous euenan- tiornithines, however, are generally larger: Gobipteryx was the size of a jackdaw and Neuquenornis that of a small fal- con. Some of the Late Cretaceous taxa were much larger. Enantiornis had a wingspan of about 1.2 meters, ranging in size between a skua and a turkey vulture (Chiappe, 1996b), and a recently discovered enantiornithine from France has been compared in size to a herring gull (Buffetaut, 1998). Some Late Cretaceous euenantiornithines, however, re- tained the small size of their Early Cretaceous relatives; Alexornis was sparrow-sized."

Corrected. We have made sure to acknowledge that this trend has been previously recognized. Thank you for the correction
