# Supplementary material for: New enantiornithine diversity in the Hell Creek Formation and the functional morphology of the avisaurid tarsometatarsus
Source: PLoS One. 2024 Oct 9;19(10):e0310686. doi: 10.1371/journal.pone.0310686 (PMC11463745; doi:10.1371/journal.pone.0310686)
Supplement: Attachment — Submitted filename: Response to Reviewers.docx [file pone.0310686.s011.docx]

Dear Editor,

Thank you again for your assistance and throughout read-through of our manuscript. I have addressed your final comments here. Thank you again very much!

Sincerely,

Alex Clark

**Changes made:**

Thomas Carr was removed as an author – I have received confirmation from all authors that they responded to the journal in agreement of the change.

A reference has been added that was previously cited but missing the citation:

Schneider, C.A., Rasband, W.S., Eliceiri, K.W., 2012. NIH Image to ImageJ: 25 years of image analysis. Nat Methods 9, 671–675. https://doi.org/10.1038/nmeth.2089

Editor:

Please indicate which results are derived exclusively from the majority-rule tree. Personally, I have some reservations about accepting taxonomic decisions and reevaluations that are not well supported. It seems to me that some redefinitions are being based exclusively on the majority-rule tree, correct? Do the authors wish to maintain this approach? If so, I suggest indicating potential weaknesses inherent to this practice.

Thank you for pointing this out. We have since clarified this results section concerning characters, source of synapomorphies, and where similar morphological characters support inclusion within the clade.

I do believe that the correct plural term is Lagerstätten. Please change throughout.

According to a previous reviewer and internet sources, Lagerstätte is the plural. When entered into search fields as a plural noun, it is also corrected as Lagerstätte.
